# On the Ambiphilic Reactivity of Geometrically Constrained Phosphorus(III) and Arsenic(III) Compounds: Insights into Their Interaction with Ionic Substrates

**DOI:** 10.1002/chem.201603135

**Published:** 2016-09-15

**Authors:** Thomas P. Robinson, Siu‐Kwan Lo, Daniel De Rosa, Simon Aldridge, Jose M. Goicoechea

**Affiliations:** ^1^ Department of ChemistryUniversity of Oxford, Chemistry Research Laboratory12 Mansfield RoadOxfordOX1 3TAUK

**Keywords:** arsenic, phosphorus, reaction mechanism, structure elucidation

## Abstract

The ambiphilic nature of geometrically constrained Group 15 complexes bearing the *N*,*N*‐bis(3,5‐di‐*tert*‐butyl‐2‐phenolate)amide pincer ligand (ONO^3−^) is explored. Despite their differing reactivity towards nucleophilic substrates with polarised element–hydrogen bonds (e.g., NH_3_), both the phosphorus(III), P(ONO) (**1 a**), and arsenic(III), As(ONO) (**1 b**), compounds exhibit similar reactivity towards charged nucleophiles and electrophiles. Reactions of **1 a** and **1 b** with KO*t*Bu or KNPh_2_ afford anionic complexes in which the nucleophilic anion associates with the pnictogen centre ([(*t*BuO)Pn(ONO)]^−^ (Pn=P (**2 a**), As (**2 b**)) and [(Ph_2_N)Pn(ONO)]^−^ (Pn=P (**3 a**), As (**3 b**)). Compound **2 a** can subsequently be reacted with a proton source or benzylbromide to afford the phosphorus(V) compounds (*t*BuO)HP(ONO) (**4 a**) and (*t*BuO)BzP(ONO) (**5 a**), respectively, whereas analogous arsenic(V) compounds are inaccessible. Electrophilic substrates, such as HOTf and MeOTf, preferentially associate with the nitrogen atom of the ligand backbone of both **1 a** and **1 b**, giving rise to cationic species that can be rationalised as either ammonium salts or as amine‐stabilised phosphenium or arsenium complexes ([Pn{ON(H)O}]^+^ (Pn=P (**6 a**), As (**6 b**)) and [Pn{ON(Me)O}]^+^ (Pn=P (**7 a**), As (**7 b**)). Reaction of **1 a** with an acid bearing a nucleophilic counteranion (such as HCl) gives rise to a phosphorus(V) compound HPCl(ONO) (**8 a**), whereas the analogous reaction with **1 b** results in the addition of HCl across one of the As−O bonds to afford ClAs{(H)ONO} (**8 b**). Functionalisation at both the pnictogen centre and the ligand backbone is also possible by reaction of **7 a**/**7 b** with KO*t*Bu, which affords the neutral species (*t*BuO)Pn{ON(Me)O} (Pn=P (**9 a**), As (**9 b**)). The ambiphilic reactivity of these geometrically constrained complexes allows some insight into the mechanism of reactivity of **1 a** towards small molecules, such as ammonia and water.

## Introduction

Over the last decade significant advances have been made in the development of main‐group species that are capable of activating small molecules.^1^ Most prominent amongst these are alkyl(amino) carbenes (AACs),^2^ low oxidation state compounds of Groups 13 and 14,^3^, ^4^ and frustrated Lewis pairs (FLPs).^5^ Several of the aforementioned compounds have also shown to be active in catalytic processes, such as the hydrogenation of imines, alkenes and alkynes,^6^ effectively heralding a new era in the chemistry of the p‐block elements. It is also worth noting that the substrate scope of such compounds includes molecules such as ammonia, which has historically proven difficult to activate using precious metal catalysts due to the unfavourable coordination/activation equilibrium. New routes resulting in the activation of N−H bonds in ammonia are particularly appealing, because of the dearth of transition‐metal systems capable of effecting such a transformation,^7^, ^8^, ^9^, ^10^, ^11^, ^12^, ^13^, ^14^, ^15^, ^16^, ^17^, ^18^, ^19^ and the relevance of N−H activation to a number of potentially important industrial processes.^20^

**Figure 1:**
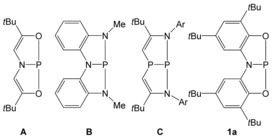
Selected geometrically constrained phosphorus(III) compounds capable of activating small molecules.

More recently, geometrically constrained compounds based on phosphorus(III) have also received significant attention in this field. Studies by Arduengo,^21^ Radosevich,^22^, ^23^, ^24^ Kinjo,^25^ and our own research group have demonstrated that the distorted T‐shaped phosphorus(III) compounds pictured in Figure 1 are capable of reacting with polar substrates such as water, alcohols, ammonia and amines.^26^ In the vast majority of cases, these reactions result in a formal oxidative addition of the substrate over the phosphorus(III) centre, although several theoretical studies have subsequently demonstrated that, in all likelihood, these processes involve the ligand backbone.^27^, ^28^, 1

We recently reported a phosphorus(III) compound bearing the *N*,*N*‐bis(3,5‐di‐*tert*‐butyl‐2‐phenolate)amide ligand (P(ONO); **1 a**).^26^ This species was found to react with ammonia and water, activating the E−H bonds in both substrates through a formal oxidative addition to afford the corresponding phosphorus(V) compounds. Under forcing condithereions (heating solid samples under a dynamic vacuum) we were able to show that these processes are reversible. During the course of these studies, we observed that **1 a** exclusively reacts with nucleophilic species with protic E−H bonds, whereas non‐nucleophilic substrates, even those with polar E−H bonds, such as phenylsilane, fail to react. These observations seemed to indicate that the nucleophilic association of the substrate is necessary prior to any further reactivity taking place. This prompted us to explore the reactivity of **1 a** towards both nucleophiles and electrophiles in an effort to gain a better understanding of the reaction dynamics. These results, and analogous studies on the heavier arsenic(III) analogue (**1 b**), are reported herein.

## Results and Discussion

### Structure and bonding considerations for 1 a and 1 b

As previously reported, **1 a** exhibits a bent geometry in the solid state with moderate pyramidalisation at both the phosphorus and nitrogen atoms (Σ_angles(P)_=296.1°; Σ_angles(N)_=331.6°), in contrast with planar compound **A**. Theoretical calculations at the density functional theory (DFT) level revealed that the pyramidal *C_s_* isomer (or electromorph to use the term coined by Arduengo) is the most stable, but relatively close in energy to the *C*_2*v*_ symmetric species (within 4 kJ mol^−1^). This energetic difference is within the error of the calculations, and it is worth noting that different computational analyses (varying basis sets and functionals) actually suggest that the planar structure is lower in energy than the *C_s_* isomer (albeit by similarly small values). Therefore, it is highly likely that, in solution, there is a dynamic, and concerted, pyramidal inversion at the phosphorus and nitrogen atoms resulting in a wing‐like "flapping" of the ligand backbone. The calculations also revealed that, regardless of the symmetry adopted by **1 a**, there is an empty, energetically accessible orbital that is largely based on the phosphorus atom, which has anti‐bonding character with respect to the P−N and P−O bonds. It is also worth noting that the P−N bond in **1 a** is significantly polarised, with computed Hirshfeld charges of 0.394 and −0.171 on the phosphorus and nitrogen atoms, respectively. These observations prompted us to explore the reactivity of **1 a**, and its heavier arsenic analogue (**1 b**), towards nucleophiles in order to establish whether they associate with the phosphorus/arsenic atom.

The arsenic‐containing species **1 b** can be prepared following a similar synthetic methodology to that previously reported for its lighter congener. Reaction of the protonated ligand, *N*,*N*‐bis(3,5‐di‐*tert*‐butyl‐2‐phenol)amine, H_3_ONO, with AsCl_3_ in the presence of three molar equivalents of triethylamine, affords **1 b** quantitatively, as evidenced by ^1^H and ^13^C{^1^H} NMR spectroscopy. The ^1^H NMR spectrum of **1 b** in C_6_D_6_ reveals two equal intensity aromatic resonances at 8.39 and 7.51 ppm as well as two singlets at 1.71 and 1.46 ppm arising from the *tert*‐butyl groups. Cooling of a concentrated pentane solution afforded bright red‐orange crystals of the compound in good to high yields.

**Figure 2:**
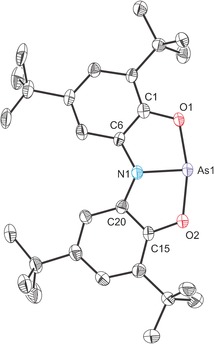
Molecular structure of **1 b** (thermal ellipsoids pictured at 50 % probability level; hydrogen atoms omitted for clarity). Selected interatomic distances [Å] and angles [°]: As1−N1 1.862(3), As1−O1 1.933(4), As1−O2 1.933(4), N1−C6 1.420(8), N1−C20 1.393(8), O1‐As1‐O2 164.39(12), N1‐As1‐O1 82.9(2), N1‐As1‐O2 81.8(2), C6‐N1‐C20 128.1(4), C6‐N1‐As1 115.2(4), C20‐N1‐As1 116.6(4).

The structure of **1 b** (Figure 2) exhibits a planar T‐shaped geometry about the arsenic(III) centre (mean deviation from As1/O1/N1/O2 plane is 0.0213 Å) with a near linear O1‐As1‐O2 angle of 164.39(12)°. The O‐As‐N angles, by contrast, are more acute and approach 90° (82.9(2) and 81.8(2)°), giving rise to a sum of bond angles around the arsenic centre (Σ_angles(As)_) of 329.1°. The sum of bond angles around the nitrogen centre, 359.9°, is significantly greater than for the lighter phosphorus‐containing analogue (331.6°). The As−O bond lengths (1.933(4) and 1.933(4) Å) are slightly elongated when compared to the expected values for single bonds (1.84–1.85 Å).^29^, ^30^ By contrast, the As−N bond (1.862(3) Å) is shorter than expected for a single bond (1.90–1.92 Å), indicating a significant degree of π‐donation from the nitrogen lone pair to the empty π‐orbital on the arsenic centre (and some multiple bond character due to this interaction). The structure and bond metrics for **1 b** are closely related to the planar 10‐As‐3 compound 5‐aza‐2,8‐dioxa‐l‐arsabicyclo[3.3.0]octa‐2,4,6‐triene previously reported by Arduengo and co‐workers (As−N: 1.839(3) Å; As−O: 1.955(3) and 1.998(3) Å; Σ_angles(As)_=320.7°).^21^ DFT calculations show a good agreement between the optimised geometry of **1 b** and that determined crystallographically. Interestingly, attempts to optimise the *C_s_* isomer of **1 b** ultimately converge to the planar (*C*_2*v*_) isomer, indicating that in contrast to its phosphorus‐containing analogue, the *C_s_* geometry of **1 b** is not a minimum on the potential energy hypersurface.2

### Reactivity of 1 a and 1 b towards nucleophiles

The reactivity of **1 a** and **1 b** was explored towards a number of nucleophilic species. In a preliminary report we demonstrated that **1 a** reacts with nucleophiles with polarised element–hydrogen bonds (NH_3_ and H_2_O) to give rise to five coordinate phosphorus(V) compounds. By contrast, no reaction is observed between **1 b** and ammonia, whereas hydrolysis does take place but gives rise to complex reaction mixtures and does not result in any arsenic(V)‐containing compounds. Our calculations reveal that this is largely a thermodynamic phenomenon. The reaction between NH_3_ and **1 a** is exothermic by 90 kJ mol^−1^, whereas the same reaction between NH_3_ and **1 b** is thermodynamically uphill by 86 kJ mol^−1^. This difference in reactivity can be attributed to the increased stabilisation of the “inert pair” on descending Group 15.

**Scheme 1:**
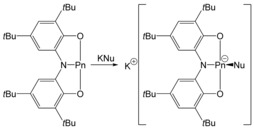
Reactivity of **1 a** towards nucleophiles (Pn=P, As; Nu=O*t*Bu, NPh_2_).

No reaction is observed for either **1 a** or **1 b** with neutral nucleophiles, such as pyridine or PPh_3_. In contrast, anionic nucleophiles do react with both compounds associating with the pnictogen centre. In a typical reaction, the geometrically constrained complexes were reacted with one equivalent of KNu (Nu=O*t*Bu or NPh_2_) in the presence of a cation sequestering agent (either 1,4,7,10,13,16‐hexaoxacyclooctadecane (18‐crown‐6) or 4,7,13,16,21,24‐hexaoxa‐1,10‐diazabicyclo[8.8.8]hexacosane (2,2,2‐crypt)) to aid crystallisation. These reactions yielded the anionic complexes [(*t*BuO)Pn(ONO)]^−^ (Pn=P (**2 a**) and As (**2 b**)) and [(Ph_2_N)Pn(ONO)]^−^ (Pn=P (**3 a**) and As (**3 b**)) as pictured in Scheme 1.

Reactions involving **1 a** were monitored by ^31^P NMR spectroscopy and show quantitative conversion to the desired products, which were observed as singlet resonances at 85.5 and 66.3 ppm for **2 a** and **3 a**, respectively. These resonances are shifted upfield with respect to **1 a** (168.6 ppm), due to the enhanced electron density on the phosphorus centre. Similarly the ^1^H NMR spectra of both compounds display the requisite number of resonances for a symmetrical *N*,*N*‐bis(3,5‐di‐*tert*‐butyl‐2‐phenolate)amide ligand backbone and for the nucleophilic substituents. Reactions involving **1 b** similarly give rise to products in which both of the 3,5‐di‐*tert*‐butyl‐2‐phenolate arms of the ligand backbone are equivalent as evidenced by ^1^H and ^13^C{^1^H} NMR spectroscopy.

**Figure 3:**
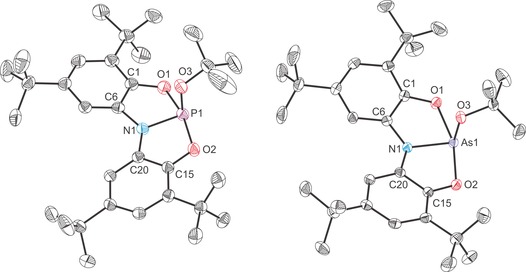
Molecular structures of [K(2,2,2‐crypt)][**2 a**]⋅1.5 tol (left) and [K(18‐crown‐6)][**2 b**]⋅THF (right). Thermal ellipsoids pictured at 50 % probability level; [K(2,2,2‐crypt)]^+^/[ K(18‐crown‐6)]^+^, solvent of crystallisation and hydrogen atoms omitted for clarity. Selected interatomic distances [Å] and angles [°]; **2 a**: P1−N1 1.759(2), P1−O1 1.830(1), P1−O2 1.978(1), P1−O3 1.652(1), O1‐P1‐O2 166.93(6), O1‐P1‐O3 93.79(7), O2‐P1‐O3 92.30(7), N1‐P1‐O1 84.96(6), N1‐P1‐O2 82.99(6), N1‐P1‐O3 95.03(7), C6‐N1‐C20 126.96(14), C6‐N1‐P1 114.19(11), C20‐N1‐P1 118.21(11). **2 b**: As1−N1 1.898(1), As1−O1 2.028(1), As1−O2 1.963(1), As1−O3 1.820(1), O1‐As1‐O2 162.95(4), O1‐As1‐O3 92.94(4), O2‐As1‐O3 93.31(4), N1‐As1‐O1 81.38(4), N1‐As1‐O2 82.40(4), N1‐As1‐O3 93.64(4), C6‐N1‐C20 127.76(10), C6‐N1‐As1 115.56(8), C20‐N1‐As1 114.11(8).

**Figure 4:**
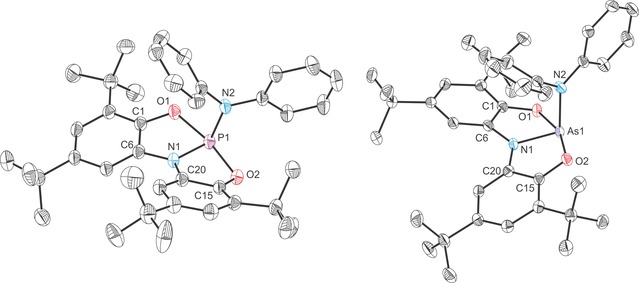
Molecular structures of [K(18‐crown‐6)][**3 a**]⋅0.5 tol⋅0.5 pent (left) and [K(2,2,2‐crypt)][**3 b**] (right). Thermal ellipsoids pictured at 50 % probability level; [K(18‐crown‐6)]^+^/[ K(2,2,2‐crypt)]^+^, solvent of crystallisation and hydrogen atoms omitted for clarity. Selected interatomic distances [Å] and angles [°]; **3 a**: P1−N1 1.749(2), P1−O1 1.855(2), P1−O2 1.931(2), P1−N2 1.764(2), O1‐P1‐O2 167.49(7), O1‐P1‐N2 91.52(8), O2‐P1‐N2 91.19(8), N1‐P1‐O1 84.83(8), N1‐P1‐O2 82.67(8), N1‐P1‐N2 103.90(9), C6‐N1‐C20 126.80(18), C6‐N1‐P1 115.99(15), C20‐N1‐P1 116.93(13). **3 b**: As1−N1 1.874(2), As1−O1 1.995(2), As1−O2 2.051(2), As1−N2 1.924(2), O1‐As1‐O2 161.48(6), O1‐As1‐N2 91.82(8), O2‐As1‐N2 92.84(8), N1‐As1‐O1 81.37(8), N1‐As1‐O2 80.22(8), N1‐As1‐N2 98.26(8), C6‐N1‐C20 128.26(18), C6‐N1‐As1 115.20(15), C20‐N1‐As1 116.51(14).

The structures of all four novel anionic complexes were determined by single‐crystal X‐ray diffraction (Figures 3 and 4). The complexes reveal planar Pn(ONO) moieties (mean deviation from plane for Pn1/O1/N1/O2: 0.0322 Å (**2 a**), 0.0365 Å (**2 b**) 0.0019 Å (**3 a**) and 0.0150 Å (**3 b**)) with the nucleophile orthogonal to the Pn(ONO) core. The planarity of the ligand backbone is also evident in the sum of bond angles around the nitrogen atoms (359.4, 357.4, 359.7 and 360.0° for **2 a**, **2 b**, **3 a** and **3 b**, respectively). These structures are consistent with lone‐pair donation from the nucleophile into the lowest unoccupied molecular orbital (LUMO) of **1 a** and **2 a**. DFT calculations reveal that the most significant atomic orbital contribution to this orbital comes from the pnictogen atom p orbital that is perpendicular to the plane of the molecule (53.75 and 49.97 % contributions for the phosphorus and arsenic p_*z*_ orbitals for **1 a** and **2 a**, respectively; the *z* axis is defined as orthogonal to the plane of the molecule). The LUMO of **1 a** and **2 a** are also notably p_π_–p_π_ antibonding with respect to the Pn−N and Pn−O bonds.3, 4

Upon coordination of an anionic nucleophile there is a significant elongation of the Pn−O bonds while the Pn−N bonds remain very similar to those of the parent compound (see Table 1 for a comparison of bond metric data for all complexes). In each of the adducts, the elongation of the Pn−O bonds is not uniform, but rather more pronounced for one of the two aryloxide functionalities of the *N*,*N*‐bis(3,5‐di‐*tert*‐butyl‐2‐phenolate)amide ligand. This significant weakening of the Pn−O bonds strongly suggests that on coordination of a nucleophile, the aryloxide functionalities are susceptible to electrophilic attack. This was probed by reacting **2 a** and **2 b** with a pyridinium trifluoromethanesulfonate and benzyl bromide (BzBr).

**Table 1:** Comparison of interatomic distances [Å] for compounds **1 a**–**3 a** and **1 b**–**3 b**.

	**1 a**	**2 a**	**3 a**	**1 b**	**2 b**	**3 b**
Pn−N	1.757(1)	1.759(2)	1.749(2)	1.862(3)	1.898(1)	1.874(2)
Pn−O	1.659(1)	1.830(1)	1.855(2)	1.933(4)	2.028(1)	1.995(2)
	1.652(1)	1.978(1)	1.931(2)	1.933(4)	1.963(1)	2.051(2)
Pn−Nu	N. A.	1.652(1)	1.764(2)	N.A.^[a]^	1.820(1)	1.924(2)

[a] N.A.=not applicable.

**Scheme 2:**
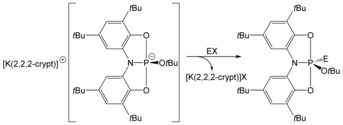
Formation of **4 a** and **5 a** from the reaction of [K(2,2,2‐crypt)][**2 a**] with pyridinium trifluoromethane sulfonate and benzyl bromide, respectively (E=PyH, X=OTf; E=Bz, X=Br).

Reactions of [K(2,2,2‐crypt)][**2 a**]⋅1.5 tol with one molar equivalent of pyridinium trifluoromethanesulfonate (PyHOTf) or benzyl bromide afforded the trigonal bipyramidal compounds (*t*BuO)HP(ONO) (**4 a**) and (*t*BuO)BzP(ONO) (**5 a**), respectively (Scheme 2). These novel phosphorus(V) compounds display similar structures and ^31^P NMR chemical shifts (−38.7 and −20.2 ppm for **4 a** and **5 a**, respectively) to the E−H activation products that were obtained by reaction of **1 a** with H_2_O and NH_3_. In fact, compound **4 a** can be readily accessed by direct reaction of **1 a** with HO*t*Bu. The reactions proceeded rapidly (before the ^31^P NMR spectra of the reaction mixtures were recorded) at room temperature affording the compounds in quantitative yields. Consequently, we were unable to ascertain whether electrophilic association of the proton or benzyl functionalities involved the ligand backbone prior to migration of the functional group to the phosphorus centre.

**Figure 5:**
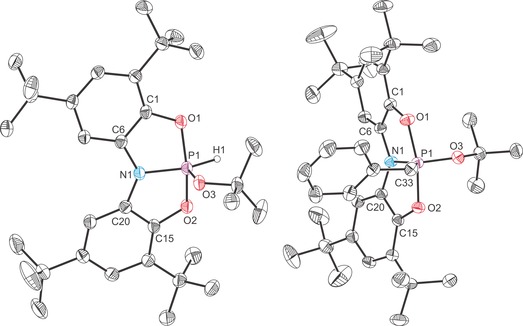
Molecular structures of **4 a** (left) and **5 a** (right). Thermal ellipsoids pictured at 50 % probability level; hydrogen atoms, with the exception of that bonded to phosphorus in **4 a**, omitted for clarity. There are two crystallographically independent molecules of **4 a** in the lattice, for clarity, only bond metric data for one of them is provided. Selected interatomic distances [Å] and angles [°]; **4 a**: P1−N1 1.720(1), P1−O1 1.688(1), P1−O2 1.681(1), P1−O3 1.588(1), P1−H1 1.30(2), O1‐P1‐O2 151.44(5), O1‐P1‐O3 104.26(5), O1‐P1‐H1 85.5(7), O2‐P1‐O3 104.29(5), O2‐P1‐H1 85.9(7), O3‐P1‐H1 106.5(7), N1‐P1‐O1 88.05(5), N1‐P1‐O2 88.00(5), N1‐P1‐O3 99.39(5), N1‐P1‐H1 154.2(7), C6‐N1‐C20 130.78(10), C6‐N1‐P1 114.31(8), C20‐N1‐P1 114.71(8). **5 a**: P1−N1 1.717(1), P1−O1 1.721(1), P1−O2 1.732(1), P1−O3 1.585(1), P1−C33 1.821(2), O1‐P1‐O2 168.15(5), O1‐P1‐O3 96.30(5), O1‐P1‐C33 88.05(5), O2‐P1‐O3 95.52(4), O2‐P1‐C33 89.87(5), O3‐P1‐C33 104.07(5), N1‐P1‐O1 86.74(4), N1‐P1‐O2 86.66(4), N1‐P1‐O3 119.96(5), N1‐P1‐C33 135.96(5), C6‐N1‐C20 128.50(10), C6‐N1‐P1 115.33(8), C20‐N1‐P1 116.06(8).

The structures of **4 a** and **5 a** were corroborated by single‐crystal X‐ray diffraction (Figure 5). Both display distorted trigonal bipyramidal geometries as expected for a phosphorus(V) species with the *N*,*N*‐bis(3,5‐di‐*tert*‐butyl‐2‐phenolate)amide ligand occupying two axial and one equatorial positions. For **4 a** the P−O distances (1.688(1) and 1.681(1) Å) to the pincer ligand are similar to those observed for related distances in the H_2_O and NH_3_ activation products, whereas for **5 a** these distances are moderately longer (approx. 0.02 Å), presumably due to the greater σ‐donor ability of the benzyl functionality and greater degree of negative hyperconjugation.5

When **2 b** is reacted with an acid such as pyridinium trifluoromethanesulfonate the reaction affords HO*t*Bu and **1 b**, and not the arsenic(V) compound (*t*BuO)HAs(ONO). Similarly, no reaction between **2 b** and BzBr takes place even upon heating. These observations illustrate the increased stability of the +3 oxidation state on descending Group 15, and demonstrate that accessing arsenic(V) complexes bearing the ONO^3−^ ligand is synthetically very challenging.

### Reactivity of 1 a and 1 b towards electrophiles

The aforementioned results prompted us to explore the reactivity of **1 a** and **1 b** towards electrophilic substrates in an effort to establish whether they too associate with the heavier pnictogen atom centre, or whether they attack the more electronegative atoms of the ligand backbone. The highest occupied molecular orbital (HOMO) for the pyramidal *C_s_* isomer of **1 a** has a significant contribution from the nitrogen atomic orbitals, while the computed Hirshfeld charges reveal significant polarisation of the P−N and P−O bonds, with negative charge accumulating equally over the three atoms of the ligand backbone.

In a typical reaction, one molar equivalent of EOTf (E=H, Me; OTf=trifluoromethanesulfonate) was added to a solution of either **1 a** or **1 b**. The reactions were monitored by NMR spectroscopy and reveal quantitative conversion to [Pn{ON(H)O}][OTf] (Pn=P (**6 a**), As (**6 b**)) and [Pn{ON(Me)O}][OTf] (Pn=P (**7 a**), As (**7 b**)) after heating or sonicating the mixtures (see Experimental Section for full details).^31^ The ^31^P NMR spectra of the reactions involving **1 a** reveal broad resonances, with evidence of weak or non‐existent P−H coupling, at 155.5 (^2^ *J*_P‐H_=12 Hz) and 149.4 ppm for **6 a** and **7 a**, respectively (cf. 168.6 ppm for **1 a**), indicating that the electrophilic groups do not associate directly with the phosphorus(III) centre. The ^1^H NMR spectra of all four compounds are consistent with two equivalent aryloxide functionalities, which suggest functionalisation at the amide nitrogen atom. The ^1^H NMR resonances for the proton‐ and methyl‐group‐functionalised nitrogen atoms were observed at 14.27 and 3.72 ppm for **6 a** and **7 a**, respectively. Similarly, reactions involving **1 b** also reveal clean conversion to the products and the ^1^H NMR resonances of the electrophiles associated with the nitrogen atom were observed at 11.64 and 3.41 ppm for **6 b** and **7 b**, respectively.

**Figure 6:**
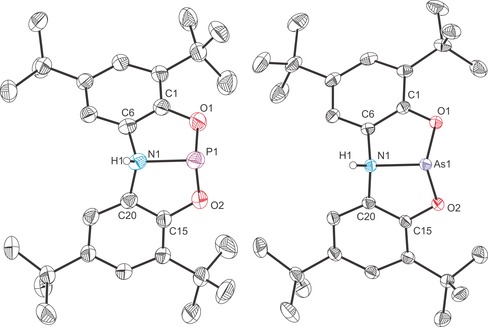
Molecular structures of [**6 a**][OTf]⋅HOTf (left) and [**6 b**][OTf] (right). Thermal ellipsoids pictured at 50 % probability level; HOTf/OTf^−^ and hydrogen atoms, with the exception of those bonded to N1, omitted for clarity. There are two crystallographically independent molecules of **6 b** in the lattice, for clarity, only bond metric data for one of them is provided. Selected interatomic distances [Å] and angles [°]; **6 a**: P1−N1 1.926(2), P1−O1 1.615(2), P1−O2 1.619(2), N1−C6 1.478(3), N1−C20 1.470(3), N1−H1 0.85(3), O1‐P1‐O2 104.80(10), N1‐P1‐O1 90.16(8), N1‐P1‐O2 104.80(10), C6‐N1‐C20 112.46(17), C6‐N1‐H1 114(2), C6‐N1‐P1 105.39(14), C20‐N1‐H1 111(2), C20‐N1‐P1 105.20(13), H1‐N1‐P1 108(2). **6 b**: As1−N1 2.028(2), As1−O1 1.791(1), As1−O2 1.810(1), N1−C6 1.472(2), N1−C20 1.466(2), N1−H1 0.82(2), O1‐As1‐O2 98.55(5), N1‐As1‐O1 87.00(5), N1‐As1‐O2 85.69(5), C6‐N1‐C20 114.51(11), C6‐N1‐H1 109.3(13), C6‐N1‐As1 105.16(9), C20‐N1‐H1 111.3(14), C20‐N1‐As1 105.87(8), H1‐N1‐As1 110.5(14).

**Figure 7:**
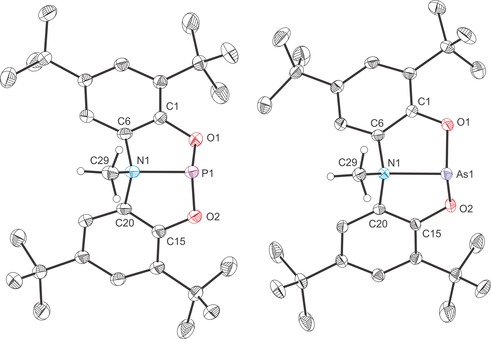
Molecular structures of [**7 a**][OTf] (left) and [**7 b**][OTf] (right). Thermal ellipsoids pictured at 50 % probability level; OTf^−^ and hydrogen atoms, with the exception of those bonded to C29, omitted for clarity. Selected interatomic distances [Å] and angles [°]; **7 a**: P1−N1 1.955(2), P1−O1 1.626(2), P1−O2 1.626(2), N1−C6 1.480(2), N1−C20 1.482(2), N1−C29 1.504(2), O1‐P1‐O2 104.00(7), N1‐P1‐O1 89.14(6), N1‐P1‐O2 90.38(6), C6‐N1‐C20 111.41(12), C6‐N1‐C29 113.51(12), C6‐N1‐P1 103.58(9), C20‐N1‐C29 111.78(12), C20‐N1‐P1 104.17(9), C29‐N1‐P1 111.71(10). **7 b**: As1−N1 2.127(1), As1−O1 1.790(1), As1−O2 1.788(1), N1−C6 1.469(2), N1−C20 1.468(2), N1−C29 1.496(2), O1‐As1‐O2 100.35(4), N1‐As1‐O1 85.00(4), N1‐As1‐O2 83.73(4), C6‐N1‐C20 112.64(9), C6‐N1‐C29 111.29(9), C6‐N1‐As1 104.58(7), C20‐N1‐C29 114.20(9), C20‐N1‐As1 103.43(7), C29‐N1‐As1 109.94(7).

The structures of all four novel cationic complexes were determined by single‐crystal X‐ray diffraction (Figures 6 and 7). The cationic moieties crystallise alongside a trifluoromethane sulfonate anion, and, in the case of **6 a** an additional molecule of HOTf. The most striking aspect of the structures is that protonation/methylation has taken place at the nitrogen centre, as evidenced by NMR spectroscopy. All four complexes adopt a distorted tetrahedral geometry around the nitrogen atom and exhibit a significant elongation of the N‐Pn bonds (N−P: 1.926(2) and 1.955(2) Å for **6 a** and **7 a**, respectively; N‐As: 2.028(2) and 2.127(1) Å for **6 b** and **7 b**, respectively) relative to **1 a** (1.757(1) Å) and **1 b** (1.862(3) Å). The Pn−O bonds experience a moderate contraction on protonation/methylation in the case of the phosphorus containing compounds (0.03 Å), but a much more dramatic contraction (0.13 Å) for the arsenic analogues, which is linked to a change from the planar geometry of **1 b** to the bent (*C_s_*) geometry of both **6 b** and **7 b**. This geometric change allows for the aryloxide substituents to get closer to the arsenic centre as the pseudo‐axial arrangement of the oxygen atoms in the 10‐Pn‐3 structure of **1 b** is lost on functionalisation of the ligand backbone.6, 7

Interestingly, all four species exhibit close contacts between the pnictogen(III) centre and trifluoromethanesulfonate anions (**6 a**: 2.790(2) and 2.898(1) Å, **6 b**: 2.745(2) Å, **7 a**: 2.760(2) **7 b**: 2.226(1) Å) indicating a significant degree of positive charge accumulating on the pnictogen centres on functionalisation of the ligand backbone. This is corroborated by DFT calculations which show a large Hirshfeld charges on the pnictogen atoms relative to nitrogen (**6 a**: 0.455 and −0.012, **6 b**: 0.598 and −0.024, **7 a**: 0.446 and 0.015, **7 b**: 0.586 and 0.004, for the heavier pnictogen and nitrogen atoms, respectively). Thus, all four complexes can be thought of as base‐stabilised phosphenium or arsenium ions. This bonding formulation has previously been proposed for related phosphorus‐containing compounds.^32^, ^33^, ^34^

It is interesting to note that while **1 a** reacts with HOTf to afford a phosphorus(III) compound with a protonated ligand backbone (**6 a**), the analogous reaction with an acid that has a more nucleophilic counter‐anion, such as HCl, affords the phosphorus(V) species HPCl(ONO) (**8 a**). This difference in reactivity suggests that mechanistically, a sufficiently strong nucleophile is required to afford the formal phosphorus(V) oxidative addition product. Reaction of **6 a** with KO*t*Bu, affords **4 a**, suggesting that nucleophilic association of the anionic ^−^O*t*Bu moiety with the phosphorus centre induces proton migration from the ligand backbone. Similarly, reaction of **6 a** with tetradodecylammonium chloride cleanly affords **8 a** and the corresponding ammonium trifluoromethane sulfonate salt. Conversely, reactions of **8 a** with one equivalent of trimethylsilyl trifluoromethane sulfonate show evidence for the formation of **6 a**, although full conversion was not observed at room temperature with such stoichiometric loadings.

In the aforementioned studies we have established the relative inaccessibility of the arsenic(V) oxidation state for **1 b**. This is borne out in the reactivity of **1 b** towards HCl. Whereas **1 a** reacts with HCl to afford **8 a**, the reaction of **1 b** with one molar equivalent of HCl results in the addition of the acid across one of the As−O bonds resulting in the arsenic(III) compound AsCl{(H)ONO} (**8 b**). This was evident from the ^1^H NMR spectra of these reaction mixtures which reveal the presence of two inequivalent arms of *N*,*N*‐bis(3,5‐di‐*tert*‐butyl‐2‐phenolate)amide pincer ligand on protonation.

**Figure 8:**
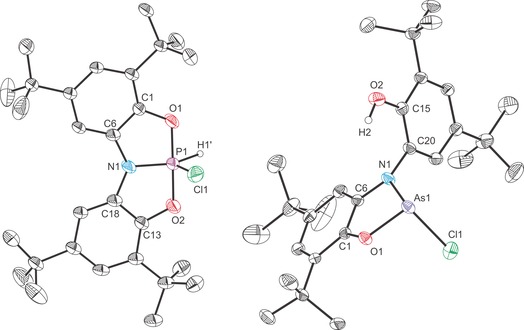
Molecular structures of **8 a** (left) and **8 b** (right). Thermal ellipsoids pictured at 50 % probability level; hydrogen atoms, with the exception of those bonded to P1 and O2, omitted for clarity. Selected interatomic distances [Å] and angles [°]; **8 a**: P1−N1 1.699(5), P1−O1 1.661(4), P1−O2 1.653(4), P1−Cl1 1.918(2), P1−H1 1.49(8), O1‐P1‐O2 179.3(2), O1‐P1‐Cl1 89.96(7), O1‐P1‐H1 87(3), O2‐P1‐Cl1 90.21(8), O2‐P1‐H1 94(3), Cl1‐P1‐H1 109(3), N1‐P1‐O1 89.6(2), N1‐P1‐O2 89.8(2), N1‐P1‐Cl1 105.26(7), N1‐P1‐H1 145(3), C6‐N1‐C18 131.4(5), C6‐N1‐P1 114.6(4), C18‐N1‐P1 114.0(4). **8 b**: As1−N1 1.839(2), As1−O1 1.783(2), As1−Cl1 2.286(1), O1‐As1‐N1 87.51(8), O1‐As1‐Cl1 95.66(5), N1‐As1‐Cl1 101.58(7), C6‐N1‐C20 120.09(18), C6‐N1‐As1 110.49(13), C20‐N1‐As1 124.99(16).

The structures of **8 a** and **8 b** were both determined by single‐crystal X‐ray diffraction (Figure 8). While **8 a** exhibits a distorted trigonal bipyramidal structure consistent with all of the phosphorus(V) complexes we have studied to date, the structure of **8 b** clearly shows a trigonal pyramidal arsenic(III) centre in which one of the *tert*‐butyl‐2‐phenolate ligand arms has been protonated and is consequently no longer coordinated to the arsenic centre. The bond metric data for **8 a** are similar to the other phosphorus(V) compounds reported. The P−O bond lengths to the *N*,*N*‐bis(3,5‐di‐*tert*‐butyl‐2‐phenolate)amide ligand, 1.661(4) and 1.653(4) Å, are slightly shorter than those observed for the aforementioned phosphorus(V) compounds (**4 a**: 1.688(1) and 1.681(1); **5 a**: 1.721(1) and 1.732(1) Å). The same is also true of the P−N interatomic distance which is 1.699(5) Å in **8 a**, and 1.720(1) and 1.717(1) Å in **4 a** and **5 a**, respectively. The P−Cl bond length is 1.918(2) Å.8

The structure of **8 b** reveals a trigonal pyramidal geometry about the arsenic(III) centre (Σ_angles(As)_=284.8°; Σ_angles(N)_=355.6°) with a relatively obtuse N1‐As1‐Cl1 angle due to the steric repulsion of the chloride with the free arm of the *N*,*N*‐bis(3,5‐di‐*tert*‐butyl‐2‐phenolate)amide ligand. The As−N and As−O distances, 1.839(2) and 1.783(2) Å, respectively, are notably shorter than those observed for **1 b** (As−N: 1.862(3) and As−O: 1.933(4) Å), presumably due to the relaxation of steric strain and the loss of As−N multiple bond character on bending the ligand backbone. It is worth noting that the As−N bond in **8 b** is still very short and indicative of some multiple bond character.

As mentioned previously, reactions of **6 a** with KO*t*Bu result in the association of the *tert*‐butoxide anion with the phosphorus(III) centre and migration of the ligand proton to afford the phosphorus(V) product **4 a** (which can also be accessed by a formal oxidative addition of HO*t*Bu to **1 a**). The facility with which the proton of the ligand backbone migrates prompted us to carry out related studies with the methylated species **7 a** and **7 b**. We hypothesised that methyl migration would not occur and that, consequently novel complexes in which both the nitrogen atom of the *N*,*N*‐bis(3,5‐di‐*tert*‐butyl‐2‐phenolate)amide ligand, and the heavier pnictogen centers could be functionalised.

**Scheme 3:**
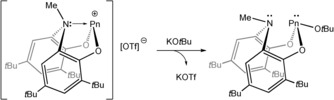
Formation of **9 a/9 b** from reaction of solutions of [**7 a/7 b**][OTf] with potassium *tert*‐butoxide.

In a typical reaction, **7 a** or **7 b** were treated with one molar equivalent of potassium *tert*‐butoxide. These reactions were found to quantitively afford novel complexes bearing a symmetrical ligand environment and an additional *tert*‐butoxide functionality (as pictured in Scheme 3). Reactions involving **7 a** reveal an upfield shift in resonance in the ^31^P NMR spectra from 149.4 to 142.8 ppm. The association of the ^−^O*t*Bu moiety with the phosphorus centre is evident from the appearance of a broad resonance in the ^1^H NMR spectrum at 1.57 ppm. This resonance is accompanied by two aromatic resonances (7.40 and 7.31 ppm), two resonances arising from the ligand *tert*‐butoxide groups and a doublet due to the methyl group of the ligand backbone. Comparable spectroscopic data were recorded for the arsenic‐containing analogue, **9 b**, although this sample could not be isolated as a compositionally pure compound due to its relative instability and tendency to decompose.3

**Figure 9:**
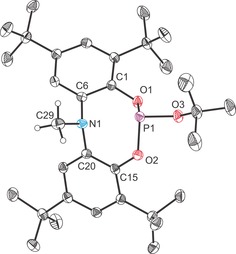
Molecular structure of **9 a** (thermal ellipsoids pictured at 50 % probability level; hydrogen atoms, with the exception of those bonded to C29, omitted for clarity). Selected interatomic distances [Å] and angles [°]: P1⋅⋅⋅N1 2.573(2), P1−O1 1.664(1), P1−O2 1.665(1), P1−O3 1.627(1), N1−C6 1.429(2), N1−C20 1.440(2), N1−C29 1.464(2), O1‐P1‐O2 99.56(5), N1‐P1‐O1 74.59(4), N1‐P1‐O2 77.16(4), O1‐P1‐O3 91.95(5), O2‐P1‐O3 93.65(5), C6‐N1‐C20 115.59(9), C6‐N1‐C29 115.94(10), C20‐N1‐C29 114.17(10).

Compound **9 a** was characterised by single‐crystal X‐ray crystallography (Figure 9) and reveals that the nucleophilic ^−^O*t*Bu moiety has associated with the phosphorus atom. Both the nitrogen and phosphorus centres exhibit distorted trigonal pyramidal geometries (Σ_angles(P)_=285.2°; Σ_angles(N)_=345.7°). That being said, the sum of bond angles around the nitrogen atom is strongly indicative of increased planarity, which we believe to arise due to the lone‐pair–lone‐pair repulsion arising between the nitrogen and phosphorus centres. The amine and phosphine like character of these centres is evident in the long P⋅⋅⋅N interatomic distance, 2.573(2) Å, which is notably longer than that observed for **6 a** and **7 a**, 1.926(2) and 1.955(2) Å, respectively, and is clearly indicative of the lack of a bonding interaction between the phosphorus and nitrogen centres. The P−O bonds range from 1.627(1) to 1.665(1) Å and are consistent with bond lengths reported for other phosphites.^35^ The N−C bond lengths to the aryl functionalities of the ligand backbone are comparable in magnitude (1.429(2) and 1.440(2) Å) and slightly shorter than that to the methyl functionality 1.464(2) Å, consistent with the reduced covalent radius of a formally sp^2^‐hybridised carbon atom compared to an sp^3^‐hybridised species Δ*r*=(0.03 Å).^29^, 9

## Conclusion

We have explored the reactivity of the geometrically constrained Group 15 complexes P(ONO) (**1 a**) and As(ONO) (**1 b**) towards ionic nucleophiles and electrophiles. These studies show that anionic nucleophiles readily associate with the pnictogen(III) centres in both complexes, suggesting that such an association may play an important role in the mechanism for the bond activation of NH_3_ and H_2_O by **1 a**. Our studies also reveal that while phosphorus(V) compounds are readily accessible by sequential reactions involving **1 a**, the corresponding arsenic(V) compounds cannot be synthesised from the heavier analogue **1 b**.

Reactions involving charged electrophilic substrates give rise to pnictogen(III) compounds in which the electrophile associates with the nitrogen atom of the ligand backbone. Interestingly, when the electrophile in question is a proton, it will associate with the nitrogen atom of the ligand backbone only in the presence of a weakly coordinating counteranion (such as OTf^−^). When a more nucleophilic counter‐anion is employed (such as Cl^−^) these reactions result in the generation of a phosphorus(V) compound by proton migration from the ligand backbone to the phosphorus centre. As with previous studies, the analogous arsenic(V) compound was found to be inaccessible.

These studies have helped us probe possible pathways by which **1 a** is able to activate nucleophilic substrates with polarised E−H bonds. Studies are currently on‐going with regard to elucidating a mechanism for such formal oxidative addition reactions.

## Experimental Section

**General procedures**: All reactions and product manipulations were carried out under an inert atmosphere of argon or dinitrogen using standard Schlenk‐line or glovebox techniques (MBraun UNIlab glovebox maintained at <0.1 ppm H_2_O and <0.1 ppm O_2_). ^1^H, ^13^C, ^31^P, ^19^F NMR spectra were recorded at room temperature using a Bruker AVIII 500 MHz or Bruker AVIII HD NanoBay 400 MHz NMR Spectrometer. ^1^H and ^13^C{^1^H} spectra are reported relative to tetramethylsilane (TMS) and were referenced to the most downfield residual solvent resonance (^1^H NMR spectroscopy: 7.26 ppm (CDCl_3_), 7.16 ppm (C_6_D_6_), 5.32 pm (CD_2_Cl_2_) and 3.58 ppm ([D_8_]THF); ^13^C{^1^H} NMR spectroscopy: 77.16 ppm (CDCl_3_), 128.06 ppm (C_6_D_6_), 53.84 ppm (CD_2_Cl_2_) and 67.21 ppm ([D_8_]THF)). ^31^P NMR chemical shifts were externally referenced to an 85 % solution of H_3_PO_4_ (aq). ^19^F NMR chemical shifts were externally referenced to CF_3_COOH. Elemental analyses were performed by Elemental analysis Ltd (Devon). EI/CI mass spectra were obtained on neat samples using a Waters GCT Time of Flight Mass Spectrometer with a temperature programmed solids probe inlet, or an Agilent 7200 Accurate‐Mass Q‐TOF GCMS with a SIM Direct Inlet probe. ESI mass spectra were obtained from DMF solutions using a Waters LCT time‐of‐flight mass spectrometer with a Z‐spray source (150 °C source temperature, 200 °C desolvation temperature, 2.4 kV capillary voltage and 25 V cone voltage).

**Solvents and reagents**: Hexane (Sigma–Aldrich, ≥97.0 %), pentane (Sigma–Aldrich, ≥99.0 %) and toluene (Sigma–Aldrich, 99.9 %) were dried using an MBraun SPS‐800 solvent system, THF (Sigma–Aldrich, ≥99.9 %) was dried over a potassium metal/benzophenone mixture and pyridine was distilled from CaH_2_. C_6_D_6_ (Aldrich, 99.6 %) was dried and stored over activated 3 Å molecular sieves. CDCl_3_ (Aldrich, 99.8 %), CD_2_Cl_2_ (Euriso‐top, 99.90 %) and [D_8_]THF (Euriso‐top, 99.50 %) were each dried over CaH_2_ and stored over activated 3 Å molecular sieves. H_3_ONO and KNPh_2_ were synthesised according to a previously reported synthetic procedure.^36^, ^37^ 1,4,7,10,13,16‐Hexaoxacyclooctadecane (18‐crown‐6, Alfa Aesar, 99 %) was purified by sublimation and NEt_3_ (Sigma–Aldrich, 99.5 %) was distilled from CaH_2_ prior to use. AsCl_3_ (Sigma–Aldrich, 99.99 %), KO*t*Bu (Aldrich, ≥98 %), 4,7,13,16,21,24‐hexaoxa‐1,10‐diazabicyclo[8,8,8]‐hexacosane (2,2,2‐crypt, VWR, 99 %), [PyH][OTf] (Alfa Aesar, 98 %), HOTf (Sigma–Aldrich, 98 %), MeOTf (Apollo, 98 %), benzyl bromide (Aldrich, 98 %) and HCl (Alfa Aesar, 2 m in Et_2_O) were used as received.

**Synthesis of As(ONO) (1 b)**: H_3_ONO (1.00 g, 2.35 mmol) was dissolved in toluene (10 mL). AsCl_3_ (198 μL, 2.35 mmol) was added to the stirred solution followed by NEt_3_ (1.00 mL, 7.17 mmol), yielding a dark orange solution and white precipitate. The mixture was stirred at room temperature for 3 h. All volatiles were removed under a dynamic vacuum and the product was extracted in pentane (3×10 mL). Removal of volatiles in vacuo yielded **1 b** as a dark orange solid. Crystals suitable for single‐crystal X‐ray diffraction analysis were grown from a concentrated pentane solution at −30 °C. Yield: 880 mg (75 %); elemental analysis calcd (%) for C_28_H_40_AsNO_2_: C 67.59, H 8.10, N 2.82; found: C 67.15, H 8.09, N 2.93; EI MS: *m*/*z* calcd for C_28_H_40_AsNO_2_: 497.2275; found: 497.2272; *m*/*z* calcd for C_27_H_37_AsNO_2_: 482.2040; found: 482.1898. ^1^H NMR (499.93 MHz, C_6_D_6_, 298 K): *δ*=8.39 (s, 2 H; Ar), 7.51 (s, 2 H; Ar), 1.71 (s, 18 H; *t*Bu), 1.46 ppm (s, 18 H; *t*Bu). ^13^C{^1^H} NMR (125.72 MHz, C_6_D_6_, 298 K): *δ*=154.0 (Ar), 142.3 (Ar), 137.2 (Ar), 135.9 (Ar), 119.9 (Ar), 111.3 (Ar), 35.7 (*C*(CH_3_)_3_), 35.1 (*C*(CH_3_)_3_), 32.0 (C(*C*H_3_)_3_), 30.0 ppm (C(*C*H_3_)_3_).

**Synthesis of [K(18‐crown‐6)][(*t*BuO)P(ONO)] ([K(18‐crown‐6)][2 a])**: Compound **1 a** (300 mg, 0.661 mmol), KO*t*Bu (74.2 mg, 0.661 mmol) and 18‐crown‐6 (175 mg, 0.663 mmol) were dissolved in THF (10 mL) at room temperature to give a pale yellow solution. After stirring for 30 min, the solution was filtered through a cannula and all volatiles were removed in vacuo. The residue was washed with toluene (3×5 mL) and the product was dried under a dynamic vacuum. Crystals suitable for X‐ray diffraction studies were grown by slow diffusion of hexane into a THF solution of the product at room temperature. Yield: 296 mg (54 %); elemental analysis calcd (%) for C_44_H_73_KNO_9_P⋅0.5 (C_4_H_8_O): C 63.79, H 8.96, N 1.62; found: C 64.06, H 9.34, N 1.64. ESI MS (−ve mode, DMF): *m*/*z* calcd for C_32_H_49_NO_3_P: 526.35; found: 527.01. ^1^H NMR (400.17 MHz, [D_8_]THF, 298 K): *δ*=7.39 (d, ^4^ *J*_H‐H_=2 Hz, 2 H; Ar), 6.57 (d, ^4^ *J*_H‐H_=2 Hz, 2 H; Ar), 3.40 (s, 24 H; 18‐crown‐6), 1.43 (s, 18 H; *t*Bu), 1.34 (s, 9 H; *t*BuO), 1.32 ppm (s, 18 H; *t*Bu); ^13^C{^1^H} NMR (100.62 MHz, [D_8_]THF, 298 K): *δ*=151.8 (d, ^2^ *J*_C‐P_=5 Hz; Ar), 134.5 (d, ^2^ *J*_C‐P_=4 Hz; Ar), 134.0 (Ar), 129.4 (d, ^3^ *J*_C‐P_=5 Hz; Ar), 112.1 (Ar), 107.0 (d, ^3^ *J*_C‐P_=3 Hz; Ar), 72.1 (d, ^2^ *J*_C‐P_=11 Hz; O*C*(CH_3_)_3_), 70.8 (18‐crown‐6), 35.0 (*C*(CH_3_)_3_), 34.9 (*C*(CH_3_)_3_), 32.7 (C(*C*H_3_)_3_), 31.9 (d, ^3^ *J*_C‐P_=12 Hz; OC(*C*H_3_)_3_), 30.4 ppm (C(*C*H_3_)_3_); ^31^P NMR (161.99 MHz, [D_8_]THF, 298 K): *δ* 85.5 ppm (s); ^31^P{^1^H} NMR (161.99 MHz, [D_8_]THF, 298 K): *δ* 85.5 ppm (s).

**Synthesis of [K(18‐crown‐6)][(*t*BuO)As(ONO)] ([K(18‐crown‐6)][2 b])**: Compound **1 b** (234 mg, 0.470 mmol), KO*t*Bu (52.7 mg, 0.470 mmol) and 18‐crown‐6 (124 mg, 0.469 mmol) were dissolved in THF (10 mL) at room temperature. The mixture was stirred at room temperature for 30 min after which the solvent was removed under vacuum. The product was crystallised as white solid from hexane. The solution was filtered and the white solid dried in vacuo. Crystals suitable for X‐ray diffraction studies were grown by slow diffusion of hexane into a THF solution of the product at room temperature. Yield: 355 mg (86 %); elemental analysis calcd (%) for C_44_H_73_AsKNO_9_: C 60.46, H 8.42, N 1.60; found: C 60.52, H 8.54, N 1.44. ESI MS (−ve mode, DMF): *m*/*z* calcd for C_32_H_49_AsNO_3_ [*M*]^−^: 570.29; found: 569.64; *m*/*z* calcd C_28_H_40_AsNO_3_ 513.22; found: 513.61; ^1^H NMR (400.17 MHz, [D_8_]THF, 298 K): *δ*=7.54 (d, ^4^ *J*_H‐H_=2 Hz, 2 H; Ar), 6.56 (d, ^4^ *J*_H‐H_=2 Hz, 2 H; Ar), 3.38 (s, 24 H; 18‐crown‐6), 1.44 (s, 18 H; *t*Bu), 1.33 (s, 18 H; *t*Bu), 1.26 ppm (s, 9 H; O*t*Bu). ^13^C{^1^H} NMR (100.62 MHz, [D_8_]THF, 298 K): *δ*=152.6 (Ar), 135.8 (Ar), 133.5 (Ar), 130.4 (Ar), 111.6 (Ar), 107.4 (Ar), 71.2 (O*C*(CH_3_)_3_), 70.8 (18‐crown‐6), 35.3 (*C*(CH_3_)_3_), 34.9 (*C*(CH_3_)_3_), 32.8 (C(*C*H_3_)_3_), 32.8 (OC(*C*H_3_)_3_), 30.4 ppm (s, C(*C*H_3_)_3_).

**Synthesis of [K(18‐crown‐6)][(Ph_2_N)P(ONO)] ([K(18‐crown‐6)][3 a])**: A preparative synthesis of [K(18‐crown‐6)][**3 a**] was not possible due to extremely high sensitivity of the product, which upon manipulation readily decomposes to [(Ph_2_N)HP(ONO)]. However, evidence for the formation of **3 a** can be observed by NMR tube scale reactions. In a typical reaction, **1 a** (20.0 mg, 0.0440 mmol), KNPh_2_⋅THF_0.15_ (9.6 mg, 0.0441 mmol) and 18‐crown‐6 (11.6 mg, 0.0439 mmol) were added to an NMR tube equipped with a J. Young airtight tap and the mixture dissolved in [D_8_]THF to give a pale yellow solution. ^1^H NMR (400.20 MHz, [D_8_]THF, 298 K): *δ*=7.07 (d, ^4^ *J*_H‐H_=2 Hz, 2 H; Ar), 6.90–6.85 (m, 4 H; NPh_2_), 6.79–6.73 (m, 4 H; NPh_2_), 6.52–6.47 (m, 2 H; NPh_2_), 6.51 (d, ^4^ *J*_H‐H_=2 Hz, 2 H; Ar), 3.49 (s, 24 H; 18‐crown‐6), 1.28 (s, 18 H; *t*Bu), 1.26 ppm (s, 18 H; *t*Bu); ^13^C{^1^H} NMR (100.63 MHz, [D_8_]THF, 298 K): *δ*=151.2 (d, ^2^ *J*_C‐P_=5 Hz; Ar), 149.4 (d, ^2^ *J*_C‐P_=8 Hz; NPh_2_), 135.0 (Ar), 134.8 (d, ^2^ *J*_C‐P_=3 Hz; Ar), 129.8 (d, ^3^ *J*_C‐P_=5 Hz; Ar), 127.6 (s; NPh_2_), 125.5 (br s; NPh_2_), 120.0 (br s; NPh_2_), 112.4 (Ar), 106.7 (d, ^3^ *J*_C‐P_=4 Hz; Ar), 70.8 (s; 18‐crown‐6), 34.9 (*C*(CH_3_)_3_), 24.8 (*C*(CH_3_)_3_), 32.5 (C(*C*H_3_)_3_), 30.2 ppm (C(*C*H_3_)_3_); ^31^P NMR (162.00 MHz, [D_8_]THF, 298 K): *δ*=66.3 ppm (s); ^31^P{^1^H} NMR (162.00 MHz, [D_8_]THF, 298 K): *δ*=66.3 ppm (s).

**Synthesis of [K(2,2,2‐crypt)][(Ph_2_N)As(ONO)] ([K(18‐crown‐6)][3 b])**: Compound **1 b** (160 mg, 0.322 mmol), KNPh_2_⋅THF_0.15_ (70.1 mg, 0.322 mmol) and 2,2,2‐crypt (121 mg, 0.322 mmol) were dissolved in THF (10 mL) resulting in a yellow solution. The mixture was stirred at room temperature for 15 min after which the solution was concentrated to 0.5 mL in vacuo. Crystals of the product were obtained by slow diffusion of hexane into the THF solution and were isolated by filtration. Yield: 245 mg (71 %); elemental analysis calcd (%) for C_58_H_86_AsKN_4_O_8_: C 64.42, H 8.02, N 5.18; found: C 63.48, H 7.84, N 5.23. ESI MS (−ve mode, DMF): *m*/*z* calcd for C_28_H_42_AsN_2_O_2_ ([(H_2_N)As(ONO)]^−^) 513.24; found: 513.97; ^1^H NMR (400.17 MHz, [D_8_]THF, 298 K): *δ*=7.17 (d, ^4^ *J*_H‐H_=2 Hz, 2 H; Ar), 6.76–6.70 (m, 8 H; NPh_2_), 6.50 (d, ^4^ *J*_H‐H_=2 Hz, 2 H; Ar), 6.46–6.41 (m, 2 H; NPh_2_), 3.46 (s, 12 H; 2,2,2‐crypt), 3.43–3.41 (m, 12 H; 2,2,2‐crypt), 2.46–2.43 (m, 12 H; 2,2,2‐crypt), 1.36 (s, 18 H; *t*Bu), 1.27 ppm (s, 18 H; *t*Bu); ^13^C{^1^H} NMR (100.62 MHz, [D_8_]THF, 298 K): *δ*=152.2 (Ar), 150.5 (NPh_2_), 135.8 (Ar), 134.1 (Ar), 130.6 (Ar), 127.9 (NPh_2_), 124.5 (br; NPh_2_), 119.4 (br; NPh_2_), 111.8 (Ar), 107.5 (Ar), 71.0 (2,2,2‐crypt), 68.2 (2,2,2‐crypt), 54.5 (2,2,2‐crypt), 35.2 (*C*(CH_3_)_3_), 34.8 (*C*(CH_3_)_3_), 32.6 (C(*C*H_3_)_3_), 30.3 ppm (C(*C*H_3_)_3_).

**Synthesis of (*t*BuO)HP(ONO) (4 a)**: Compound **1 a** (150 mg, 0.331 mmol) and KO*t*Bu (37.1 mg, 0.331 mol) were dissolved in THF (3 mL) at room temperature. The yellow solution was stirred for 15 min after which a solution of pyridinium triflate (75.8 mg, 0.331 mmol) in THF (2 mL) was added, giving rise to a colourless solution and white precipitate. After stirring for 30 min all volatiles were removed in vacuo and the product extracted into toluene (5 mL). The solution was filtered through a cannula, concentrated to approximately 0.5 mL and then cooled to 5 °C for a week, yielding large colourless block crystals of **4 a**. The product was isolated by filtration. Yield: 145 mg (83 %); elemental analysis calcd (%) for C_32_H_50_NO_3_P: C 72.83, H 9.55, N 2.65; found: C 72.92, H 9.62, N 2.75. EI MS: *m*/*z* calcd for C_32_H_50_NO_3_P: 528.3607; found: 528.3600 *m*/*z* calcd for C_28_H_43_NO_3_P: 472.2981; found: 472.2968; *m*/*z* calcd for C_28_H_41_NO_2_P: 454.2875; found: 454.2852; ^1^H NMR (400.20 MHz, C_6_D_6_, 298 K): *δ*=8.16 (d, ^1^ *J*_H‐P_=856 Hz, 1 H; P*H*), 7.80 (s, 2 H; Ar), 7.22 (d, ^4^ *J*_H‐H_=2 Hz, 2 H; Ar), 1.64 (s, 18 H; *t*Bu), 1.42 (s, 18 H; *t*Bu), 1.19 ppm (s, 9 H; O*t*Bu); ^1^H{^31^P} NMR (400.20 MHz, C_6_D_6_, 298 K): *δ*=8.16 ppm (s, 1 H; PH), all other resonances the same as above; ^13^C{^1^H} NMR (100.63 MHz, C_6_D_6_, 298 K): *δ*=143.0 (Ar), 141.0 (d, ^2^ *J*_C‐P_=7 Hz; Ar), 132.7 (d, ^3^ *J*_C‐P_=6 Hz; Ar), 129.5 (d, ^2^ *J*_C‐P_=24 Hz; Ar), 115.4 (Ar), 106.7 (d, ^3^ *J*_C‐P_=16 Hz; Ar), 80.0 (d, ^2^ *J*_C‐P_=8 Hz; O*C*(CH_3_)_3_), 35.0 (*C*(CH_3_)_3_), 34.8 (C(*C*H_3_)_3_), 32.0 (C(*C*H_3_)_3_), 30.3 (d, ^3^ *J*_C‐P_=6 Hz; OC(*C*H_3_)_3_), 30.2 (C(*C*H_3_)_3_). ^31^P NMR (161.99 MHz, C_6_D_6_, 298 K): *δ*=−38.7 ppm (d, ^1^ *J*_P‐H_=856 Hz); ^31^P{^1^H} NMR (161.99 MHz, C_6_D_6_, 298 K): *δ*=−38.7 ppm (s).

**Synthesis of (*t*BuO)BzP(ONO) (5 a)**: Compound **1 a** (100 mg, 0.220 mmol) and KO*t*Bu (24.7 mg, 0.220 mmol) were dissolved in THF (5 mL) and stirred until all solid material had dissolved. Benzyl bromide (26.2 μL, 0.220 mmol) was added, resulting in the formation of a white precipitate of KBr. The slurry was stirred for 30 min and then all volatiles were removed in vacuo. The product was extracted into hexane and filtered through a cannula. The solution was concentrated under a dynamic vacuum and cooled to −30 °C, yielding a crop of colourless crystals which were isolated by filtration. Yield: 101 mg (74 %); elemental analysis calcd (%) for C_39_H_56_NO_3_P: C 75.82, H 9.14, N 2.27; found: C 76.32, H 9.25, N 2.34; CI MS (NH_3_ carrier gas): *m*/*z* calcd C_35_H_45_NO_2_P⋅NH_4_: 562.3688; found: 562.3479; ^1^H NMR (400.17 MHz, C_6_D_6_, 298 K): *δ*=7.88 (d, ^4^ *J*_H‐H_=2 Hz, 2 H; Ar), 7.32–7.27 (m, 2 H; CH_2_(C_6_ *H*_5_)), 7.22 (d, ^4^ *J*_H‐H_=2 Hz, 2 H; Ar), 6.96–6.90 (m, 2 H; CH_2_(C_6_ *H*_5_)), 6.85–6.79 (m, 1 H; CH_2_(C_6_ *H*_5_)), 3.78 (d, ^2^ *J*_H‐P_=22 Hz, 2 H; C*H*_2_(C_6_H_5_)), 1.61 (s, 18 H; *t*Bu), 1.38 (s, 18 H; *t*Bu), 1.15 ppm (s, 9 H; O*t*Bu); ^1^H{^31^P} NMR (499.93 MHz, C_6_D_6_, 298 K): 3.78 ppm (s; C*H*_2_(C_6_H_5_)) all other resonances as above; ^13^C{^1^H} NMR (100.62 MHz, CD_2_Cl_2_, 298 K): *δ*=142.9 (d, ^2^ *J*_C‐P_=5 Hz; Ar), 141.7 (Ar), 134.8 (d, ^2^ *J*_C‐P_=12 Hz; CH_2_(*C*_6_H_5_)), 131.8 (d, ^3^ *J*_C‐P_=5 Hz; Ar), 130.0 (d, ^4^ *J*_C‐P_=9 Hz; CH_2_(*C*_6_H_5_)), 128.8 (d, ^2^ *J*_C‐P_=20 Hz; Ar), 128.5 (d, ^3^ *J*_C‐P_=5 Hz; CH_2_(*C*_6_H_5_)), 126.5 (d, ^5^ *J*_C‐P_=5 Hz; CH_2_(*C*_6_H_5_)), 116.1 (Ar), 106.7 (d, ^3^ *J*_C‐P_=13 Hz; Ar), 80.6 (d, ^2^ *J*_C‐P_=14 Hz; O*C*(CH_3_)_3_), 47.2 (d, ^1^ *J*_C‐P_=188 Hz, *C*H_2_Ph), 35.0 (*C*(CH_3_)_3_), 34.7 (*C*(CH_3_)_3_), 32.0 (C(*C*H_3_)_3_), 30.4 (d, ^3^ *J*_C‐P_=5 Hz; OC(*C*H_3_)_3_), 30.3 ppm (C(*C*H_3_)_3_); ^31^P NMR (161.99 MHz, C_6_D_6_, 298 K): *δ*=−20.2 ppm (t, ^2^ *J*_P‐H_=22 Hz); ^31^P{^1^H} NMR (161.99 MHz, C_6_D_6_, 298 K): *δ*=−20.2 ppm (s).

**Synthesis of [P{ON(H)O}][OTf] ([6 a]OTf)**: Compound **1 a** (360 mg 0.794 mmol) was suspended in hexane (5 mL) and HOTf (70.2 μL, 0.794 mmol) was added, affording a colourless solution. The reaction mixture was sonicated for 15 min causing precipitation of a white solid. The solid was isolated by filtration. Yield: 325 mg (68 %); elemental analysis calcd (%) for C_29_H_41_F_3_NO_5_PS⋅HCF_3_O_3_S: C 47.81, H 5.62, N 1.86; found: C 47.78, H 5.66, N 2.02. CI MS (NH_3_ carrier gas): *m*/*z* calcd for C_28_H_41_NO_2_P⋅NH_4_: 472.3219; found: 472.3002; ^1^H NMR (400.17 MHz, CD_2_Cl_2_, 298 K): *δ*=14.27 (br s, 1 H; N*H*), 7.88 (s, 2 H; Ar), 7.41 (s, 2 H; Ar), 1.38 (s, 18 H; *t*Bu), 1.33 ppm (s, 18 H; *t*Bu); ^13^C{^1^H} NMR (100.62 MHz, CD_2_Cl_2_, 298 K): *δ*=149.9 (Ar), 147.0 (d, ^2^ *J*_C‐P_=12 Hz; Ar), 138.2 (Ar), 128.9 (Ar), 126.3 (Ar), 116.5 (Ar), 35.6 (*C*(CH_3_)_3_), 35.4 (*C*(CH_3_)_3_), 31.4 (C(*C*H_3_)_3_), 29.4 ppm (C(*C*H_3_)_3_); ^31^P NMR (161.99 MHz, CD_2_Cl_2_, 298 K): *δ*=155.5 ppm (br d, ^2^ *J*_C‐P_=12 Hz); ^31^P{^1^H} NMR (161.99 MHz, CD_2_Cl_2_, 298 K): *δ*=155.5 ppm (br s, W_1/2_=57 Hz); ^19^F NMR (376.54 MHz, CD_2_Cl_2_, 298 K): *δ*=−78.6 ppm.

**Synthesis of [As{ON(H)O}][OTf] ([6 b]OTf)**: Compound **1 b** (108 mg, 0.217 mmol) was dissolved in hexane (5 mL) and HOTf (19.2 μL, 0.217 mmol) was added, resulting in a pale pink solution and the formation of a white precipitate. The mixture was stirred at room temperature for 15 min after which the solid was isolated by filtration. Yield: 64 mg (46 %); elemental analysis calcd (%) for C_28_H_41_AsF_3_NO_5_S: C 53.78, H 6.38, N 2.16; found: C 54.33, H 6.80, N 2.21. CI MS (CH_4_ carrier gas): *m*/*z* calcd for C_28_H_41_AsNO_2_: 498.2353; found: 498.2426; *m*/*z* calcd C_27_H_37_AsNO_2_: 482.2027; found: 482.2133; ^1^H NMR (400.17 MHz, C_6_D_6_, 298 K): *δ*=11.64 (br, 1 H; N*H*), 7.84 (d, ^4^ *J*_H‐H_=2 Hz, 2 H; Ar), 7.25 (d, ^4^ *J*_H‐H_=2 Hz, 2 H; Ar), 1.36 (s, 18 H; *t*Bu), 1.23 ppm (s, 18 H; *t*Bu). ^13^C{^1^H} NMR (100.62 MHz, C_6_D_6_, 298 K): *δ*=151.8 (Ar), 145.5 (Ar), 137.9 (Ar), 131.3 (Ar), 124.5 (Ar), 117.7 (Ar), 35.2 (*C*(CH_3_)_3_), 34.9 (*C*(CH_3_)_3_), 31.4 (C(*C*H_3_)_3_), 29.3 ppm (C(*C*H_3_)_3_); ^19^F{^1^H} NMR (376.54 MHz, C_6_D_6_, 298 K): *δ*=−78.3 ppm.

**Synthesis of [P{ON(Me)O}][OTf] ([7 a]OTf)**: Compound **1 a** (100 mg, 0.220 mmol) was dissolved in toluene (5 mL) and MeOTf (24.9 μL, 0.220 mmol) was added. The solution was heated at 70 °C for 24 h resulting in the growth of colourless needle‐shaped crystals. The supernatant was removed by cannula filtration and the crystals washed with toluene (3×5 mL) and dried in vacuo. Yield: 85 mg (60 %); elemental analysis calcd (%) for C_30_H_43_F_3_NO_5_PS: C 58.33, H 7.02, N 2.27; found: C 58.50, H 7.10, N 2.35; CI MS (NH_3_ carrier gas): *m*/*z* calcd for C_29_H_43_NO_2_P⋅NH_4_: 486.3375; found: 486.3151; ^1^H NMR (499.93 MHz, CD_2_Cl_2_, 298 K): *δ*=7.60 (br s, 2 H; Ar), 7.39 (d, ^4^ *J*_H‐H_=2 Hz, 2 H; Ar), 3.72 (d, ^3^ *J*_H‐P_=7 Hz, 3 H; NC*H*_3_), 1.39 (s, 18 H; *t*Bu), 1.33 ppm (s, 18 H; *t*Bu); ^1^H{^31^P} NMR (499.93 MHz, CD_2_Cl_2_, 298 K): *δ*=3.72 ppm (s, 3 H; NCH_3_) all other resonances as above; ^13^C{^1^H} NMR (125.71 MHz, CD_2_Cl_2_, 298 K): *δ*=149.3 (Ar), 147.0 (d, ^2^ *J*_C‐P_=12 Hz; Ar), 139.2 (Ar), 134.0 (d, ^2^ *J*_C‐P_=3 Hz; Ar), 126.0 (Ar), 114.8 (Ar), 47.6 (d, ^2^ *J*_C‐P_=26 Hz, N*C*H_3_), 35.6 (*C*(CH_3_)_3_), 35.5 (*C*(CH_3_)_3_), 31.5 (C(*C*H_3_)_3_), 29.4 ppm (C(*C*H_3_)_3_); ^31^P NMR (161.99 MHz, CD_2_Cl_2_, 298 K): *δ*=149.4 ppm (br s); ^31^P{^1^H} NMR (161.99 MHz, CD_2_Cl_2_, 298 K): *δ*=149.4 ppm (br s); ^19^F NMR (376.54 MHz, CD_2_Cl_2_, 298 K): *δ* −78.7 ppm.

**Synthesis of [As{ON(Me)O}][OTf] ([7 b]OTf)**: Compound **1 b** (705 mg, 1.417 mmol) was dissolved in toluene (15 mL) and MeOTf (160 μL, 1.457 mmol) was added to the solution, which was stirred at 80 °C for three days, resulting in the precipitation of a white/pink solid. The solid was isolated by filtration and dried in vacuo. Yield: 733 mg (78 %); elemental analysis calcd (%) for C_30_H_43_AsF_3_NO_5_S: C 54.46, H 6.55, N 2.12; found C 54.40, H 6.48, N 2.28; EI MS: *m*/*z* calcd for C_29_H_43_AsNO_2_⋅CF_3_O_3_S: 661.2030; found: 661.2022; *m*/*z* calcd for C_29_H_43_AsNO_2_: 512.2528; found: 512.2510; ^1^H NMR (400.17 MHz, CDCl_3_, 298 K): *δ*=7.31 (d, ^4^ *J*_H‐H_=2 Hz, 2 H; Ar), 7.28 (d, ^4^ *J*_H‐H_=2 Hz, 2 H; Ar), 3.41 (s, 3 H; NC*H*_3_), 1.40 (s, 18 H; *t*Bu), 1.31 ppm (s, 18 H; *t*Bu); ^13^C{^1^H} NMR (100.62 MHz, CDCl_3_, 298 K): *δ*=149.4 (Ar), 145.6 (Ar), 139.7 (Ar), 135.0 (Ar), 124.5 (Ar), 114.8 (Ar), 46.2 (N*C*H_3_), 35.5 (*C*(CH_3_)_3_), 34.9 (*C*(CH_3_)_3_), 31.6 (C(*C*H_3_)_3_), 29.4 ppm (C(*C*H_3_)_3_); ^19^F NMR (376.54 MHz, CDCl_3_, 298 K): *δ*=−77.9 ppm (br s).

**Synthesis of HPCl(ONO) (8 a)**: Compound **1 a** (200 mg, 0.441 mmol) was dissolved in Et_2_O (5 mL) and HCl (220 µL, 2 m in Et_2_O, 0.440 mmol) was added. The colourless solution was stirred for 30 min after which all volatiles were removed in vacuo. The colourless solid was extracted into hot hexane (3 mL) and the solution filtered. Colourless crystals of **8 a** grew upon standing at room temperature, which were isolated by filtration. A further crop could be isolated by concentrating the mother liquor. Yield: 139 mg (64 %); elemental analysis calcd (%) for C_28_H_41_ClNO_2_P: C 68.63, H 8.43, N 2.86; found: C 68.86, H 8.49, N 2.92; ^1^H NMR (400.20 MHz, CD_2_Cl_2_, 298 K): *δ*=8.97 (d, ^1^ *J*_H‐P_=967 Hz, 1 H; P*H*), 7.66 (s, 2 H; Ar), 7.13 (d, ^4^ *J*_H‐H_=2 Hz, 2 H; Ar), 1.45 (s, 18 H; *t*Bu), 1.41 ppm (s, 18 H; *t*Bu); ^1^H{^31^P} NMR (400.20 MHz, CD_2_Cl_2_, 298 K): *δ*=8.97 ppm (s, 1 H; PH), all other resonances as above; ^13^C{^1^H} NMR (100.63 MHz, CD_2_Cl_2_, 298 K): *δ*=144.2 (Ar), 139.4 (d, ^2^ *J*_C‐P_=9 Hz; Ar), 133.9 (d, ^3^ *J*_C‐P_=6 Hz; Ar), 126.4 (d, ^2^ *J*_C‐P_=24 Hz; Ar), 116.6 (Ar), 106.4 (d, ^3^ *J*_C‐P_=17 Hz; Ar), 34.6 (*C*(CH_3_)_3_), 34.1 (*C*(CH_3_)_3_), 31.1 (C(*C*H_3_)_3_), 29.0 ppm (C(*C*H_3_)_3_); ^31^P NMR (261.99 MHz, CD_2_Cl_2_, 298 K): *δ*=−31.3 ppm (d, ^1^ *J*_P‐H_=967 Hz); ^31^P{^1^H} NMR (161.99 MHz, CD_2_Cl_2_, 298 K): *δ*=−31.3 ppm (s).

**Synthesis of AsCl(HONO) (8 b)**: Compound **1 b** (210 mg, 0.422 mmol) was dissolved in THF (5 mL). HCl (211 μL, 2 m in Et_2_O, 0.422 mmol) was added turning the solution pale yellow. The mixture was stirred at room temperature for 15 min and then concentrated in vacuo to 0.5 mL promoting the growth of colourless block crystals of the product. These were isolated by filtration. Yield: 155 mg (69 %); elemental analysis calcd (%) for C_28_H_41_AsClNO_2_: C 62.98, H 7.74, N 2.62; found: C 62.64, H 7.74, N 2.96; ^1^H NMR (400.17 MHz, C_6_D_6_, 298 K): *δ*=7.53 (d, ^4^ *J*_H‐H_=2 Hz, 1 H; Ar), 7.41 (d, ^4^ *J*_H‐H_=2 Hz, 1 H; Ar), 7.19 (d, ^4^ *J*_H‐H_=2 Hz, 1 H; Ar), 6.69 (d, ^4^ *J*_H‐H_=2 Hz, 1 H; Ar), 5.48 (s, 1 H; ArOH), 1.63 (s, 9 H; *t*Bu), 1.57 (s, 9 H; *t*Bu), 1.27 (s, 9 H; *t*Bu), 1.16 ppm (s, 9 H; *t*Bu); ^13^C{^1^H} NMR (100.62 MHz, C_6_D_6_, 298 K): *δ*=149.7 (Ar), 147.9 (Ar), 146.1 (Ar), 143.5 (Ar), 137.4 (Ar), 137.2 (Ar), 136.7 (Ar), 124.6 (Ar), 124.0 (Ar), 123.6 (Ar), 116.5 (Ar), 108.5 (Ar), 35.6 (*C*(CH_3_)_3_), 35.1 (*C*(CH_3_)_3_), 34.8 (*C*(CH_3_)_3_), 34.6 (*C*(CH_3_)_3_), 31.7 (C(*C*H_3_)_3_), 31.6 (C(*C*H_3_)_3_), 30.0 (C(*C*H_3_)_3_), 29.8 ppm (C(*C*H_3_)_3_).

**Synthesis of (*t*BuO)P{ON(Me)O} (9 a)**: Compound **7 a** (120 mg, 0.264 mmol) and KO*t*Bu (29.7 mg, 0.265 mmol) were dissolved in THF (3 mL) at −78 °C and the reaction mixture was allowed to warm to room temperature. After one hour all volatiles were removed in vacuo. The product was extracted with hexane (3 mL) and the solution was concentrated under a dynamic vacuum and cooled to 5 °C, resulting in the formation of a colourless microcrystalline solid. Crystals suitable for X‐ray diffraction studies were grown from a concentrated hexane solution at room temperature. Yield: 72 mg (50 %); elemental analysis calcd (%) for C_33_H_52_NO_3_P: C 73.16, H 9.68, N 2.59; found: C 73.04, H 9.48, N 2.68. CI MS (NH_3_ carrier gas): *m*/*z* calcd for C_29_H_44_NO_3_P⋅H: 486.3137; found: 486.3140; *m*/*z* calcd for C_29_H_43_NO_2_P: 468.3026; found: 468.3051; ^1^H NMR (400.17 MHz, C_6_D_6_, 298 K): *δ*=7.40 (d, ^4^ *J*_H‐H_=2 Hz, 2 H; Ar), 7.31 (d, ^4^ *J*_H‐H_=2 Hz, 2 H; Ar), 2.89 (d, ^3^ *J*_H‐P_=1 Hz, 3 H; Me), 1.57 (s, 9 H; O*t*Bu) 1.57 (s, 18 H; *t*Bu), 1.25 ppm (s, 18 H; *t*Bu); ^13^C{^1^H} NMR (100.62 MHz, C_6_D_6_, 298 K): *δ*=149.7 (d, ^2^ *J*_C‐P_=7 Hz; Ar), 144.6 (Ar), 139.3 (Ar), 138.9 (d, ^3^ *J*_C‐P_=3 Hz; Ar), 121.5 (Ar), 118.6 (Ar), 75.7 (d, ^2^ *J*_C‐P_=15 Hz; O*C*(CH_3_)_3_), 42.5 (d, *J*_C‐P_=9 Hz; N*C*H_3_), 35.5 (*C*(CH_3_)_3_), 34.7 (*C*(CH_3_)_3_), 31.8 (OC(*C*H_3_)_3_), 31.7 (C(*C*H_3_)_3_), 30.1 ppm (C(*C*H_3_)_3_); ^31^P NMR (161.99 MHz, C_6_D_6_, 298 K) *δ*=142.8 ppm (s); ^31^P{^1^H} NMR (161.99 MHz, C_6_D_6_, 298 K) *δ*=142.8 ppm (s).

**Synthesis of (*t*BuO)As{ON(Me)O} (9 b)**: Compound **7 b** (150 mg, 0.227 mmol) was dissolved in THF (5 mL) and cooled to −78 °C. A solution of KO*t*Bu (25.5 mg, 0.227 mmol) in THF (5 mL) was then added dropwise. The reaction mixture was stirred at −78 °C for 30 mins, then 30 mins at room temperature. All volatiles were removed in vacuo and the product was extracted with hexane (5 mL) and filtered via cannula. The solution was concentrated under a dynamic vacuum yielding a crude sample of **9 b** as a white solid. Isolation of **9 b** from other impurities has not been possible, although characterisation data confirms the presence of the expected product. EI MS: *m*/*z* calcd for C_33_H_53_AsNO_3_: 586.3241; found: 586.3140; *m*/*z* calcd for C_29_H_44_AsNO_3_ [(HO)As{ON(Me)O}]: 529.2537; found: 529.2534; *m*/*z* calcd for C_29_H_43_AsNO_2_ [As{ON(Me)O}]: 512.2510 ; found: 512.2500; ^1^H NMR (400.17 MHz, C_6_D_6_, 298 K): *δ*=7.40 (d, ^3^ *J*_H‐H_=2 Hz, 2 H; Ar), 7.32 (d, ^3^ *J*_H‐H_=2 Hz, 2 H; Ar), 2.62 (s, 3 H; Me), 1.59 (s, 18 H; *t*Bu) 1.54 (s, 9 H; O*t*Bu), 1.28 ppm (s, 18 H; *t*Bu); ^13^C{^1^H} NMR (100.62 MHz, C_6_D_6_, 298 K): *δ*=151.4 (Ar), 142.9 (Ar), 139.0 (Ar), 137.9 (Ar), 121.9 (Ar), 118.0 (Ar), 73.6 (O*C*(CH_3_)_3_), 43.0 (N*C*H_3_), 35.7 (*C*(CH_3_)_3_), 34.6 (*C*(CH_3_)_3_), 33.1 (OC(*C*H_3_)_3_), 31.7 (C(*C*H_3_)_3_), 30.1 ppm (C(*C*H_3_)_3_).

**X‐ray diffraction**: Single‐crystal X‐ray diffraction data were collected using an Oxford Diffraction Supernova dual‐source diffractometer equipped with a 135 mm Atlas CCD area detector. Crystals were selected under Paratone‐N oil, mounted on micromount loops and quench‐cooled using an Oxford Cryosystems open flow N_2_ cooling device.^38^ Data were collected at 150 K using mirror monochromated Cu Kα radiation (*λ*=1.5418 Å; Oxford Diffraction Supernova). Data were processed using the CrysAlisPro package, including unit cell parameter refinement and inter‐frame scaling (which was carried out using SCALE3 ABSPACK within CrysAlisPro).^39^ Structures were subsequently solved using direct methods or using the charge flipping algorithm as implemented in the program SUPERFLIP,^40^ and refined on *F* ^2^ using the SHELXL 2013–4 package.^41^

CCDC 1489274 (**1 b**), 1489275 ([K(2,2,2‐crypt)][**2 a**]⋅1.5 toluene), 1489276 ([K(18‐crown‐6)][**2 b**]⋅THF), 1489277 ([K(18‐crown‐6)][**3 a**]⋅0.5 toluene⋅0.5 pentane), 1489278 ([K(2,2,2‐crypt)][**3 b**]), 1489279 (**4 a**), 1489280 (**5 a**), 1489281 ([**6 a**][OTf]⋅HOTf), 1489282 ([**6 b**][OTf]⋅0.5 hexane), 1489283 ([**7 a**][OTf]), 1489284 ([**7 b**][OTf]), 1489285 (**8 a**), 1489286 (**8 b**), and 1489287 (**9 a**) contain the supplementary crystallographic data. These data can be obtained free of charge by The Cambridge Crystallographic Data Centre.

**DFT calculations**: All geometry optimisations were performed using the Amsterdam Density Functional package (ADF2014.01).^42^ An TZP Slater‐type basis set of triple‐ζ quality, extended with a single polarisation functions, was used to describe all phosphorus, arsenic, nitrogen and oxygen atoms while a DZ basis set was used for all remaining atoms. Geometry optimisations were performed using the Becke88 exchange functional with Perdew86 local correlation functional.^43^, ^44^ The Grimme3 empirical dispersion correction was applied to all calculations.^45^ All structures were optimised using the gradient algorithm of Versluis and Ziegler.^46^ Stationary points were confirmed to be minima by the absence of imaginary frequencies.

## Supporting information

As a service to our authors and readers, this journal provides supporting information supplied by the authors. Such materials are peer reviewed and may be re‐organized for online delivery, but are not copy‐edited or typeset. Technical support issues arising from supporting information (other than missing files) should be addressed to the authors.

Supplementary
