# Supplementary material for: On the Ambiphilic Reactivity of Geometrically Constrained Phosphorus(III) and Arsenic(III) Compounds: Insights into Their Interaction with Ionic Substrates
Source: Chemistry. 2016 Sep 15;22(44):15712–24. doi: 10.1002/chem.201603135 (PMC5095867; doi:10.1002/chem.201603135)
Supplement: Supplementary file 1 — Supplementary [file CHEM-22-15712-s001.pdf]

CHEMISTRY
A European Journal

Supporting Information

On the Ambiphilic Reactivity of Geometrically Constrained Phosphorus(III) and Arsenic(III) Compounds: Insights into Their Interaction with Ionic Substrates

Thomas P. Robinson, Siu-Kwan Lo, Daniel De Rosa, Simon Aldridge, and Jose M. Goicoechea*[a]

chem_201603135_sm_miscellaneous_information.pdf

Supporting Information

CONTENTS:

1. Single crystal X-ray diffraction data

2. Computational details

3. NMR spectra

4. MS spectra

1. Single crystal X-ray diffraction data

Table S1. Selected X-ray data collection and refinement parameters for **1b**, [K(2,2,2-crypt)][**2a**]·1.5tol and [K(18-crown-6)][**2b**]·THF.

	**1b**	[K(2,2,2-crypt)][**2a**]·1.5tol	[K(18-crown-6)][**2b**]·THF
Formula	$C_{28}H_{40}AsNO_2$	$C_{60.5}H_{97}KN_3O_9P$	$C_{48}H_{81}AsKNO_{10}$
CCDC depository number	1489274	1489275	1489276
Fw [g mol^{-1}]	497.53	1080.48	946.15
crystal system	tetragonal	monoclinic	monoclinic
space group	*I*–4	$P2_1/c$	$P2_1/c$
a (Å)	13.7548(19)	14.3925(1)	14.6275(1)
b (Å)	13.7548(19)	17.5650(1)	21.8125(1)
c (Å)	28.807(6)	25.2124(2)	17.2391(1)
α (°)			
β (°)		102.3250(10)	113.433(1)
γ (°)			
V (Å^3)	5450.1(19)	6226.90(8)	5046.71(6)
Z	8	4	4
radiation, λ (Å)	Cu Kα (1.54178)	Cu Kα (1.54178)	Cu Kα (1.54178)
T (K)	150(2)	150(2)	150(2)
ρ_{calc} (g cm^{-3})	1.213	1.153	1.245
μ (mm^{-1})	1.270	1.416	2.082
reflections collected	6126	82613	55837
independent reflections	6111	12957	10494
parameters	318	709	6.42
R(int)	0.0242	0.0293	0.0272
R1/wR2,[a] I ≥ 2σI (%)	3.90/8.95	5.55/16.04	2.79/7.33
R1/wR2,[a] all data (%)	4.54/9.28	6.15/16.76	2.96/7.47
GOF	1.028	1.035	1.030

[a] $R1 = [\Sigma||F_o| - |F_c||]/\Sigma|F_o|$; $wR2 = \{[\Sigma w[(F_o)^2 - (F_c)^2]^2]/[\Sigma w(F_o^2)^2]\}^{1/2}$; $w = [\sigma^2(F_o)^2 + (AP)^2 + BP]^{-1}$, where $P = [(F_o)^2 + 2(F_c)^2]/3$ and the A and B values are 0.0517 and 5.72 for **1b**, 0.0979 and 3.70 for [K(2,2,2-crypt)][**2a**]·1.5tol, and 0.0384 and 2.27 for [K(18-crown-6)][**2b**]·THF.

Table S2. Selected X-ray data collection and refinement parameters for [K(18-crown-6)][**3a**]·0.5tol·0.5pent, [K(2,2,2-crypt)][**3b**], **4a** and **5a**.

	[K(18-crown-6)] [**3a**]·0.5tol·0.5pent	[K(2,2,2-crypt)][**3b**]	**4a**	**5a**
Formula	$C_{58}H_{84}KN_2O_8P$	$C_{58}H_{86}AsKN_4O_8$	$C_{32}H_{50}NO_3P$	$C_{39}H_{56}NO_3P$
CCDC depository number	1489277	1489278	1489279	1489280
Fw [g mol^{-1}]	1007.34	1081.32	527.70	617.81
crystal system	monoclinic	orthorhombic	monoclinic	monoclinic
space group	*Ia*	$Pna2_1$	$P2_1/c$	$P2_1/c$
a (Å)	16.1272(2)	30.0623(2)	10.4282(1)	11.4051(3)
b (Å)	18.4364(2)	10.6173(1)	29.6821(2)	16.7186(5)
c (Å)	20.9814(6)	18.4922(1)	20.9711(2)	19.5555(5)
α (°)				
β (°)	110.656(2)		102.014(1)	95.679(2)
γ (°)				
V (Å^3)	5837.3(2)	5902.35(8)	6349.02(10)	3710.49(18)
Z	4	4	8	4
radiation, λ (Å)	Cu Kα (1.54178)	Cu Kα (1.54178)	Cu Kα (1.54178)	Cu Kα (1.54178)
T (K)	150(2)	150(2)	150(2)	150(2)
ρ_{calc} (g cm^{-3})	1.146	1.217	1.104	1.106
μ (mm^{-1})	1.461	1.836	0.992	0.917
reflections collected	32544	71276	71331	34941
independent reflections	10556	11961	13210	7701
parameters	713	661	697	
R(int)	0.0243	0.0322	0.0255	0.0264
R1/wR2,[a] I ≥ 2σI (%)	3.45/9.39	3.02/7.88	3.77/10.12	3.81/10.13
R1/wR2,[a] all data (%)	3.55/9.56	3.04/7.92	4.28/10.58	4.31/10.61
GOF	1.006	1.045	1.031	1.034

[a] $R1 = [\Sigma||F_o| - |F_c||]/\Sigma|F_o|$; $wR2 = \{[\Sigma w[(F_o)^2 - (F_c)^2]^2]/[\Sigma w(F_o^2)^2]\}^{1/2}$; $w = [\sigma^2(F_o)^2 + (AP)^2 + BP]^{-1}$, where $P = [(F_o)^2 + 2(F_c)^2]/3$ and the A and B values are 0.0724 and 1.14 for [K(18-crown-6)][**3a**]·0.5tol·0.5pent, 0.0610 and 0.71 for [K(2,2,2-crypt)][**3b**], 0.0588 and 1.98 for **4a**, and 0.0560 and 1.23 for **5a**.

Table S3. Selected X-ray data collection and refinement parameters for [**6a**][OTf]·HOTf, [**6b**][OTf]·0.5hex and [**7a**][OTf].

	[**6a**][OTf]·HOTf	[**6b**][OTf]·0.5hex	[**7a**][OTf]
Formula	$C_{30}H_{42}F_6NO_8PS_2$	$C_{32}H_{48}AsF_3NO_5S$	$C_{30}H_{43}F_3NO_5PS$
CCDC depository number	1489281	1489282	1489283
Fw [g mol^{-1}]	753.73	690.69	617.68
crystal system	monoclinic	triclinic	monoclinic
space group	$P2_1/c$	P–1	$P2_1/n$
a (Å)	10.9709(1)	13.9248(4)	15.7666(2)
b (Å)	24.3831(4)	15.1784(4)	11.0178(1)
c (Å)	14.2349(3)	19.1969(4)	18.5524(2)
α (°)		110.964(2)	
β (°)	96.846(2)	98.355(2)	96.478(1)
γ (°)		105.897(2)	
V (Å^3)	3780.75(11)	3507.63(17)	3202.22(6)
Z	4	4	4
radiation, λ (Å)	Cu Kα (1.54178)	Cu Kα (1.54178)	Cu Kα (1.54178)
T (K)	150(2)	150(2)	150(2)
ρ_{calc} (g cm^{-3})	1.324	1.308	1.281
μ (mm^{-1})	2.342	2.321	1.844
reflections collected	42493	38893	18595
independent reflections	7858	14480	6626
parameters	441	839	370
R(int)	0.0392	0.0231	0.0163
R1/wR2,[a] I ≥ 2σI (%)	5.40/13.67	2.71/6.77	4.21/11.46
R1/wR2,[a] all data (%)	6.13/14.44	3.20/7.10	4.54/11.84
GOF	1.065	1.026	1.028

[a] $R1 = [\Sigma||F_o| - |F_c||]/\Sigma|F_o|$; $wR2 = \{[\Sigma w[(F_o)^2 - (F_c)^2]^2]/[\Sigma w(F_o^2)^2]\}^{1/2}$; $w = [\sigma^2(F_o)^2 + (AP)^2 + BP]^{-1}$, where $P = [(F_o)^2 + 2(F_c)^2]/3$ and the A and B values are 0.0581 and 3.63 for [**6a**][OTf]·HOTf, 0.0356 and 1.01 for [**6b**][OTf]·0.5hex, and 0.0625 and 1.98 for [**7a**][OTf].

Table S4. Selected X-ray data collection and refinement parameters for [**7b**][OTf], **8a** and **9a**.

	[**7b**][OTf]	**8a**	**8b**	**9a**
Formula	$C_{30}H_{43}AsF_3NO_5S$	$C_{28}H_{41}ClNO_2P$	$C_{28}H_{41}AsClNO_2$	$C_{33}H_{52}NO_3P$
CCDC depository number	1489284	1489285	1489286	1489287
Fw [g mol^{-1}]	661.63	490.04	533.99	541.72
crystal system	triclinic	orthorhombic	orthorhombic	triclinic
space group	*P*–1	*Cmc*2$_1$	*Iba*2	*P*–1
a (Å)	10.7560(2)	9.5644(3)	19.8430(3)	5.8986(2)
b (Å)	12.9728(3)	26.5541(6)	28.1484(4)	12.8549(6)
c (Å)	12.9972(3)	11.0137(3)	10.2974(1)	22.4765(10)
α (°)	77.253(2)			79.311(4)
β (°)	76.411(2)			83.904(4)
γ (°)	73.096(2)			79.925(4)
V (Å^3)	1663.86(7)	2797.19(13)	5751.60(13)	1644.11(12)
Z	2	4	8	2
radiation, λ (Å)	Cu Kα (1.54178)	Cu Kα (1.54178)	Cu Kα (1.54178)	Cu Kα (1.54178)
T (K)	150(2)	150(2)	150(2)	150(2)
ρ_{calc} (g cm^{-3})	1.321	1.164	1.233	1.094
μ (mm^{-1})	2.425	1.923	2.623	0.969
reflections collected	30746	9673	28596	14726
independent reflections	6896	3007	5313	6781
parameters	370	210	309	344
R(int)	0.0149	0.0342	0.0303	0.0285
R1/wR2,[a] I ≥ 2σI (%)	2.31/6.03	5.08/13.59	2.26/5.80	3.80/9.32
R1/wR2,[a] all data (%)	2.37/6.08	5.20/13.74	2.36/5.89	4.76/9.91
GOF	1.016	1.062	1.040	1.020

[a] $R1 = [\Sigma||F_o| - |F_c||]/\Sigma|F_o|$; $wR2 = \{[\Sigma w[(F_o)^2 - (F_c)^2]^2]/[\Sigma w(F_o^2)^2]\}^{1/2}$; $w = [\sigma^2(F_o)^2 + (AP)^2 + BP]^{-1}$, where $P = [(F_o)^2 + 2(F_c)^2]/3$ and the A and B values are 0.0328 and 0.77 for [**7b**][OTf], 0.0740 and 2.74 for **8a**, 0.0349 and 2.14 for **8b**, and 0.0522 and 0.19 for **9a**.

2. Computational details

All geometry optimizations were performed using the Amsterdam Density Functional package (ADF2014.01).[1] An TZP Slater-type basis set of triple-ζ quality, extended with one polarization function, was used to describe all phosphorus arsenic, nitrogen, oxygen and chlorine atoms, while a DZ basis set was used for all remaining atoms. Geometry optimizations were performed using the Becke88 exchange functional with Perdew86 local correlation functional.[2,3] The Grimme3 empirical dispersion correction was applied to all calculations.[4] All structures were optimized using the gradient algorithm of Versluis and Ziegler.[5]

Cartesian coordinates [Å] for the optimized computed geometry of 1a (planar).

**Atom**	***x***	***y***	***z***
1. P	0.048714	–0.006222	–0.221172
2. N	–1.704141	0.228290	–0.289844
3. O	–0.340528	–1.781444	–0.318433
4. O	0.125613	1.809858	–0.050494
5. C	–2.487393	–0.933942	–0.285199
6. C	–3.887568	–1.069332	–0.180093
7. H	–4.516386	–0.202126	–0.046233
8. C	–4.457865	–2.345533	–0.186506
9. C	–3.605970	–3.480003	–0.271332
10. H	–4.065693	–4.465296	–0.279745
11. C	–2.208048	–3.396368	–0.319913
12. C	–1.655033	–2.085127	–0.321756
13. C	–2.154929	1.555212	–0.303073
14. C	–1.062355	2.444576	–0.115463
15. C	–1.263091	3.852028	–0.043843
16. H	–2.773921	5.368985	–0.149831
17. C	–2.581283	4.300218	–0.204590
18. C	–3.679437	3.435643	–0.464852
19. H	–4.264209	1.391640	–0.787810
20. C	–3.455132	2.056561	–0.523479
21. C	–0.073419	4.802001	0.180443
22. C	–0.523854	6.282536	0.262494
23. H	0.360870	6.918526	0.429353
24. H	–1.000216	6.616724	–0.674189
25. H	–1.218996	6.450806	1.102123
26. C	0.632062	4.434505	1.518538
27. H	–0.080366	4.516313	2.356339
28. H	1.469849	5.127916	1.701608
29. H	1.027420	3.409371	1.494527

30. C	0.917363	4.658298	–1.012896
31. H	0.403626	4.891224	–1.960415
32. H	1.758707	5.360526	–0.887761
33. H	1.320380	3.637857	–1.075906
34. C	–5.082984	4.040271	–0.682571
35. C	–5.047536	4.978158	–1.924164
36. H	–6.048039	5.403097	–2.109986
37. H	–4.343411	5.813036	–1.782179
38. H	–4.730767	4.414653	–2.816746
39. C	–6.159321	2.951688	–0.928382
40. H	–5.945179	2.366927	–1.837893
41. H	–7.140375	3.433740	–1.069612
42. H	–6.247029	2.265896	–0.069292
43. C	–5.492003	4.848587	0.582318
44. H	–4.809408	5.692914	0.765433
45. H	–6.509125	5.256008	0.459043
46. H	–5.476155	4.198591	1.472138
47. C	–1.293368	–4.633393	–0.352849
48. C	–0.395771	–4.630235	0.919966
49. H	0.239684	–5.531355	0.931970
50. H	0.254069	–3.744674	0.947527
51. H	–1.024753	–4.631537	1.825475
52. C	–2.105023	–5.952680	–0.369347
53. H	–2.722820	–6.063142	0.537278
54. H	–1.405337	–6.803529	–0.399917
55. H	–2.752076	–6.021310	–1.259590
56. C	–0.415259	–4.586773	–1.637350
57. H	0.230534	–3.697937	–1.648507
58. H	0.224549	–5.483551	–1.685441
59. H	–1.057205	–4.564877	–2.533392
60. C	–5.985647	–2.552128	–0.101937
61. C	–6.755970	–1.209321	–0.049140
62. H	–7.838554	–1.411592	–0.003695
63. H	–6.491558	–0.623709	0.846143
64. H	–6.569204	–0.599811	–0.948402
65. C	–6.459280	–3.332359	–1.362007
66. H	–6.173297	–2.787459	–2.276131
67. H	–7.555528	–3.450445	–1.347096
68. H	–6.012106	–4.336983	–1.411250
69. C	–6.324585	–3.359932	1.183720
70. H	–5.970129	–2.819268	2.076109
71. H	–7.414602	–3.505290	1.266706
72. H	–5.848722	–4.352734	1.176895

TOTAL BONDING ENERGY (kJ mol^{-1}): –39837.60

Cartesian coordinates [Å] for the optimized computed geometry of 1a (pyramidal).

**Atom**	***x***	***y***	***z***
1. P	0.048445	0.090935	–0.163272
2. N	0.012635	–1.698565	–0.097645
3. O	1.486024	0.087967	–1.075273

4. O	–1.376341	0.144718	–1.098104
5. C	1.993639	–1.203981	–1.290076
6. C	3.184488	–1.489912	–1.985352
7. C	3.538091	–2.855385	–2.053314
8. H	4.451818	–3.122169	–2.578343
9. C	2.776760	–3.892151	–1.466452
10. C	1.604594	–3.552591	–0.765920
11. H	1.004929	–4.309228	–0.271876
12. C	1.214102	–2.210831	–0.697709
13. C	4.034429	–0.369318	–2.612637
14. C	5.285688	–0.928941	–3.334638
15. H	5.011017	–1.609976	–4.156934
16. H	5.957240	–1.455685	–2.636656
17. H	5.852310	–0.090509	–3.771357
18. C	4.514886	0.593590	–1.487887
19. H	3.667479	1.078663	–0.983849
20. H	5.159728	1.378799	–1.916514
21. H	5.094720	0.035187	–0.734509
22. C	3.175879	0.401173	–3.657609
23. H	2.298821	0.871347	–3.191258
24. H	2.824298	–0.290359	–4.440873
25. H	3.782202	1.191161	–4.131275
26. C	3.254530	–5.355069	–1.601296
27. C	3.359564	–5.720023	–3.110293
28. H	3.660309	–6.774597	–3.227594
29. H	4.103009	–5.095888	–3.629870
30. H	2.385597	–5.572060	–3.604300
31. C	2.275046	–6.355162	–0.936798
32. H	2.181981	–6.176695	0.147080
33. H	2.654495	–7.381125	–1.072724
34. H	1.276200	–6.305825	–1.400300
35. C	4.643621	–5.504899	–0.918190
36. H	4.577902	–5.217186	0.143653
37. H	5.401031	–4.865244	–1.397350
38. H	4.990351	–6.549960	–0.981878
39. C	–1.947866	–1.123381	–1.292912
40. C	–3.149926	–1.361448	–1.979485
41. C	–3.573552	–2.713131	–2.029605
42. H	–4.497504	–2.935853	–2.553270
43. C	–2.866280	–3.771744	–1.426003
44. C	–1.676985	–3.475811	–0.724615
45. H	–1.121820	–4.257738	–0.214004
46. C	–1.216639	–2.160651	–0.681590
47. C	–3.948062	–0.212387	–2.624592
48. C	–3.055496	0.499148	–3.682692
49. H	–2.157364	0.936408	–3.224457
50. H	–3.624948	1.307307	–4.171421
51. H	–2.736632	–0.222318	–4.452869
52. C	–4.381937	0.790342	–1.515336
53. H	–3.512027	1.241175	–1.017948

54. H	–4.988506	0.272320	–0.754318
55. H	–4.988296	1.598784	–1.956795
56. C	–5.225661	–0.723454	–3.336737
57. H	–5.752164	0.134127	–3.786599
58. H	–5.920822	–1.206892	–2.630761
59. H	–4.985270	–1.429895	–4.148156
60. C	–3.335676	–5.241327	–1.503816
61. C	–2.220510	–6.090718	–2.181973
62. H	–1.290043	–6.082256	–1.593909
63. H	–1.992448	–5.689655	–3.182705
64. H	–2.547363	–7.139087	–2.284009
65. C	–3.604065	–5.773103	–0.066472
66. H	–4.366171	–5.152663	0.432352
67. H	–2.692918	–5.753391	0.551590
68. H	–3.964845	–6.814593	–0.106333
69. C	–4.635278	–5.401492	–2.331096
70. H	–5.474045	–4.846491	–1.880451
71. H	–4.918809	–6.466378	–2.361057
72. H	–4.497249	–5.063346	–3.371008

TOTAL BONDING ENERGY (kJ mol^{-1}): –39828.16

Cartesian coordinates [Å] for the optimized computed geometry of 1b.

**Atom**	***x***	***y***	***z***
1. As	0.109513	–0.000995	–0.025517
2. N	–1.796976	–0.061919	–0.138202
3. O	–0.238878	1.942651	–0.224868
4. O	–0.132828	–1.964272	0.106839
5. C	–1.535009	2.269114	–0.238432
6. C	–2.024257	3.612117	–0.311347
7. C	–3.408813	3.787624	–0.231597
8. H	–3.806884	4.797473	–0.291942
9. C	–4.327416	2.717471	–0.040788
10. C	–3.836208	1.413508	0.019193
11. H	–4.504518	0.595428	0.249464
12. C	–2.450566	1.169890	–0.139818
13. C	–1.044121	4.791091	–0.458237
14. C	–1.783414	6.148915	–0.566717
15. H	–2.451210	6.179875	–1.443743
16. H	–1.038358	6.951901	–0.687911
17. H	–2.366772	6.372893	0.341524
18. C	–0.202264	4.599685	–1.753814
19. H	–0.868426	4.540566	–2.630270
20. H	0.398298	3.681155	–1.709390
21. H	0.479204	5.455993	–1.888321
22. C	–0.117981	4.843164	0.792524
23. H	0.487017	3.930262	0.877644
24. H	–0.725214	4.947805	1.706798
25. H	0.561112	5.709281	0.720011
26. C	–5.831557	3.031729	0.100682
27. C	–6.057655	3.892492	1.377263

28. H	–7.129298	4.121990	1.498346
29. H	–5.507101	4.845318	1.326801
30. H	–5.709329	3.346427	2.268736
31. C	–6.307862	3.813128	–1.158091
32. H	–7.395491	3.987494	–1.107221
33. H	–6.083394	3.238446	–2.071509
34. H	–5.814620	4.793949	–1.242603
35. C	–6.689144	1.745840	0.220975
36. H	–7.752899	2.022223	0.302150
37. H	–6.430155	1.166144	1.121946
38. H	–6.578235	1.102692	–0.667254
39. C	–1.397239	–2.373105	–0.039053
40. C	–2.366386	–1.332187	–0.221866
41. C	–3.707998	–1.659322	–0.536160
42. H	–4.402716	–0.879590	–0.816158
43. C	–4.113115	–2.993900	–0.561657
44. C	–3.151145	–4.008179	–0.296304
45. H	–3.484232	–5.042934	–0.308170
46. C	–1.799086	–3.746436	–0.054940
47. C	–0.763034	–4.863802	0.168194
48. C	–1.416810	–6.268645	0.151883
49. H	–1.882341	–6.492181	–0.822056
50. H	–2.172197	–6.376776	0.947736
51. H	–0.637346	–7.027401	0.327913
52. C	–0.082303	–4.666939	1.553824
53. H	0.638032	–5.481715	1.736557
54. H	–0.841950	–4.684634	2.353001
55. H	0.454835	–3.710120	1.603523
56. C	0.296328	–4.811272	–0.973104
57. H	–0.197645	–4.931730	–1.951700
58. H	1.025802	–5.628608	–0.844229
59. H	0.837960	–3.855516	–0.970548
60. C	–5.566343	–3.399150	–0.889441
61. C	–6.496730	–2.169037	–1.041569
62. H	–6.188139	–1.524373	–1.880892
63. H	–6.524810	–1.569909	–0.116319
64. H	–7.523711	–2.511637	–1.250245
65. C	–6.124816	–4.282377	0.263277
66. H	–5.570064	–5.227960	0.361615
67. H	–7.181703	–4.532390	0.071503
68. H	–6.057908	–3.744292	1.222662
69. C	–5.581049	–4.195645	–2.225387
70. H	–5.170904	–3.577473	–3.040120
71. H	–6.612152	–4.487148	–2.486104
72. H	–4.972794	–5.111295	–2.156394

Cartesian coordinates [Å] for the optimized computed geometry of 2a.

**Atom**	***x***	***y***	***z***
1. P	0.005330	0.099222	–0.197048
2. N	0.109048	–1.677216	–0.435153

3. O	1.964363	0.022782	–0.273801
4. O	–1.890570	–0.206960	–0.350967
5. O	–0.031730	–0.049729	1.498204
6. C	–0.026458	1.115288	2.375761
7. C	1.427383	1.612081	2.540438
8. H	2.066160	0.770497	2.846902
9. H	1.484004	2.412560	3.299859
10. H	1.808716	1.995467	1.582317
11. C	–0.574465	0.566008	3.712040
12. H	–1.587318	0.166371	3.549561
13. H	–0.609253	1.357664	4.479971
14. H	0.068265	–0.256007	4.064332
15. C	–0.942474	2.250730	1.860226
16. H	–1.957777	1.865785	1.693580
17. H	–0.567535	2.660547	0.909894
18. H	–0.983317	3.065332	2.604968
19. C	2.416449	–1.233611	–0.397626
20. C	3.785225	–1.616903	–0.466305
21. C	4.078849	–2.986759	–0.654035
22. H	5.122482	–3.296464	–0.683407
23. C	3.080890	–3.970390	–0.816838
24. C	1.729347	–3.568830	–0.784098
25. H	0.940118	–4.280520	–0.997989
26. C	1.393941	–2.228840	–0.522847
27. C	4.888427	–0.544894	–0.353036
28. C	6.312499	–1.153131	–0.420715
29. H	7.055066	–0.341086	–0.327770
30. H	6.488335	–1.664660	–1.382413
31. H	6.485738	–1.867337	0.402150
32. C	4.737591	0.463102	–1.530747
33. H	5.515150	1.246421	–1.466431
34. H	3.745684	0.936420	–1.503790
35. H	4.844112	–0.068445	–2.491674
36. C	4.747069	0.200049	1.004959
37. H	5.532103	0.972025	1.104858
38. H	4.844066	–0.518261	1.836402
39. H	3.762539	0.678848	1.072933
40. C	3.486886	–5.450114	–1.003781
41. C	4.276456	–5.614349	–2.334164
42. H	5.187932	–4.995538	–2.331719
43. H	3.650711	–5.288502	–3.181118
44. H	4.569828	–6.668930	–2.487733
45. C	4.378447	–5.896424	0.191875
46. H	3.841119	–5.729216	1.139708
47. H	5.317355	–5.322292	0.230445
48. H	4.633309	–6.968305	0.106867
49. C	2.257379	–6.394248	–1.048656
50. H	1.670494	–6.325423	–0.118535
51. H	2.597225	–7.438714	–1.161776
52. H	1.598478	–6.160415	–1.900413

53. C	–2.188218	–1.487266	–0.046330
54. C	–3.470146	–1.986243	0.302746
55. C	–3.569951	–3.352067	0.656799
56. H	–4.548187	–3.754208	0.915934
57. C	–2.451950	–4.210335	0.714649
58. C	–1.187329	–3.697555	0.359542
59. H	–0.300772	–4.310964	0.462549
60. C	–1.057433	–2.363083	–0.059369
61. C	–4.688422	–1.039822	0.307866
62. C	–6.001147	–1.766994	0.694357
63. H	–5.945231	–2.186535	1.713094
64. H	–6.235932	–2.578299	–0.015317
65. H	–6.834606	–1.043225	0.670889
66. C	–4.873125	–0.430774	–1.112324
67. H	–5.738273	0.257011	–1.124992
68. H	–5.048724	–1.237529	–1.843964
69. H	–3.971458	0.121062	–1.414123
70. C	–4.442340	0.097642	1.340903
71. H	–4.283055	–0.332727	2.343646
72. H	–5.310054	0.781823	1.376214
73. H	–3.546962	0.665517	1.059555
74. C	–2.629174	–5.669318	1.195998
75. C	–3.118115	–5.664599	2.674013
76. H	–3.229012	–6.696294	3.054825
77. H	–4.089002	–5.152145	2.767282
78. H	–2.391069	–5.124912	3.302991
79. C	–1.306084	–6.474326	1.129168
80. H	–0.916706	–6.513296	0.099034
81. H	–1.486253	–7.509904	1.466743
82. H	–0.534702	–6.035816	1.782572
83. C	–3.673995	–6.396049	0.300570
84. H	–3.347679	–6.367603	–0.752020
85. H	–4.661162	–5.911238	0.361115
86. H	–3.787912	–7.449822	0.613990

Cartesian coordinates [Å] for the optimized computed geometry of 2b.

**Atom**	***x***	***y***	***z***
1. As	–0.023323	0.241943	–0.333513
2. N	0.090574	–1.664856	–0.574457
3. O	2.022751	0.070601	–0.395900
4. O	–2.013645	–0.188977	–0.511378
5. O	–0.095848	–0.002676	1.521142
6. C	–0.040669	1.121341	2.431761
7. C	1.400207	1.683385	2.493056
8. H	2.097310	0.862867	2.717423
9. H	1.488636	2.463305	3.270680
10. H	1.688577	2.114457	1.521770
11. C	–0.436732	0.512902	3.799233
12. H	–1.451510	0.090878	3.729034
13. H	–0.407386	1.274910	4.597125

14. H	0.258705	–0.304057	4.049239
15. C	–1.049468	2.231747	2.043706
16. H	–2.052850	1.795251	1.935482
17. H	–0.769495	2.702976	1.087485
18. H	–1.078229	3.016020	2.821040
19. C	2.421117	–1.210214	–0.480268
20. C	3.785165	–1.621653	–0.483971
21. C	4.067658	–2.995985	–0.639793
22. H	5.106383	–3.320728	–0.619257
23. C	3.062739	–3.966192	–0.833860
24. C	1.719625	–3.544505	–0.854010
25. H	0.927737	–4.247522	–1.088105
26. C	1.383060	–2.196459	–0.617259
27. C	4.905180	–0.571136	–0.325468
28. C	6.316905	–1.212416	–0.299728
29. H	7.070873	–0.415329	–0.176187
30. H	6.538980	–1.744772	–1.240051
31. H	6.424983	–1.914819	0.544310
32. C	4.848961	0.419720	–1.525758
33. H	5.639294	1.186297	–1.426328
34. H	3.868604	0.915438	–1.565670
35. H	5.001505	–0.130506	–2.469964
36. C	4.708298	0.200663	1.011120
37. H	5.518060	0.939991	1.151012
38. H	4.720990	–0.507610	1.856746
39. H	3.743191	0.721746	1.009138
40. C	3.457532	–5.450622	–1.007956
41. C	4.258664	–5.625172	–2.330256
42. H	5.174202	–5.012369	–2.320101
43. H	3.643720	–5.296952	–3.184267
44. H	4.545463	–6.682445	–2.478101
45. C	4.334297	–5.900975	0.196862
46. H	3.792727	–5.722334	1.140154
47. H	5.281172	–5.340677	0.239343
48. H	4.575695	–6.976568	0.118705
49. C	2.220604	–6.383672	–1.060108
50. H	1.626076	–6.302876	–0.136206
51. H	2.551554	–7.432168	–1.163934
52. H	1.571925	–6.149540	–1.919553
53. C	–2.233634	–1.475711	–0.174302
54. C	–3.496656	–1.999214	0.217352
55. C	–3.561809	–3.351608	0.620136
56. H	–4.526017	–3.764396	0.912451
57. C	–2.424925	–4.182384	0.688771
58. C	–1.180695	–3.652051	0.296689
59. H	–0.278199	–4.239680	0.412674
60. C	–1.077713	–2.330435	–0.173483
61. C	–4.737499	–1.080186	0.230625
62. C	–6.022173	–1.827594	0.670424
63. H	–5.930678	–2.216744	1.698547

64. H	–6.255743	–2.663567	–0.010558
65. H	–6.872941	–1.124093	0.649058
66. C	–4.977919	–0.511997	–1.198043
67. H	–5.860655	0.153348	–1.202644
68. H	–5.153150	–1.341240	–1.904423
69. H	–4.099303	0.054466	–1.537727
70. C	–4.494223	0.087136	1.231397
71. H	–4.296747	–0.317893	2.237881
72. H	–5.379466	0.747863	1.275855
73. H	–3.621712	0.671789	0.914589
74. C	–2.561287	–5.624751	1.228278
75. C	–3.009697	–5.572684	2.717882
76. H	–3.088202	–6.591115	3.140587
77. H	–3.988477	–5.077050	2.817873
78. H	–2.278237	–4.994018	3.305349
79. C	–1.226356	–6.408774	1.153028
80. H	–0.864724	–6.474829	0.114481
81. H	–1.379239	–7.435716	1.528665
82. H	–0.445761	–5.936539	1.770879
83. C	–3.616318	–6.403558	0.390434
84. H	–3.315631	–6.414942	–0.670112
85. H	–4.609469	–5.932073	0.454812
86. H	–3.705796	–7.444617	0.750687

Cartesian coordinates [Å] for the optimized computed geometry of 3a.

**Atom**	***x***	***y***	***z***
1. P	0.112810	0.194695	–0.256864
2. O	–1.757168	0.060061	–0.503655
3. O	1.991840	–0.048892	–0.031744
4. N	0.071521	–1.610022	–0.216897
5. N	–0.110383	0.548815	1.575559
6. C	–2.217504	–1.207388	–0.489719
7. C	–3.577325	–1.579441	–0.586255
8. C	–3.887115	–2.959024	–0.483309
9. H	–4.931255	–3.257594	–0.511633
10. C	–2.896787	–3.948554	–0.360353
11. C	–1.539675	–3.553844	–0.341357
12. H	–0.765967	–4.312138	–0.345876
13. C	–1.196280	–2.197962	–0.342609
14. C	–4.657415	–0.503118	–0.818552
15. C	–4.349264	0.226728	–2.158969
16. H	–5.118241	0.993045	–2.363772
17. H	–4.342348	–0.502682	–2.986365
18. H	–3.364079	0.712607	–2.115650
19. C	–4.659381	0.522451	0.351424
20. H	–5.428386	1.296601	0.175302
21. H	–3.681356	1.015600	0.440879
22. H	–4.893968	0.011340	1.300536
23. C	–6.078873	–1.112991	–0.920454
24. H	–6.807021	–0.302148	–1.095890

25. H	–6.366553	–1.628323	0.011354
26. H	–6.154026	–1.824152	–1.760083
27. C	–3.219669	–5.452209	–0.219739
28. C	–2.628158	–6.231447	–1.430102
29. H	–3.055947	–5.842308	–2.369159
30. H	–2.857205	–7.309477	–1.349607
31. H	–1.534480	–6.115725	–1.486002
32. C	–4.743628	–5.725789	–0.167741
33. H	–5.243364	–5.394658	–1.093558
34. H	–5.209929	–5.214153	0.691211
35. H	–4.919715	–6.810633	–0.057022
36. C	–2.591591	–5.984327	1.103775
37. H	–2.957746	–5.391805	1.957972
38. H	–1.493940	–5.901604	1.090652
39. H	–2.853572	–7.046817	1.259205
40. C	2.295204	–1.279258	0.429284
41. C	3.510653	–1.645314	1.055943
42. C	3.635013	–2.978763	1.520180
43. H	4.574497	–3.280484	1.974621
44. C	2.591918	–3.916947	1.418354
45. C	1.373393	–3.514393	0.828834
46. H	0.530911	–4.192625	0.853713
47. C	1.222526	–2.220922	0.316206
48. C	4.623788	–0.596099	1.254466
49. C	5.030463	–0.002103	–0.125968
50. H	4.171476	0.490227	–0.602792
51. H	5.385084	–0.808893	–0.790286
52. H	5.841525	0.736943	0.003971
53. C	5.894026	–1.205718	1.903095
54. H	6.324791	–2.005658	1.276263
55. H	5.683531	–1.610227	2.907428
56. H	6.654062	–0.413117	2.014016
57. C	4.099616	0.535393	2.186132
58. H	3.216931	1.024657	1.755712
59. H	4.879963	1.303001	2.335549
60. H	3.823647	0.124554	3.171657
61. C	2.723074	–5.380456	1.903555
62. C	2.404700	–6.338680	0.717036
63. H	1.361188	–6.231896	0.382839
64. H	2.562864	–7.390787	1.014986
65. H	3.058038	–6.103443	–0.138960
66. C	4.151374	–5.708025	2.409417
67. H	4.194538	–6.763090	2.732294
68. H	4.425852	–5.080172	3.272959
69. H	4.899250	–5.561779	1.612845
70. C	1.726528	–5.656394	3.065844
71. H	0.696096	–5.385893	2.787550
72. H	2.006336	–5.062392	3.950033
73. H	1.744671	–6.726283	3.342338
74. C	0.607627	1.604605	2.142703

75. C	0.782550	1.730575	3.551594
76. H	0.317319	1.003668	4.216663
77. C	1.547443	2.771979	4.091873
78. H	1.672158	2.826699	5.176124
79. C	2.149376	3.738453	3.267728
80. H	2.757549	4.537582	3.694358
81. C	1.933572	3.659027	1.879514
82. H	2.369608	4.408998	1.215393
83. C	1.174094	2.625917	1.323919
84. H	1.031651	2.583428	0.246100
85. C	–0.474114	–0.565062	2.402742
86. C	–1.820737	–0.976318	2.454949
87. H	–2.571931	–0.363443	1.965587
88. C	–2.171674	–2.186762	3.072658
89. H	–3.207477	–2.527353	3.031510
90. C	–1.191230	–2.960399	3.713432
91. H	–1.456955	–3.908679	4.183961
92. C	0.140345	–2.507099	3.742169
93. H	0.912693	–3.095987	4.235201
94. C	0.502690	–1.330251	3.079650
95. H	1.549234	–1.025769	3.032996

Cartesian coordinates [Å] for the optimized computed geometry of 3b.

**Atom**	***x***	***y***	***z***
1. As	0.091818	0.326401	–0.393782
2. O	–1.894283	0.077825	–0.623181
3. O	2.060424	–0.011049	–0.083281
4. N	0.050893	–1.602052	–0.318835
5. N	–0.163561	0.732197	1.621761
6. C	–2.278232	–1.211879	–0.551945
7. C	–3.632225	–1.630000	–0.568167
8. C	–3.903275	–3.012844	–0.431081
9. H	–4.939494	–3.336345	–0.393895
10. C	–2.882731	–3.976165	–0.359847
11. C	–1.541166	–3.543447	–0.413259
12. H	–0.748865	–4.280491	–0.464232
13. C	–1.222921	–2.178648	–0.420633
14. C	–4.758885	–0.590028	–0.750711
15. C	–4.568073	0.110861	–2.128001
16. H	–5.369381	0.852720	–2.295908
17. H	–4.602908	–0.640962	–2.934287
18. H	–3.595207	0.620901	–2.168045
19. C	–4.718510	0.465800	0.391820
20. H	–5.519857	1.212370	0.243718
21. H	–3.752131	0.987979	0.409476
22. H	–4.878894	–0.025492	1.366234
23. C	–6.165627	–1.242390	–0.738506
24. H	–6.926538	–0.456440	–0.885849
25. H	–6.373468	–1.736366	0.225683
26. H	–6.279655	–1.979635	–1.550915

27. C	–3.156382	–5.484485	–0.171674
28. C	–2.576767	–6.284454	–1.373067
29. H	–3.047698	–5.946247	–2.311098
30. H	–2.765357	–7.365533	–1.245367
31. H	–1.489634	–6.135504	–1.468297
32. C	–4.669621	–5.799931	–0.066709
33. H	–5.204381	–5.509416	–0.985993
34. H	–5.126307	–5.277711	0.790564
35. H	–4.810545	–6.884825	0.081482
36. C	–2.474980	–5.957808	1.147520
37. H	–2.836497	–5.353824	1.995140
38. H	–1.381802	–5.839448	1.099640
39. H	–2.696645	–7.022954	1.340995
40. C	2.288160	–1.255754	0.380514
41. C	3.482967	–1.646029	1.039916
42. C	3.577321	–2.975442	1.516634
43. H	4.501819	–3.288636	1.992908
44. C	2.522524	–3.897492	1.397396
45. C	1.326962	–3.476073	0.782064
46. H	0.473926	–4.140105	0.797139
47. C	1.197328	–2.185141	0.252443
48. C	4.617163	–0.619793	1.251026
49. C	5.080576	–0.064983	–0.128482
50. H	4.251732	0.446892	–0.636907
51. H	5.425426	–0.895782	–0.767923
52. H	5.912033	0.649389	0.010413
53. C	5.854198	–1.249580	1.944783
54. H	6.285252	–2.066819	1.340934
55. H	5.605545	–1.636070	2.947736
56. H	6.628166	–0.472494	2.067928
57. C	4.105837	0.544268	2.149587
58. H	3.238478	1.043586	1.700015
59. H	4.901180	1.298069	2.290436
60. H	3.813225	0.163599	3.142285
61. C	2.622765	–5.366255	1.874730
62. C	2.339528	–6.308270	0.665814
63. H	1.309779	–6.186353	0.295307
64. H	2.476642	–7.365390	0.956802
65. H	3.025593	–6.069839	–0.163474
66. C	4.029213	–5.711231	2.427799
67. H	4.049187	–6.767052	2.750318
68. H	4.282080	–5.086771	3.300672
69. H	4.804955	–5.573188	1.656398
70. C	1.582051	–5.650839	2.995067
71. H	0.565493	–5.365807	2.683469
72. H	1.831938	–5.076773	3.901210
73. H	1.578581	–6.725866	3.251686
74. C	0.637637	1.733164	2.169787
75. C	0.881508	1.838112	3.572787
76. H	0.415085	1.123288	4.250069

77. C	1.704864	2.844402	4.089986
78. H	1.875908	2.882706	5.168903
79. C	2.307722	3.797935	3.250234
80. H	2.961091	4.570450	3.658644
81. C	2.030420	3.742402	1.871947
82. H	2.466860	4.482092	1.196838
83. C	1.209288	2.743907	1.341307
84. H	1.019258	2.728443	0.268714
85. C	–0.438918	–0.437245	2.394046
86. C	–1.751957	–0.951854	2.403993
87. H	–2.538932	–0.375933	1.924884
88. C	–2.022378	–2.210572	2.962732
89. H	–3.030574	–2.622308	2.884545
90. C	–0.998646	–2.939365	3.588434
91. H	–1.202465	–3.922669	4.015913
92. C	0.297785	–2.393472	3.655244
93. H	1.108421	–2.950339	4.124740
94. C	0.581659	–1.166848	3.051110
95. H	1.606278	–0.797466	3.025916

Cartesian coordinates [Å] for the optimized computed geometry of 4a.

**Atom**	***x***	***y***	***z***
1. P	0.011815	0.017117	0.007277
2. H	1.325849	0.028934	–0.513247
3. N	–1.740406	0.017306	–0.023976
4. O	–0.032800	1.674455	–0.404228
5. O	–0.032437	–1.619957	–0.486319
6. O	0.182750	–0.024679	1.616237
7. C	1.497002	–0.044904	2.330344
8. C	2.285952	–1.309933	1.946695
9. H	2.610928	–1.294142	0.896711
10. H	1.663528	–2.200880	2.108968
11. H	3.184778	–1.388213	2.579742
12. C	1.076266	–0.097525	3.809653
13. H	0.476068	–0.999599	4.000272
14. H	0.468284	0.783701	4.061916
15. H	1.965669	–0.115628	4.457827
16. C	2.272928	1.251375	2.032380
17. H	2.591915	1.311034	0.982112
18. H	3.174328	1.294961	2.665100
19. H	1.642855	2.122862	2.258471
20. C	–1.291992	2.247614	–0.308035
21. C	–1.574441	3.616594	–0.452317
22. C	–2.936809	3.979175	–0.354917
23. H	–3.197330	5.030294	–0.447219
24. C	–3.977023	3.046536	–0.142666
25. C	–3.654272	1.682021	–0.013910
26. H	–4.429262	0.946172	0.153889
27. C	–2.310540	1.287111	–0.097687
28. C	–0.450205	4.636521	–0.716254

29. C	–0.992493	6.084614	–0.805082
30. H	–1.476729	6.394259	0.135727
31. H	–1.709372	6.201701	–1.634443
32. H	–0.152382	6.773555	–0.992378
33. C	0.584252	4.576425	0.445944
34. H	0.082502	4.760533	1.410103
35. H	1.360806	5.346178	0.299275
36. H	1.073680	3.594948	0.487042
37. C	0.239841	4.291357	–2.068169
38. H	1.034087	5.025898	–2.283901
39. H	–0.498472	4.320935	–2.886477
40. H	0.689873	3.288760	–2.044056
41. C	–5.436340	3.547313	–0.050968
42. C	–6.445714	2.383583	0.121518
43. H	–6.405530	1.685708	–0.731358
44. H	–7.468721	2.791394	0.171975
45. H	–6.266524	1.825320	1.055271
46. C	–5.573258	4.494604	1.175460
47. H	–4.924883	5.379336	1.077409
48. H	–5.285027	3.963722	2.097291
49. H	–6.615137	4.842516	1.277051
50. C	–5.800647	4.311271	–1.356444
51. H	–5.649117	3.659804	–2.232415
52. H	–5.179291	5.210273	–1.488948
53. H	–6.856245	4.629998	–1.329430
54. C	–1.284918	–2.204470	–0.375725
55. C	–1.563685	–3.567749	–0.547122
56. C	–2.921973	–3.948906	–0.399960
57. H	–3.171495	–4.998258	–0.516320
58. C	–3.952716	–3.035139	–0.106191
59. C	–3.632838	–1.667198	0.041790
60. H	–4.408804	–0.949866	0.283516
61. C	–2.304498	–1.254922	–0.104593
62. C	–0.445917	–4.571361	–0.891472
63. C	–0.981011	–6.020697	–1.001884
64. H	–0.144104	–6.695392	–1.246635
65. H	–1.732289	–6.119815	–1.802090
66. H	–1.420941	–6.363849	–0.051022
67. C	0.639983	–4.539560	0.223947
68. H	1.406594	–5.307751	0.026107
69. H	0.181231	–4.743830	1.205130
70. H	1.133297	–3.560254	0.264175
71. C	0.182356	–4.178374	–2.260628
72. H	0.976950	–4.895159	–2.529760
73. H	0.619923	–3.170422	–2.226044
74. H	–0.588873	–4.195208	–3.048345
75. C	–5.426767	–3.466740	0.065064
76. C	–5.626425	–4.985168	–0.166782
77. H	–5.329808	–5.280413	–1.186748
78. H	–6.692850	–5.236367	–0.042514

79. H	–5.056115	–5.584795	0.561441
80. C	–6.306692	–2.699829	–0.965675
81. H	–5.939584	–2.884504	–1.988412
82. H	–6.288439	–1.612946	–0.789946
83. H	–7.355195	–3.035574	–0.897842
84. C	–5.892724	–3.131583	1.512656
85. H	–5.851837	–2.049643	1.713523
86. H	–5.244841	–3.637399	2.246965
87. H	–6.932471	–3.466617	1.666129

Cartesian coordinates [Å] for the optimized computed geometry of 5a.

**Atom**	***x***	***y***	***z***
1. P	–0.160005	0.157747	–0.129634
2. N	0.370190	–1.381763	0.450654
3. O	–1.594096	–0.056160	0.901159
4. O	1.346335	0.201419	–1.079821
5. O	–1.039354	0.488539	–1.434211
6. C	–1.641420	–1.253377	1.563081
7. C	–2.666803	–1.682165	2.422099
8. C	–2.517437	–2.978968	2.977023
9. H	–3.286660	–3.333056	3.655746
10. C	–1.432528	–3.826821	2.682904
11. C	–0.419513	–3.357075	1.816175
12. H	0.407948	–4.000194	1.530816
13. C	–0.514158	–2.063865	1.302621
14. C	–3.851330	–0.758466	2.762720
15. C	–4.618459	–0.393317	1.459513
16. H	–4.963423	–1.309878	0.953899
17. H	–3.974023	0.169450	0.770719
18. H	–5.497510	0.228160	1.702015
19. C	–3.303828	0.534197	3.435624
20. H	–2.641549	1.085455	2.752949
21. H	–2.741678	0.277055	4.348522
22. H	–4.139377	1.198297	3.715939
23. C	–4.848327	–1.426166	3.742258
24. H	–5.294923	–2.336477	3.309202
25. H	–5.667803	–0.720844	3.957147
26. H	–4.368655	–1.682167	4.701343
27. C	–1.296335	–5.247670	3.274153
28. C	–2.450806	–5.596351	4.245708
29. H	–2.479178	–4.906581	5.104999
30. H	–2.300726	–6.615225	4.639241
31. H	–3.428180	–5.576402	3.736814
32. C	–1.307850	–6.286537	2.114921
33. H	–0.470707	–6.126323	1.417337
34. H	–2.246293	–6.205448	1.542758
35. H	–1.224671	–7.310714	2.516584
36. C	0.044622	–5.348612	4.059022
37. H	0.914195	–5.186664	3.403375
38. H	0.147643	–6.347373	4.515976

39. H	0.074689	–4.588847	4.856937
40. C	2.280668	–0.702916	–0.640551
41. C	3.647573	–0.720136	–0.985653
42. C	4.456119	–1.620583	–0.255413
43. H	5.518214	–1.659587	–0.486629
44. C	3.964343	–2.459737	0.768836
45. C	2.582724	–2.463408	1.036280
46. H	2.170983	–3.054241	1.845526
47. C	1.752994	–1.615338	0.296814
48. C	4.202038	0.186805	–2.102195
49. C	4.071853	1.682669	–1.700847
50. H	4.451282	2.322794	–2.516030
51. H	4.667557	1.890139	–0.797075
52. H	3.021934	1.949377	–1.513666
53. C	3.390190	–0.071827	–3.405138
54. H	3.821426	0.504275	–4.241246
55. H	2.343275	0.234251	–3.277426
56. H	3.415608	–1.143269	–3.661562
57. C	5.694419	–0.107115	–2.397519
58. H	6.034254	0.541064	–3.221921
59. H	5.844664	–1.153905	–2.708410
60. H	6.333984	0.104274	–1.524827
61. C	4.942965	–3.361851	1.553408
62. C	5.580524	–4.389253	0.573410
63. H	4.791367	–4.983431	0.084210
64. H	6.255564	–5.073158	1.115875
65. H	6.163980	–3.886451	–0.213998
66. C	6.055370	–2.489285	2.201951
67. H	5.609519	–1.767607	2.904242
68. H	6.625191	–1.926144	1.446926
69. H	6.765626	–3.126413	2.756449
70. C	4.232088	–4.146883	2.685402
71. H	3.472297	–4.837047	2.283747
72. H	3.751932	–3.467481	3.408780
73. H	4.973789	–4.752257	3.232038
74. C	–1.538076	–0.580425	–2.380772
75. C	–0.374625	–1.440605	–2.906075
76. H	0.077454	–2.056299	–2.115262
77. H	0.406112	–0.809922	–3.347175
78. H	–0.763780	–2.118834	–3.683536
79. C	–2.157954	0.257963	–3.510210
80. H	–1.391663	0.902049	–3.965873
81. H	–2.964200	0.892927	–3.113994
82. H	–2.574423	–0.402888	–4.285538
83. C	–2.601959	–1.439033	–1.673874
84. H	–2.171758	–2.074977	–0.885598
85. H	–3.077793	–2.100575	–2.416519
86. H	–3.372444	–0.799771	–1.225310
87. C	0.313317	1.674221	0.818096
88. H	0.824618	2.382947	0.152527

89. H	–0.582277	2.140354	1.251173
90. C	1.263189	1.149859	1.898750
91. C	2.656327	1.198512	1.693872
92. H	3.050648	1.737704	0.835008
93. C	3.533606	0.528336	2.558887
94. H	4.606217	0.556626	2.364654
95. C	3.026132	–0.200111	3.645098
96. H	3.703310	–0.735993	4.311224
97. C	1.641135	–0.227484	3.879959
98. H	1.239169	–0.786050	4.726753
99. C	0.764024	0.450086	3.018402
100. H	–0.309819	0.401673	3.198019

Cartesian coordinates [Å] for the optimized computed geometry of 6a.

**Atom**	***x***	***y***	***z***
1. P	0.080870	0.005333	0.146345
2. N	–1.882498	–0.040702	–0.340681
3. H	–2.495474	–0.060494	0.496239
4. O	0.248597	1.328493	–0.807837
5. O	0.299935	–1.350601	–0.750866
6. C	–0.900372	1.842880	–1.460381
7. C	–0.886249	2.975900	–2.291300
8. C	–2.150509	3.364633	–2.800219
9. H	–2.179609	4.226438	–3.458562
10. C	–3.364977	2.711944	–2.500235
11. C	–3.326472	1.598966	–1.632230
12. H	–4.237987	1.071375	–1.359580
13. C	–2.091883	1.181830	–1.141900
14. C	0.412336	3.742083	–2.609575
15. C	0.991003	4.320638	–1.283424
16. H	1.890121	4.918015	–1.503365
17. H	1.275483	3.529021	–0.575826
18. H	0.250152	4.975779	–0.797248
19. C	1.428176	2.772068	–3.281724
20. H	2.333669	3.328261	–3.572278
21. H	0.988874	2.326620	–4.189281
22. H	1.733758	1.960734	–2.606543
23. C	0.155082	4.922795	–3.578532
24. H	1.110612	5.432750	–3.778963
25. H	–0.528294	5.670710	–3.144605
26. H	–0.243393	4.580771	–4.547798
27. C	–4.717150	3.138440	–3.109637
28. C	–5.280944	1.944765	–3.936877
29. H	–6.217441	2.240820	–4.434789
30. H	–5.503377	1.073245	–3.301084
31. H	–4.557156	1.634873	–4.707811
32. C	–5.702296	3.511659	–1.965288
33. H	–5.288001	4.325195	–1.348870
34. H	–5.918054	2.655198	–1.306280
35. H	–6.659215	3.850435	–2.392357

36. C	–4.573205	4.358994	–4.051236
37. H	–4.207367	5.248832	–3.514877
38. H	–5.560455	4.611428	–4.467602
39. H	–3.905111	4.145394	–4.901340
40. C	–0.817016	–1.895425	–1.432573
41. C	–0.749023	–3.035153	–2.259114
42. C	–1.984246	–3.459035	–2.794379
43. H	–1.978514	–4.329450	–3.444251
44. C	–3.227818	–2.831531	–2.538971
45. C	–3.243891	–1.713607	–1.685491
46. H	–4.171965	–1.202440	–1.446181
47. C	–2.031107	–1.265596	–1.154674
48. C	0.578922	–3.763704	–2.539934
49. C	1.581594	–2.766703	–3.190925
50. H	2.508752	–3.297206	–3.458245
51. H	1.847842	–1.945081	–2.511622
52. H	1.151334	–2.336900	–4.109830
53. C	1.140794	–4.319833	–1.197762
54. H	2.062151	–4.890851	–1.392711
55. H	0.408038	–4.994905	–0.727519
56. H	1.384507	–3.517877	–0.486712
57. C	0.379864	–4.954750	–3.510167
58. H	1.354418	–5.434705	–3.687520
59. H	–0.006810	–4.628264	–4.489145
60. H	–0.288651	–5.722517	–3.089210
61. C	–4.509618	–3.380500	–3.199255
62. C	–4.770931	–4.819101	–2.665088
63. H	–3.946541	–5.504293	–2.916283
64. H	–5.693779	–5.223761	–3.110994
65. H	–4.886810	–4.810015	–1.569198
66. C	–5.746474	–2.501689	–2.887569
67. H	–5.975441	–2.477704	–1.810125
68. H	–6.627910	–2.922282	–3.394989
69. H	–5.615956	–1.473379	–3.261391
70. C	–4.311903	–3.406096	–4.743889
71. H	–3.511380	–4.096855	–5.048592
72. H	–4.060527	–2.400059	–5.117565
73. H	–5.240604	–3.738742	–5.234316

Cartesian coordinates [Å] for the optimized computed geometry of 6b.

**Atom**	***x***	***y***	***z***
1. As	0.166944	0.020729	0.281418
2. N	–1.866256	–0.039702	–0.336193
3. H	–2.525587	–0.067653	0.464656
4. O	0.312490	1.427780	–0.843394
5. O	0.368365	–1.474910	–0.708419
6. C	–0.871210	1.873716	–1.455990
7. C	–0.902986	3.009152	–2.294938
8. C	–2.175797	3.370632	–2.794762
9. H	–2.227054	4.229053	–3.455670

10. C	–3.375523	2.697937	–2.481272
11. C	–3.302484	1.592308	–1.611191
12. H	–4.202035	1.050611	–1.325793
13. C	–2.054069	1.189960	–1.134244
14. C	0.370907	3.812350	–2.624632
15. C	0.944347	4.409014	–1.304562
16. H	1.818719	5.038548	–1.533458
17. H	1.264520	3.625977	–0.603019
18. H	0.185899	5.036890	–0.809532
19. C	1.409086	2.876240	–3.309250
20. H	2.298588	3.458202	–3.597279
21. H	0.977078	2.429707	–4.219471
22. H	1.736225	2.065131	–2.644310
23. C	0.071388	4.987332	–3.589338
24. H	1.010904	5.521313	–3.800310
25. H	–0.625856	5.716969	–3.147420
26. H	–0.328911	4.636391	–4.554375
27. C	–4.739685	3.090052	–3.087777
28. C	–5.270657	1.882747	–3.916676
29. H	–6.206970	2.157516	–4.427067
30. H	–5.483696	1.011986	–3.276956
31. H	–4.531641	1.582144	–4.676672
32. C	–5.737019	3.435252	–1.945710
33. H	–5.352453	4.267914	–1.335542
34. H	–5.922196	2.576517	–1.280325
35. H	–6.705905	3.735378	–2.374667
36. C	–4.627570	4.315599	–4.028087
37. H	–4.273848	5.210893	–3.491921
38. H	–5.623131	4.550089	–4.435713
39. H	–3.962334	4.116737	–4.883966
40. C	–0.766078	–1.933122	–1.398088
41. C	–0.726441	–3.076480	–2.233937
42. C	–1.958394	–3.467578	–2.793676
43. H	–1.962673	–4.336988	–3.444734
44. C	–3.190276	–2.809584	–2.561569
45. C	–3.188139	–1.692841	–1.710135
46. H	–4.106527	–1.154392	–1.494043
47. C	–1.974458	–1.268237	–1.155214
48. C	0.584089	–3.843882	–2.497599
49. C	1.625344	–2.876889	–3.132780
50. H	2.541500	–3.433324	–3.385103
51. H	1.901673	–2.061526	–2.450136
52. H	1.221222	–2.436642	–4.058744
53. C	1.111177	–4.425737	–1.152222
54. H	2.012739	–5.029635	–1.339823
55. H	0.348690	–5.075687	–0.693354
56. H	1.375170	–3.635529	–0.435684
57. C	0.364839	–5.025906	–3.475458
58. H	1.327603	–5.534231	–3.639155
59. H	0.003527	–4.685075	–4.459459

60. H	–0.333114	–5.774651	–3.067145
61. C	–4.473098	–3.322436	–3.249024
62. C	–4.779629	–4.757551	–2.731440
63. H	–3.967407	–5.460837	–2.970954
64. H	–5.702456	–5.136505	–3.199021
65. H	–4.917841	–4.753191	–1.638622
66. C	–5.694230	–2.417341	–2.953821
67. H	–5.938803	–2.389159	–1.879258
68. H	–6.577855	–2.818528	–3.474433
69. H	–5.534744	–1.392090	–3.324839
70. C	–4.247218	–3.342782	–4.788961
71. H	–3.451020	–4.043615	–5.082165
72. H	–3.973837	–2.339387	–5.152677
73. H	–5.171439	–3.659632	–5.297512

Cartesian coordinates [Å] for the optimized computed geometry of 7a.

**Atom**	***x***	***y***	***z***
1. P	–0.068576	0.066597	0.031413
2. N	0.015590	–1.910743	–0.175187
3. C	–0.056346	–2.649937	1.135578
4. H	0.738380	–2.266804	1.789024
5. H	0.118776	–3.718315	0.957345
6. H	–1.047247	–2.492421	1.578795
7. O	–0.674791	0.132183	–1.514041
8. O	1.550362	0.160722	–0.139403
9. C	–1.423021	–1.023817	–1.854393
10. C	–2.408348	–1.059123	–2.859763
11. C	–2.998963	–2.323896	–3.070942
12. H	–3.759197	–2.401149	–3.843268
13. C	–2.679450	–3.493576	–2.341997
14. C	–1.701370	–3.402770	–1.331057
15. H	–1.431702	–4.267137	–0.729262
16. C	–1.095639	–2.165303	–1.112200
17. C	–2.795874	0.192277	–3.667418
18. C	–3.314284	1.288431	–2.691738
19. H	–2.529541	1.628183	–2.001837
20. H	–4.163704	0.907366	–2.102948
21. H	–3.657827	2.162059	–3.267072
22. C	–3.918460	–0.115273	–4.688704
23. H	–4.841614	–0.454302	–4.193040
24. H	–3.605540	–0.865082	–5.432207
25. H	–4.162864	0.805718	–5.238821
26. C	–1.547085	0.694543	–4.448364
27. H	–0.735800	1.005435	–3.775002
28. H	–1.824687	1.560482	–5.069021
29. H	–1.167255	–0.100116	–5.109677
30. C	–3.409840	–4.811332	–2.668200
31. C	–2.898031	–5.991149	–1.806539
32. H	–1.825931	–6.186104	–1.975192
33. H	–3.441898	–6.906160	–2.086514

34. H	–3.077953	–5.824129	–0.731959
35. C	–4.930521	–4.624895	–2.393800
36. H	–5.370204	–3.842214	–3.030832
37. H	–5.101593	–4.347125	–1.341394
38. H	–5.466716	–5.564876	–2.598896
39. C	–3.173486	–5.160104	–4.167159
40. H	–3.602091	–4.401873	–4.840002
41. H	–3.649598	–6.123842	–4.406275
42. H	–2.095761	–5.239292	–4.381352
43. C	2.125258	–0.936530	–0.842885
44. C	3.391097	–0.910555	–1.446800
45. C	3.772551	–2.111717	–2.093740
46. H	4.738193	–2.128986	–2.587881
47. C	2.969271	–3.270972	–2.150824
48. C	1.717905	–3.251640	–1.497661
49. H	1.050464	–4.110754	–1.532043
50. C	1.324685	–2.085359	–0.853945
51. C	4.289139	0.339187	–1.396632
52. C	4.613801	0.659780	0.093299
53. H	5.295331	1.523445	0.144492
54. H	5.109776	–0.201157	0.569786
55. H	3.711505	0.908574	0.669669
56. C	5.625517	0.112777	–2.146419
57. H	5.466530	–0.097953	–3.216215
58. H	6.217776	–0.700756	–1.698033
59. H	6.229655	1.030460	–2.080341
60. C	3.549187	1.533118	–2.068465
61. H	3.276763	1.280792	–3.105735
62. H	4.211316	2.412654	–2.088534
63. H	2.637101	1.813074	–1.523931
64. C	3.389569	–4.543678	–2.910140
65. C	4.745349	–4.370750	–3.636103
66. H	4.990524	–5.301033	–4.171625
67. H	5.568377	–4.175575	–2.929728
68. H	4.706124	–3.561422	–4.383048
69. C	3.516376	–5.713902	–1.891805
70. H	2.556938	–5.933386	–1.396608
71. H	4.258192	–5.470531	–1.114518
72. H	3.841803	–6.628698	–2.410618
73. C	2.302428	–4.878165	–3.973845
74. H	1.327102	–5.103737	–3.515539
75. H	2.608710	–5.762276	–4.552773
76. H	2.168634	–4.034517	–4.668119

Cartesian coordinates [Å] for the optimized computed geometry of 7b.

**Atom**	***x***	***y***	***z***
1. As	–0.114184	0.171201	0.265606
2. N	–0.008239	–1.907524	–0.042620
3. C	–0.068760	–2.713413	1.226688
4. H	0.727599	–2.360586	1.895988

5. H	0.117446	–3.770421	0.995772
6. H	–1.059525	–2.593244	1.683084
7. O	–0.712333	0.183752	–1.460031
8. O	1.663314	0.190769	0.038333
9. C	–1.412011	–0.996780	–1.773475
10. C	–2.346281	–1.074521	–2.832088
11. C	–2.907601	–2.348142	–3.058585
12. H	–3.621234	–2.446569	–3.871704
13. C	–2.614262	–3.502662	–2.296017
14. C	–1.694638	–3.379629	–1.238085
15. H	–1.446755	–4.232817	–0.611695
16. C	–1.108721	–2.132271	–1.003125
17. C	–2.702735	0.153019	–3.691711
18. C	–3.281568	1.270529	–2.776366
19. H	–2.534784	1.640606	–2.061244
20. H	–4.153938	0.894918	–2.217856
21. H	–3.608466	2.122006	–3.393529
22. C	–3.767688	–0.189837	–4.763193
23. H	–4.712135	–0.530318	–4.308735
24. H	–3.407445	–0.951019	–5.473716
25. H	–3.993161	0.717007	–5.345439
26. C	–1.418837	0.644827	–4.423080
27. H	–0.642366	0.972962	–3.717608
28. H	–1.669097	1.494728	–5.077491
29. H	–1.004566	–0.163778	–5.046361
30. C	–3.301524	–4.838102	–2.645833
31. C	–2.824252	–5.997217	–1.737294
32. H	–1.740762	–6.176902	–1.838193
33. H	–3.336136	–6.925307	–2.035404
34. H	–3.070854	–5.817999	–0.677613
35. C	–4.840177	–4.675834	–2.471669
36. H	–5.252504	–3.917109	–3.154434
37. H	–5.081874	–4.375530	–1.439327
38. H	–5.345904	–5.630673	–2.685721
39. C	–2.965909	–5.204039	–4.122343
40. H	–3.358907	–4.459958	–4.831640
41. H	–3.415400	–6.176766	–4.377706
42. H	–1.876064	–5.273474	–4.266865
43. C	2.124634	–0.904757	–0.721472
44. C	3.360030	–0.897328	–1.400501
45. C	3.695982	–2.090195	–2.083013
46. H	4.636112	–2.111438	–2.623970
47. C	2.874007	–3.235825	–2.123590
48. C	1.651254	–3.199545	–1.424265
49. H	0.959295	–4.038447	–1.466401
50. C	1.299366	–2.042102	–0.738599
51. C	4.268588	0.347145	–1.409161
52. C	4.704896	0.667744	0.051443
53. H	5.383247	1.535120	0.052299
54. H	5.240804	–0.191571	0.484766

55. H	3.847788	0.907457	0.696227
56. C	5.547222	0.116884	–2.254126
57. H	5.311332	–0.080713	–3.312043
58. H	6.162073	–0.706162	–1.856618
59. H	6.162923	1.028352	–2.220967
60. C	3.488969	1.543396	–2.031041
61. H	3.132182	1.284115	–3.040721
62. H	4.155585	2.416312	–2.112194
63. H	2.625345	1.835428	–1.418240
64. C	3.219171	–4.492150	–2.946517
65. C	4.607394	–4.386018	–3.622007
66. H	4.808609	–5.311787	–4.182778
67. H	5.413647	–4.268391	–2.880453
68. H	4.651254	–3.552790	–4.341789
69. C	3.220915	–5.733584	–2.007410
70. H	2.228732	–5.921910	–1.566846
71. H	3.943394	–5.595065	–1.187180
72. H	3.506660	–6.632823	–2.575179
73. C	2.138583	–4.662087	–4.057550
74. H	1.133126	–4.814701	–3.633709
75. H	2.377932	–5.536802	–4.682661
76. H	2.105437	–3.768697	–4.702045

Cartesian coordinates [Å] for the optimized computed geometry of 8a.

**Atom**	***x***	***y***	***z***
1. P	0.052630	0.142926	0.174566
2. O	1.306267	0.467865	–0.941565
3. O	–1.421222	–0.435469	0.817850
4. N	0.691141	–1.481586	0.330429
5. C	2.334748	–0.473557	–0.928520
6. C	3.580233	–0.331818	–1.573262
7. C	4.458772	–1.435200	–1.456433
8. H	5.429171	–1.371538	–1.935487
9. C	4.141768	–2.618445	–0.742454
10. C	2.886702	–2.715089	–0.105218
11. H	2.621355	–3.601058	0.449756
12. C	1.986144	–1.636734	–0.199909
13. C	3.942300	0.961495	–2.346032
14. C	2.927544	1.161934	–3.524390
15. H	3.201643	2.058156	–4.104267
16. H	1.901727	1.288648	–3.154197
17. H	2.951749	0.287971	–4.195212
18. C	5.378188	0.897586	–2.952903
19. H	5.586447	1.841357	–3.480099
20. H	5.475720	0.078558	–3.682738
21. H	6.144212	0.775599	–2.171525
22. C	5.182450	–3.774882	–0.689429
23. C	6.518682	–3.245489	–0.065283
24. H	7.245339	–4.068713	0.028526
25. H	6.332115	–2.822967	0.934534

26. H	6.975504	–2.461055	–0.685679
27. C	5.444955	–4.293339	–2.145398
28. H	4.504955	–4.648457	–2.596280
29. H	6.166517	–5.126291	–2.128055
30. H	5.853471	–3.500543	–2.789125
31. C	–1.317719	–1.679099	1.440184
32. C	–2.309302	–2.276544	2.244879
33. C	–1.994661	–3.561847	2.749144
34. H	–2.725116	–4.057380	3.379144
35. C	–0.776310	–4.235451	2.477837
36. C	0.187970	–3.599398	1.667242
37. H	1.124391	–4.087039	1.446554
38. C	–0.087240	–2.318705	1.152310
39. C	–3.648481	–1.552847	2.539057
40. C	–4.407217	–1.309876	1.188342
41. H	–3.828179	–0.668936	0.510826
42. H	–5.375501	–0.822289	1.385928
43. H	–4.595614	–2.271508	0.684724
44. C	–4.577231	–2.395615	3.468279
45. H	–5.509276	–1.836493	3.645044
46. H	–4.109612	–2.583058	4.447926
47. H	–4.848257	–3.359139	3.008890
48. C	–0.525923	–5.642029	3.094267
49. C	0.824357	–6.266799	2.621624
50. H	1.687059	–5.661139	2.939527
51. H	0.851994	–6.387430	1.527389
52. H	0.938516	–7.265705	3.070768
53. C	–1.679619	–6.614039	2.670203
54. H	–1.741354	–6.674486	1.572427
55. H	–2.655621	–6.277767	3.049830
56. H	–1.490692	–7.623167	3.069749
57. H	–0.715179	1.092899	–0.528466
58. Cl	0.778403	1.240529	1.865334
59. C	3.885068	2.179421	–1.360145
60. H	4.174583	3.101594	–1.889964
61. H	2.877665	2.316421	–0.945862
62. H	4.584822	2.020043	–0.523752
63. C	4.692067	–4.979586	0.171171
64. H	3.768276	–5.418838	–0.236335
65. H	5.462823	–5.766408	0.166781
66. H	4.519397	–4.685464	1.217857
67. C	–3.355010	–0.189633	3.255699
68. H	–2.756685	0.478711	2.623284
69. H	–4.302926	0.314981	3.501209
70. H	–2.800470	–0.368553	4.190401
71. C	–0.484628	–5.516688	4.656037
72. H	0.313954	–4.821802	4.959447
73. H	–1.436965	–5.136769	5.054956
74. H	–0.289436	–6.501071	5.111352

Cartesian coordinates [Å] for the optimized computed geometry of 9a.

**Atom**	***x***	***y***	***z***
1. P	–0.061245	0.036164	–0.063862
2. N	–0.375083	–2.403458	0.572652
3. O	1.110420	–0.823854	–0.957067
4. O	0.608216	–0.045939	1.513651
5. O	0.692758	1.471336	–0.443477
6. C	0.714543	–2.031082	–1.522042
7. C	1.069320	–2.374006	–2.844326
8. C	0.681327	–3.659628	–3.295419
9. H	0.961439	–3.951357	–4.302401
10. C	–0.054230	–4.569594	–2.511851
11. C	–0.433018	–4.170288	–1.212336
12. H	–1.017100	–4.833382	–0.573979
13. C	–0.047468	–2.919564	–0.721415
14. C	1.837008	–1.385018	–3.748596
15. C	1.032589	–0.055743	–3.876967
16. H	0.005134	–0.266143	–4.217377
17. H	1.518669	0.599683	–4.619644
18. H	0.989871	0.482671	–2.922289
19. C	3.239629	–1.109704	–3.133389
20. H	3.803852	–2.052864	–3.046826
21. H	3.156614	–0.659318	–2.134975
22. H	3.803986	–0.419271	–3.783039
23. C	2.038125	–1.952275	–5.177791
24. H	2.640398	–2.875398	–5.173053
25. H	2.579179	–1.207816	–5.784462
26. H	1.073712	–2.151911	–5.674169
27. C	–0.436811	–5.981054	–3.009607
28. C	–1.985311	–6.130587	–2.999822
29. H	–2.398845	–6.017228	–1.985342
30. H	–2.275595	–7.126341	–3.375506
31. H	–2.444674	–5.362071	–3.642346
32. C	0.203532	–7.038359	–2.062559
33. H	–0.187007	–6.952998	–1.036373
34. H	1.296223	–6.900162	–2.023754
35. H	–0.012433	–8.057767	–2.424795
36. C	0.067223	–6.255075	–4.448333
37. H	–0.229693	–7.273165	–4.750698
38. H	1.166044	–6.195807	–4.510145
39. H	–0.372709	–5.550531	–5.172578
40. C	1.168921	–1.209752	2.002756
41. C	2.201729	–1.159353	2.979794
42. C	2.767333	–2.388791	3.365715
43. H	3.566819	–2.375729	4.102768
44. C	2.378304	–3.641443	2.829967
45. C	1.335080	–3.651889	1.892836
46. H	0.988815	–4.573011	1.425298
47. C	0.723678	–2.450038	1.508415
48. C	2.673823	0.189646	3.562944

49. C	3.779141	–0.000993	4.633349
50. H	4.067017	0.986572	5.029867
51. H	4.684370	–0.464532	4.207263
52. H	3.424456	–0.610525	5.481200
53. C	1.469394	0.900943	4.249730
54. H	1.801894	1.851061	4.701060
55. H	1.059014	0.259072	5.047530
56. H	0.667130	1.117093	3.532283
57. C	3.257079	1.075624	2.422364
58. H	3.622576	2.028428	2.842267
59. H	2.507337	1.292703	1.651121
60. H	4.103116	0.556732	1.941878
61. C	3.110493	–4.923083	3.278706
62. C	2.947520	–5.099560	4.816203
63. H	3.449337	–6.023581	5.149707
64. H	1.878929	–5.161437	5.079312
65. H	3.385440	–4.255411	5.371395
66. C	4.618256	–4.799798	2.914695
67. H	5.155342	–5.722695	3.191217
68. H	5.094443	–3.957983	3.441388
69. H	4.733462	–4.631873	1.831677
70. C	2.546357	–6.187131	2.582330
71. H	3.100450	–7.074868	2.929164
72. H	2.658435	–6.129230	1.487416
73. H	1.482252	–6.340810	2.826866
74. C	–1.669427	–2.794298	1.155652
75. H	–2.457972	–2.633201	0.405766
76. H	–1.854720	–2.147444	2.026714
77. H	–1.687519	–3.847710	1.496177
78. C	–0.077897	2.735853	–0.336846
79. C	0.935465	3.808978	–0.773913
80. H	1.266446	3.614959	–1.805614
81. H	1.816986	3.783493	–0.115743
82. H	0.479483	4.810297	–0.726007
83. C	–1.286877	2.704259	–1.296651
84. H	–0.948934	2.466600	–2.317044
85. H	–1.782068	3.689438	–1.309204
86. H	–2.025295	1.949943	–0.986359
87. C	–0.513973	2.958428	1.126249
88. H	0.361174	2.904783	1.790096
89. H	–1.240749	2.194651	1.446114
90. H	–0.984992	3.949355	1.235539

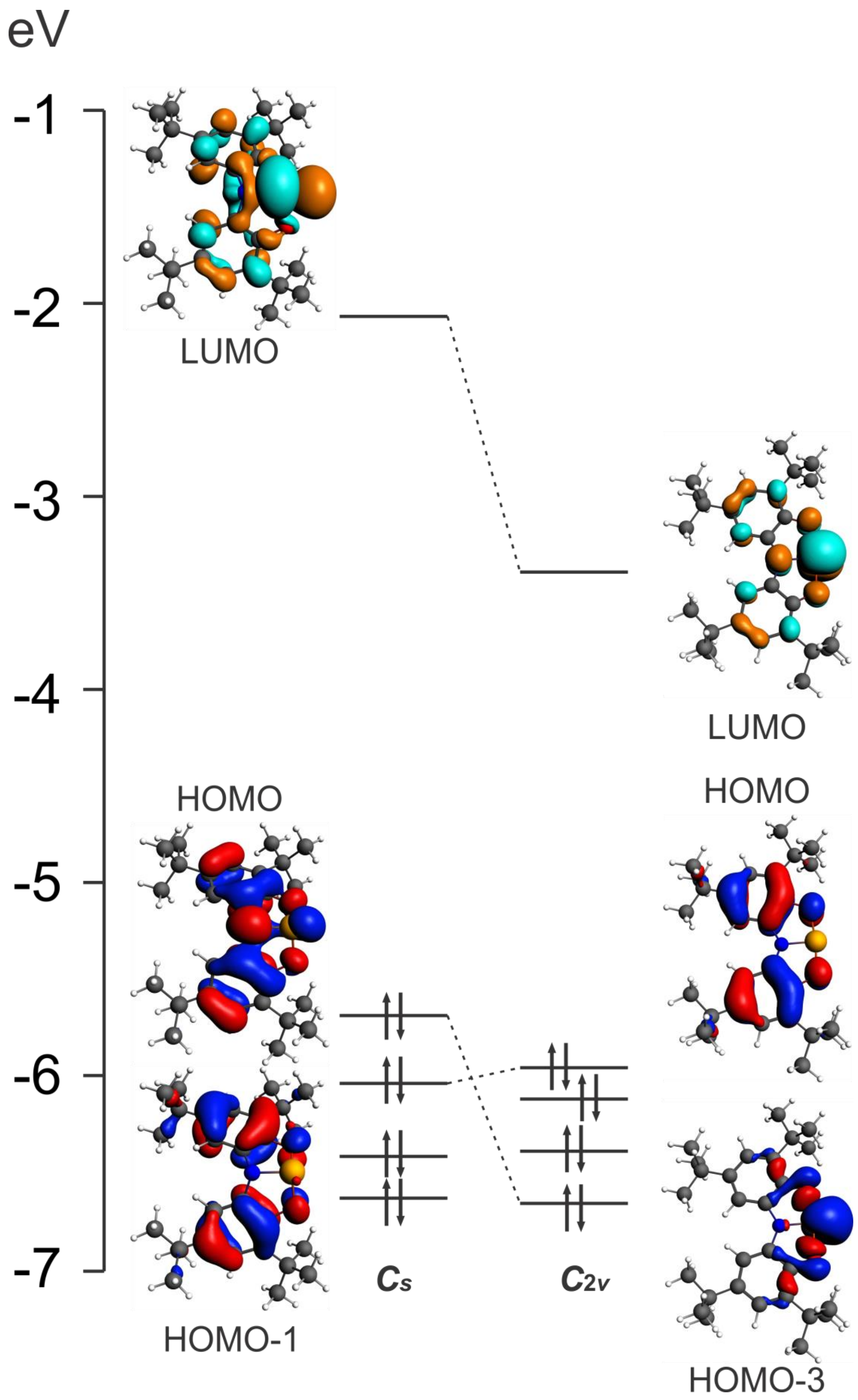

Figure S1. Relative frontier orbital energies and selected Kohn-Sham molecular orbital representations (contour values 0.03 au) for selected orbitals of **1a** in pyramidal (left) and planar (right) configurations.

2. NMR data

NMR spectra for 1b

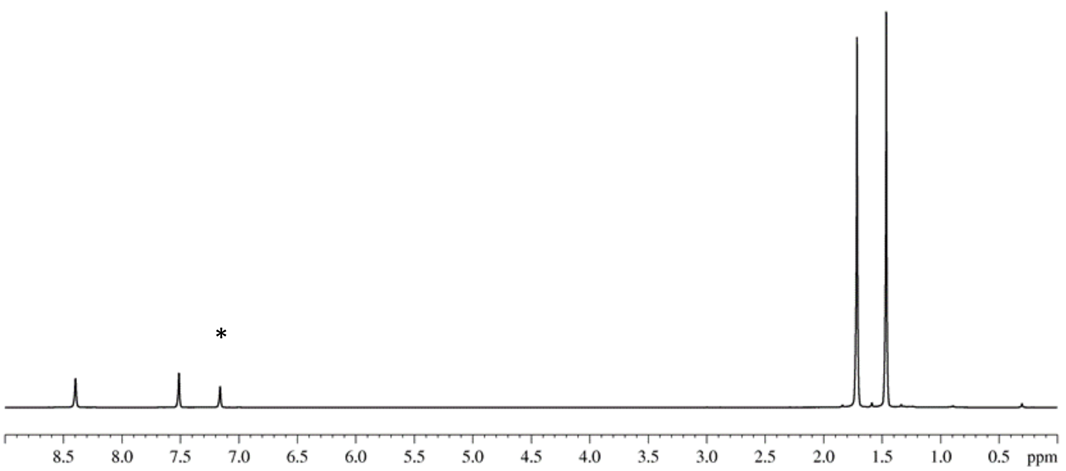

Figure S2: ^{1}H NMR spectrum of **1b** in C_6D_6 at room temperature (* denotes NMR solvent residue).

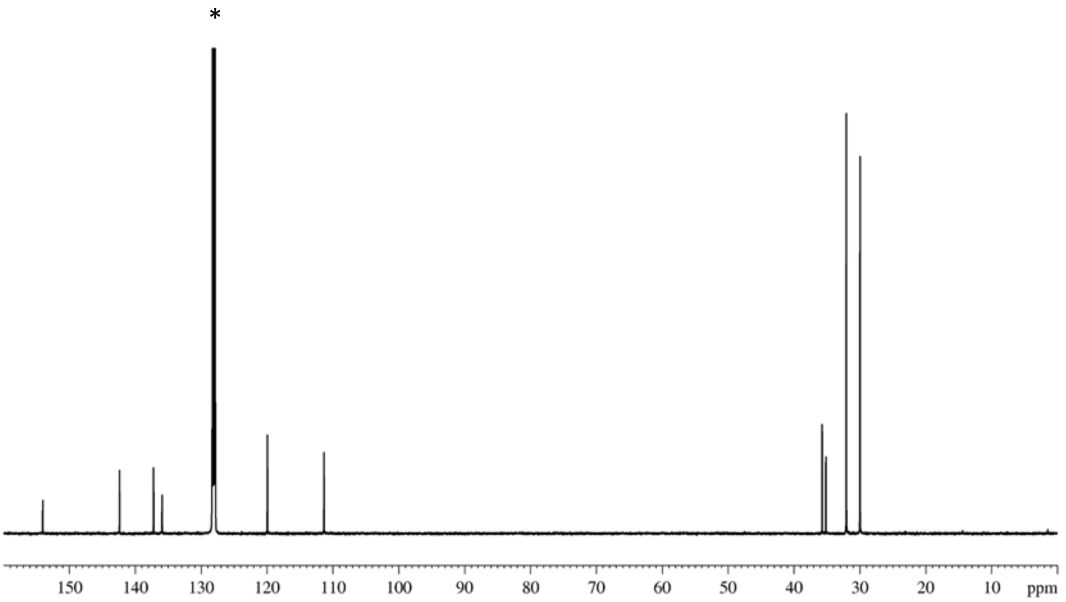

Figure S3: $^{13}C\{^{1}H\}$ NMR spectrum of **1b** in C_6D_6 at room temperature (* denotes NMR solvent residue).

NMR spectra for [K(18-crown-6)][2a]

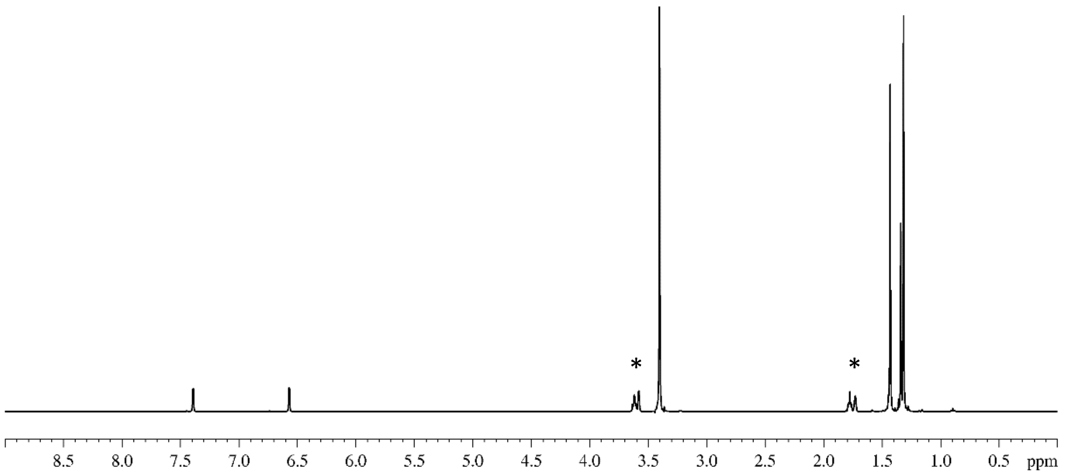

Figure S4: ^{1}H NMR spectrum of [K(18-crown-6)][**2a**] in d_8-THF at room temperature (* denotes residual THF and NMR solvent residue).

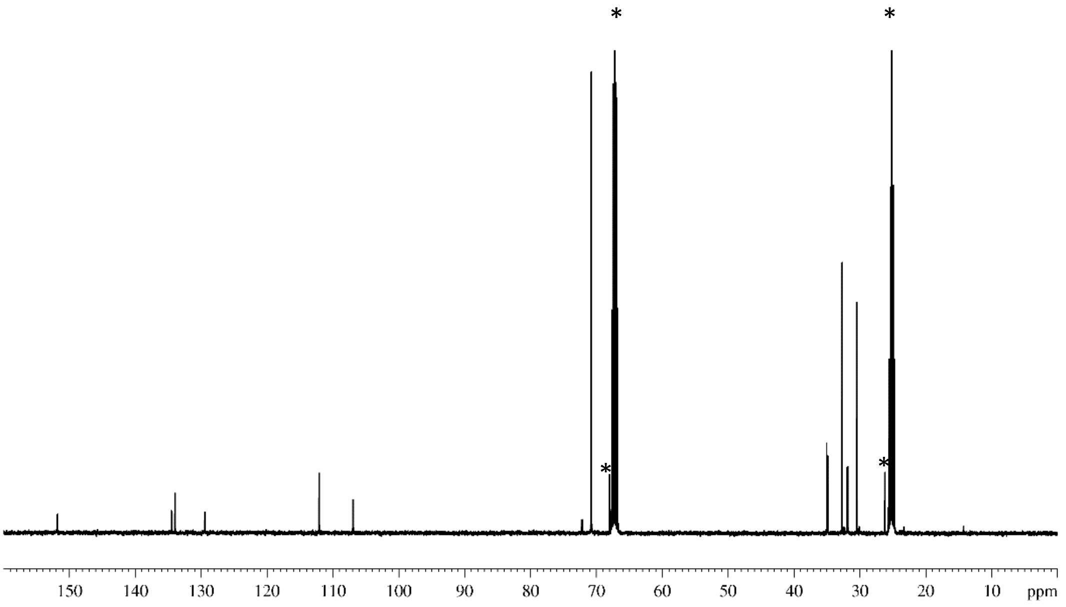

Figure S5: $^{13}C\{^{1}H\}$ NMR spectrum of [K(18-crown-6)][**2a**] in d_8-THF at room temperature (* denotes residual THF and NMR solvent residue).

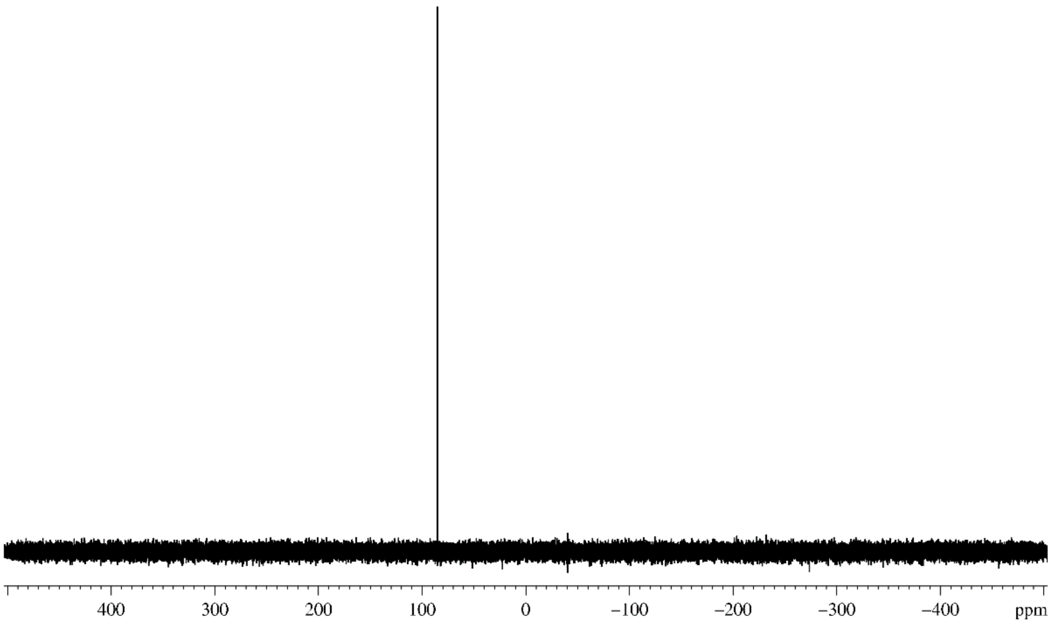

Figure S6: ^{31}P NMR spectrum of [K(18-crown-6)][**2a**] in d_8-THF at room temperature.

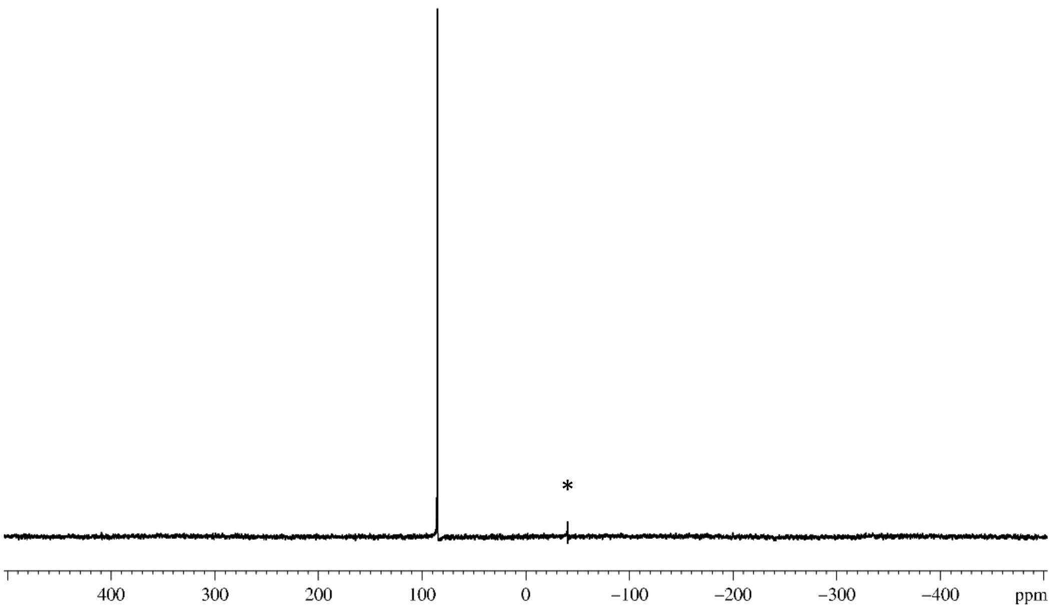

Figure S7: $^{31}P\{^1H\}$ NMR spectrum of [K(18-crown-6)][**2a**] in d_8-THF at room temperature (* Denotes trace amount of (^{t}BuO)HP(ONO)).

NMR spectra for [K(18-crown-6)][2b]

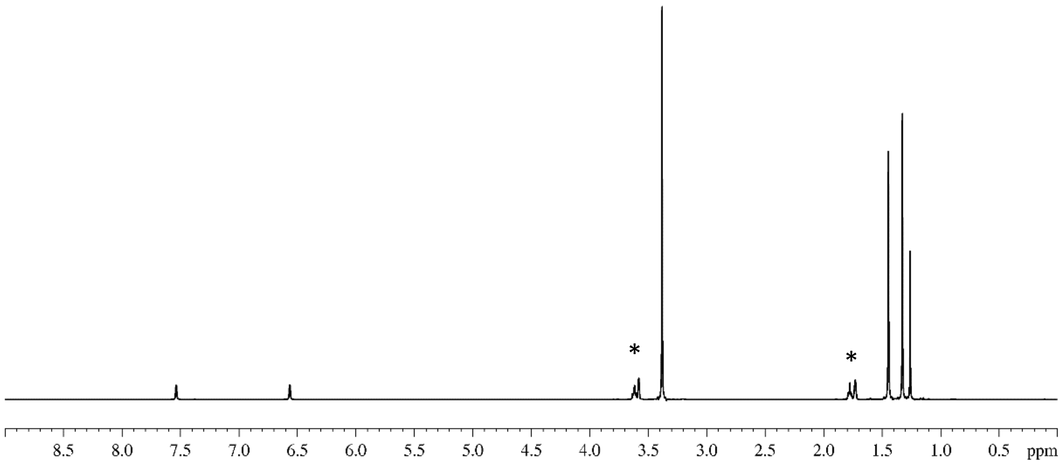

Figure S8: ^{1}H NMR spectrum of [K(18-crown-6)][**2b**] in d_8-THF at room temperature (* denotes residual THF and NMR solvent residue).

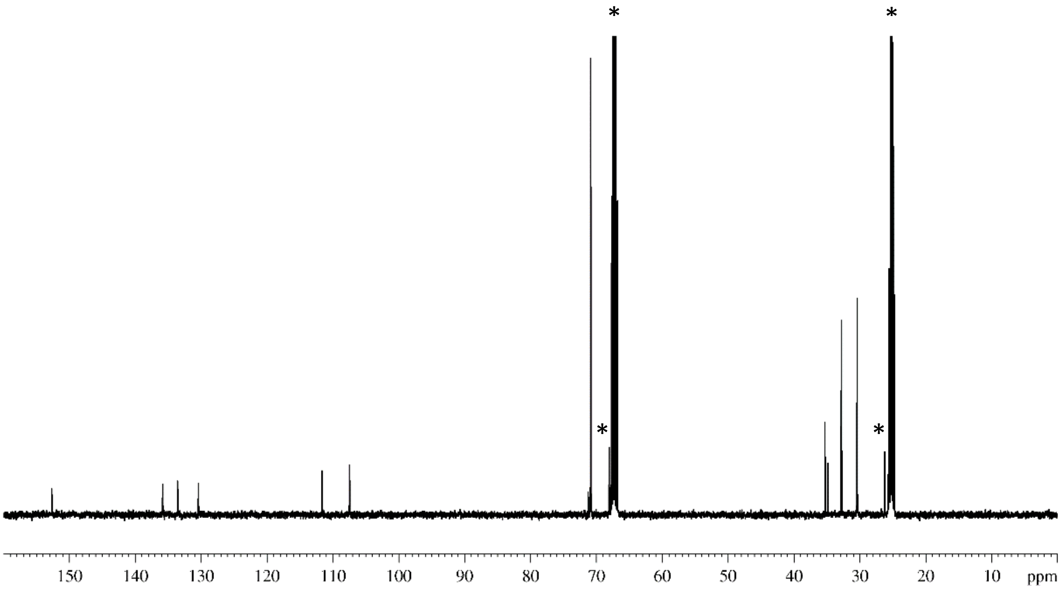

Figure S9: $^{13}C\{^{1}H\}$ NMR spectrum of [K(18-crown-6)][**2b]** in d_8-THF at room temperature (* denotes residual THF and NMR solvent residue).

NMR spectra for [K(18-crown-6)][3a]

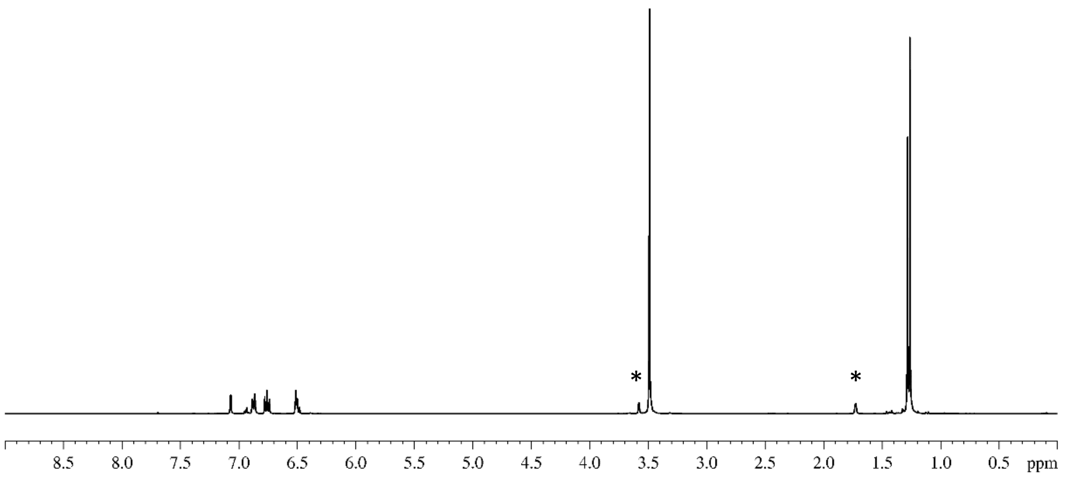

Figure S10: ^{1}H NMR spectrum of *in situ* synthesised [K(18-crown-6)][**3a]** in d_8-THF at room temperature (* denotes NMR solvent residue).

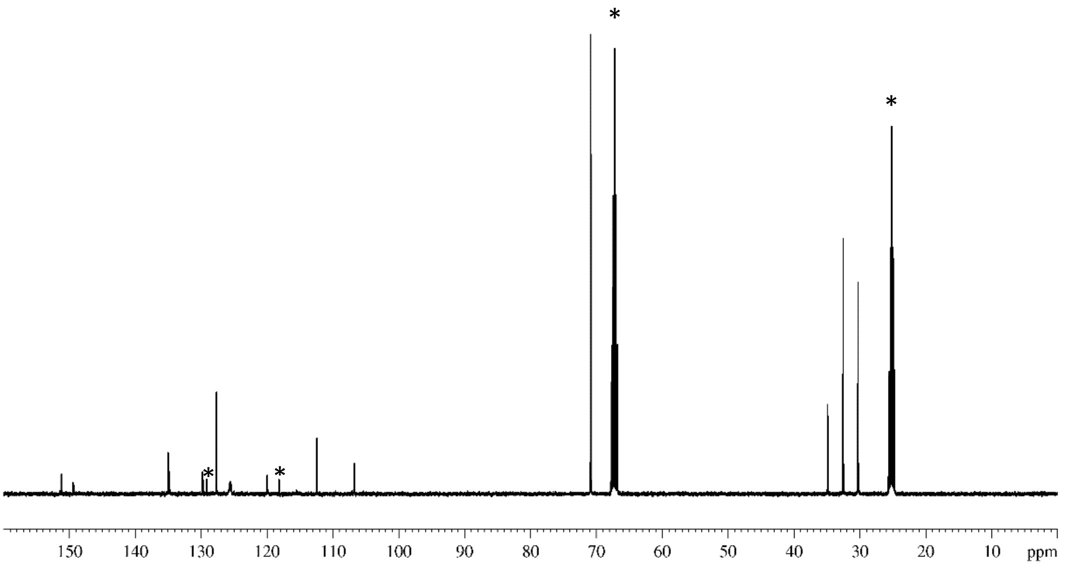

Figure S11: $^{13}C\{^{1}H\}$ NMR spectrum of *in situ* synthesised [K(18-crown-6)][**3a**] in d_8-THF at room temperature (* denotes NMR solvent residue and slight $[NPh_2]^{-}$ impurity).

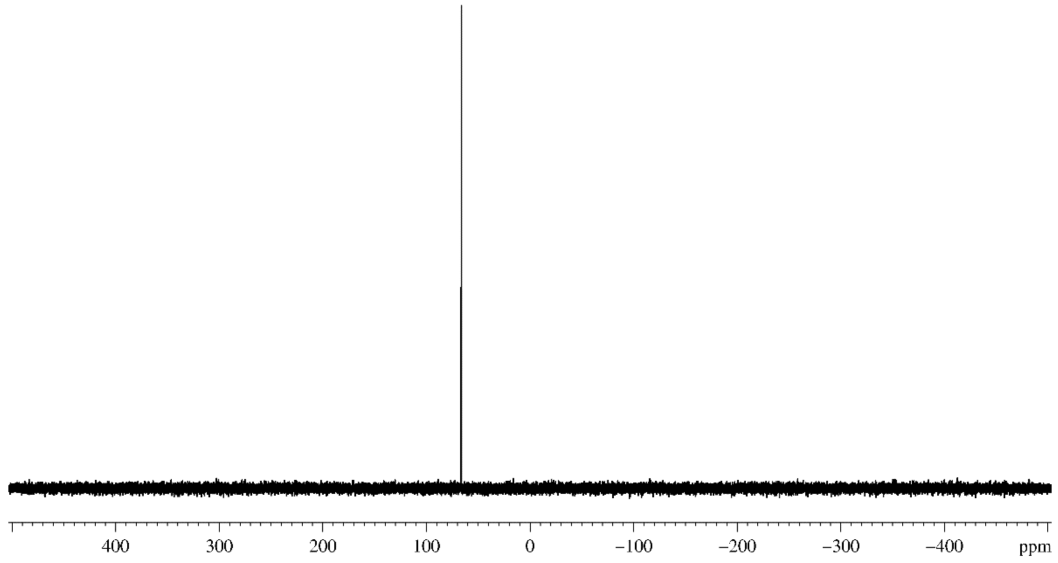

Figure S12: ^{31}P NMR spectrum of *in situ* synthesised [K(18-crown-6)][**3a**] in d_8-THF at room temperature.

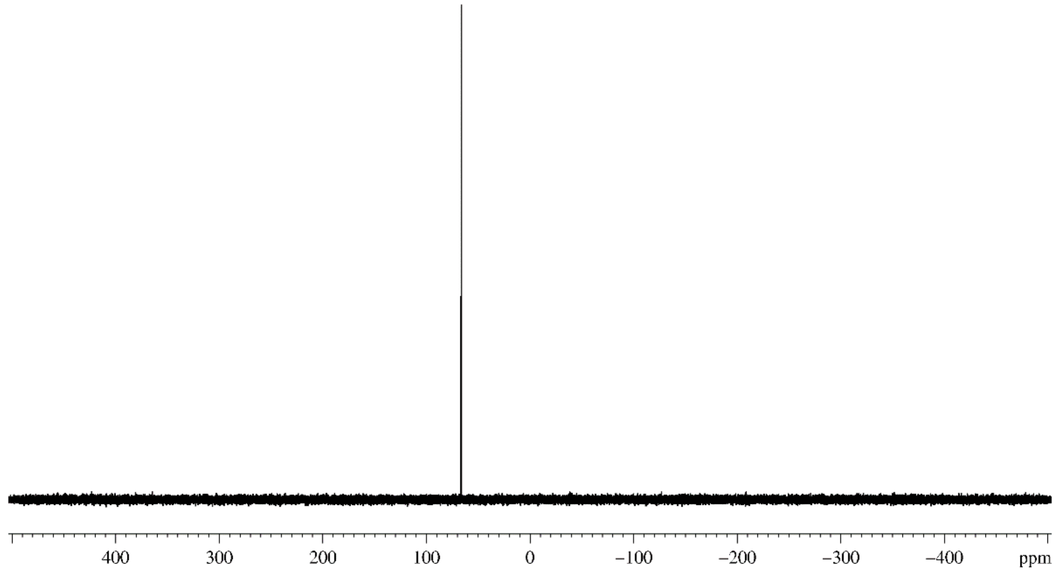

Figure S13: ^{31}P{^{1}H} NMR spectrum of *in situ* synthesised [K(18-crown-6)][**3a**] in d_8-THF at room temperature.

NMR spectra for [K(2,2,2-crypt)][3b]

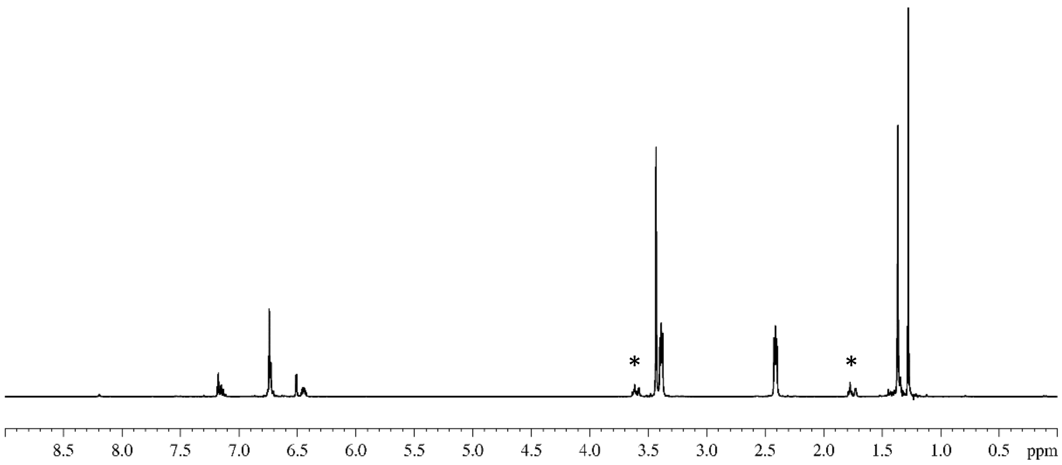

Figure S14: ^{1}H NMR spectrum of [K(2,2,2-crypt)][**3b**] in d_8-THF at room temperature (* denotes residual THF and NMR solvent residue).

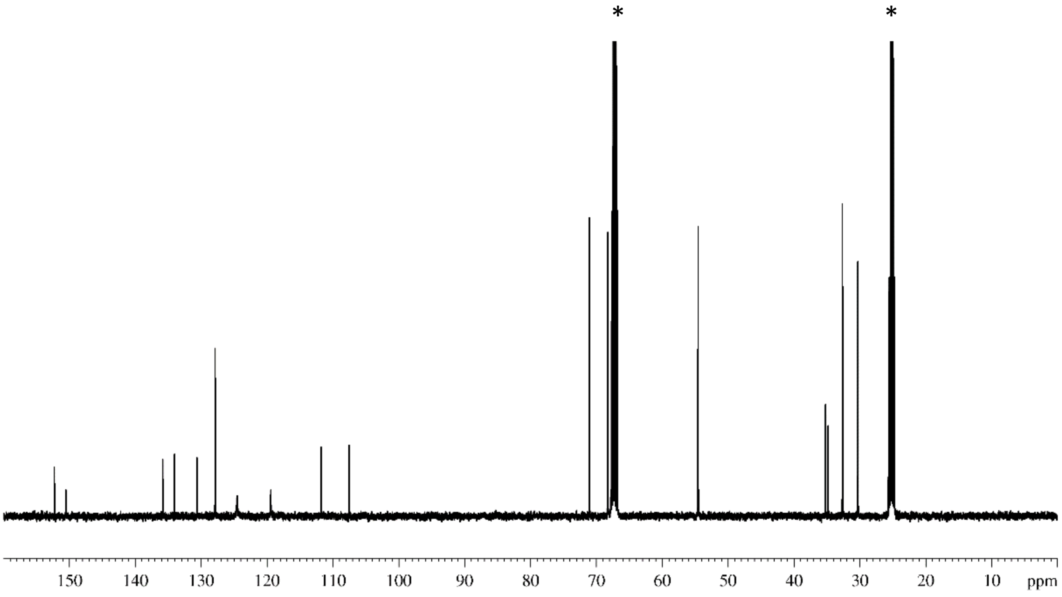

Figure S15: ^{13}C{^{1}H} NMR spectrum of [K(2,2,2-crypt)][**3b**] in d_8-THF at room temperature (* NMR solvent residue).

NMR spectra for 4a

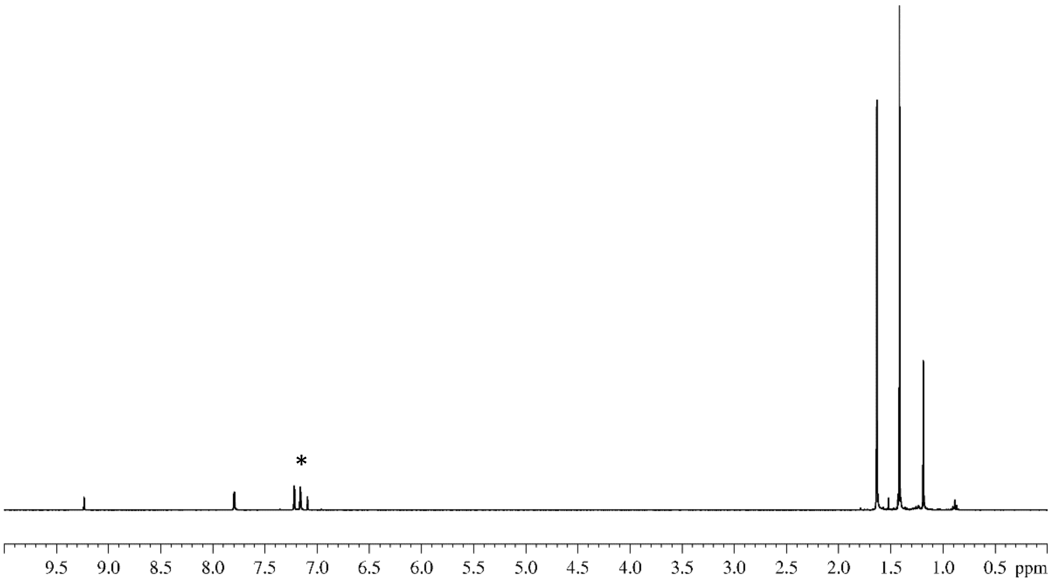

Figure S16: ^{1}H NMR spectrum of **4a** in C_6D_6 at room temperature (* denotes NMR solvent residue).

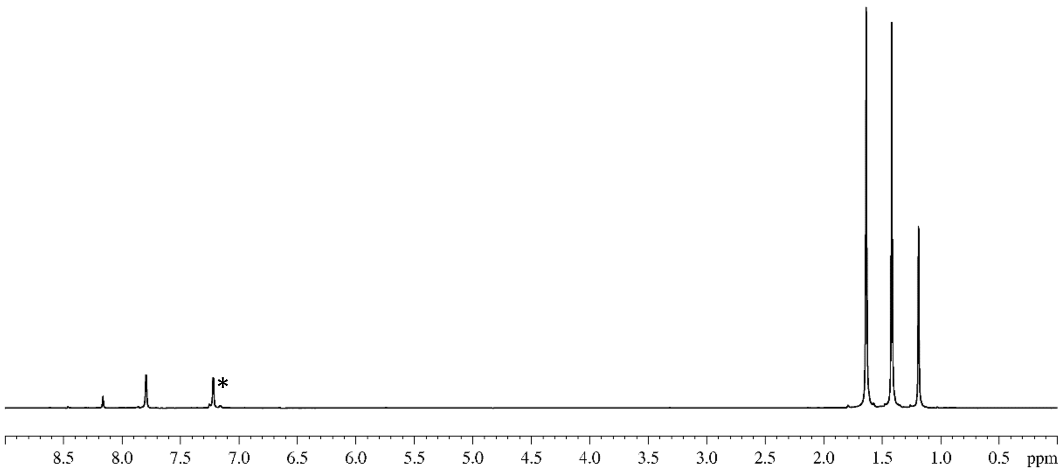

Figure S17: $^{1}H\{^{31}P\}$ NMR spectrum of **4a** in C_6D_6 at room temperature (* denotes NMR solvent residue).

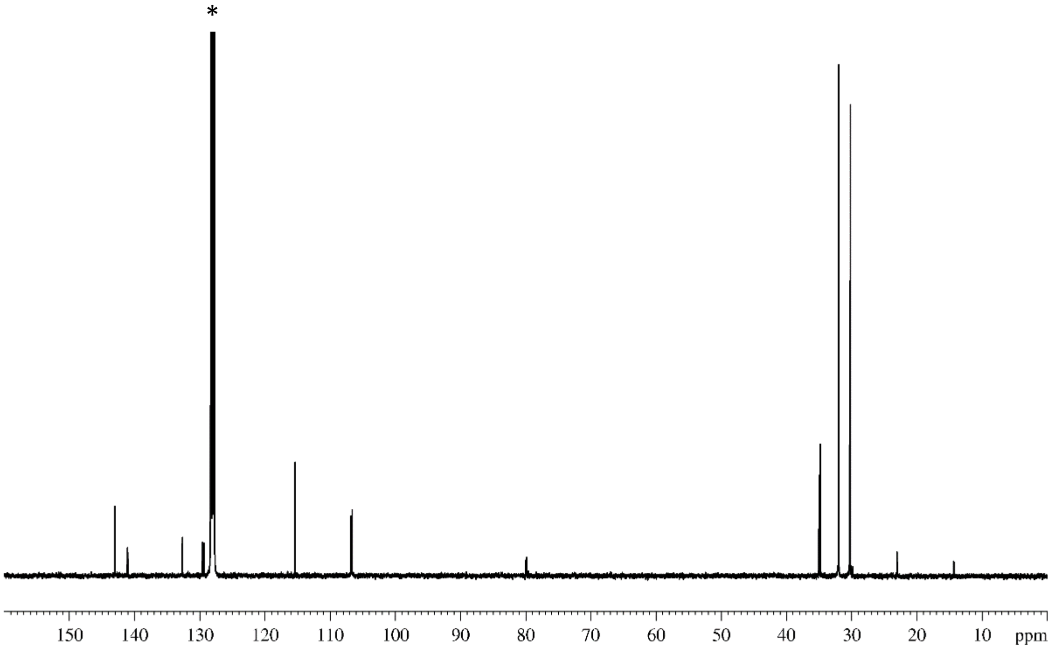

Figure S18: $^{13}C\{^{1}H\}$ NMR spectrum of **4a** in C_6D_6 at room temperature (* denotes NMR solvent residue).

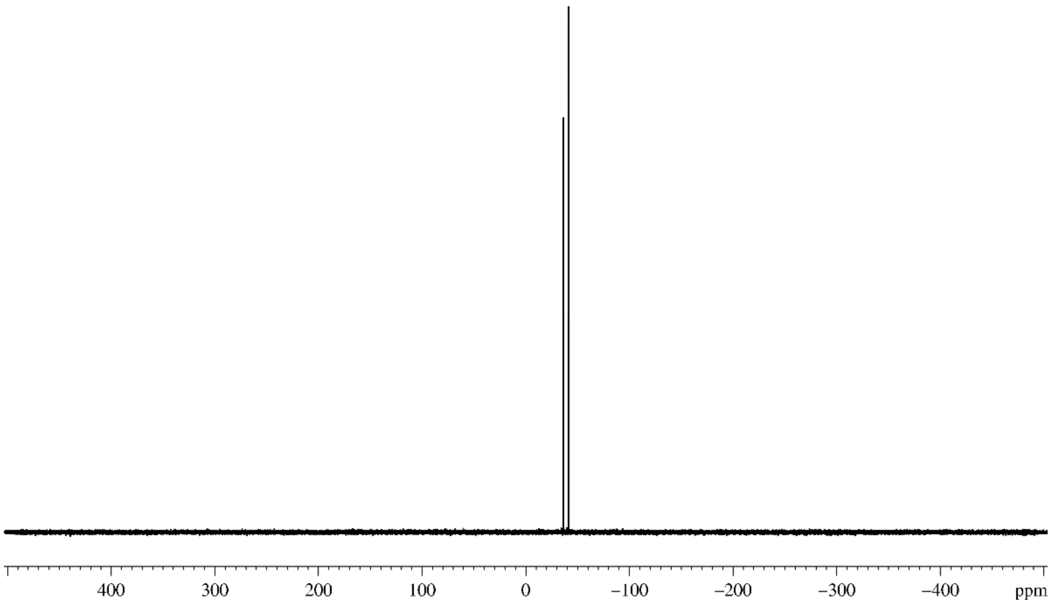

Figure S19: ^{31}P NMR spectrum of **4a** in C_6D_6 at room temperature.

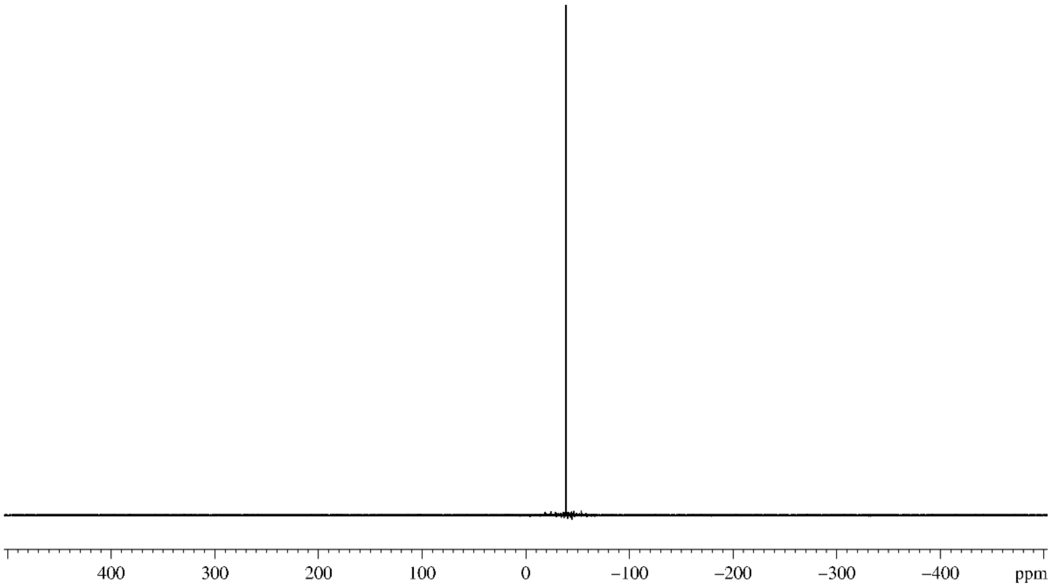

Figure S20: $^{31}P\{^{1}H\}$ NMR spectrum of **4a** in C_6D_6 at room temperature.

NMR spectra for 5a

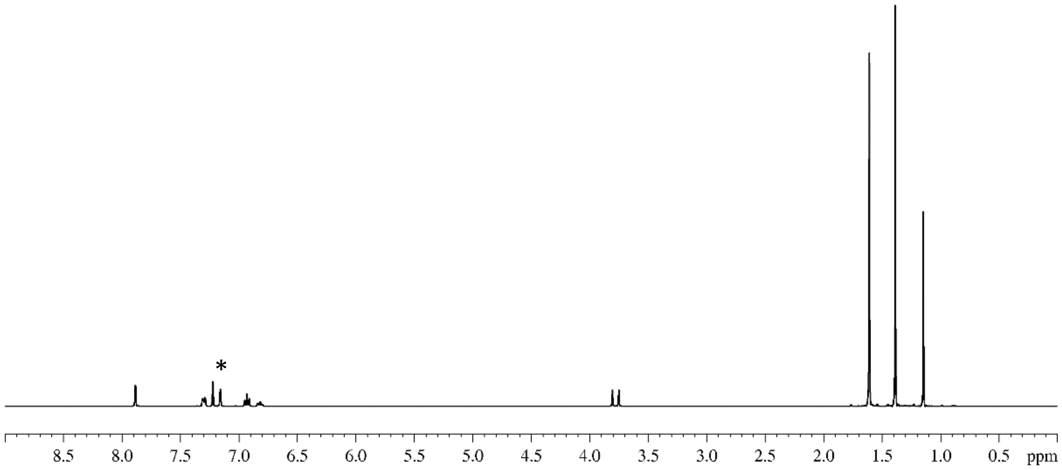

Figure S21: ^{1}H NMR spectrum of **5a** in C_6D_6 at room temperature (* denotes NMR solvent residue).

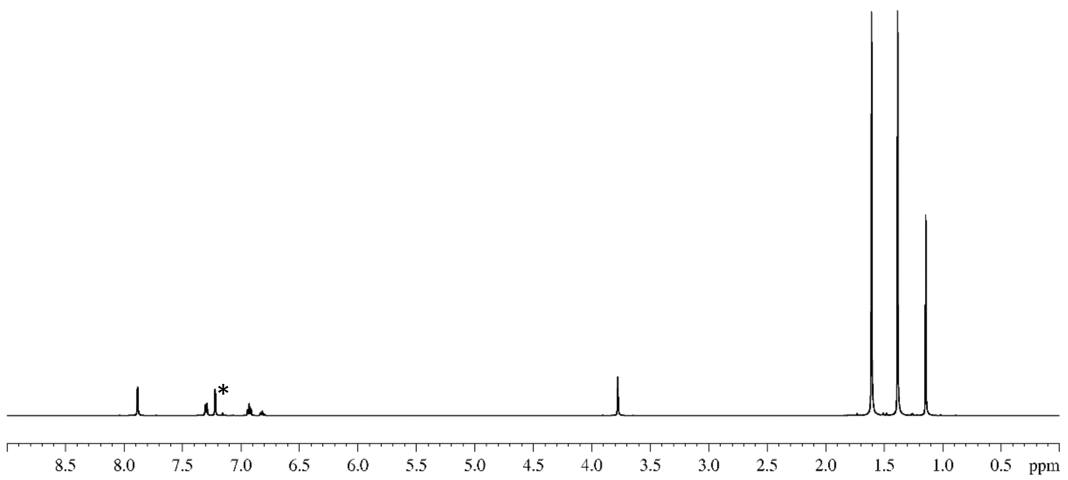

Figure S22: $^{1}H\{^{31}P\}$ NMR spectrum of **5a** in C_6D_6 at room temperature (* denotes NMR solvent residue).

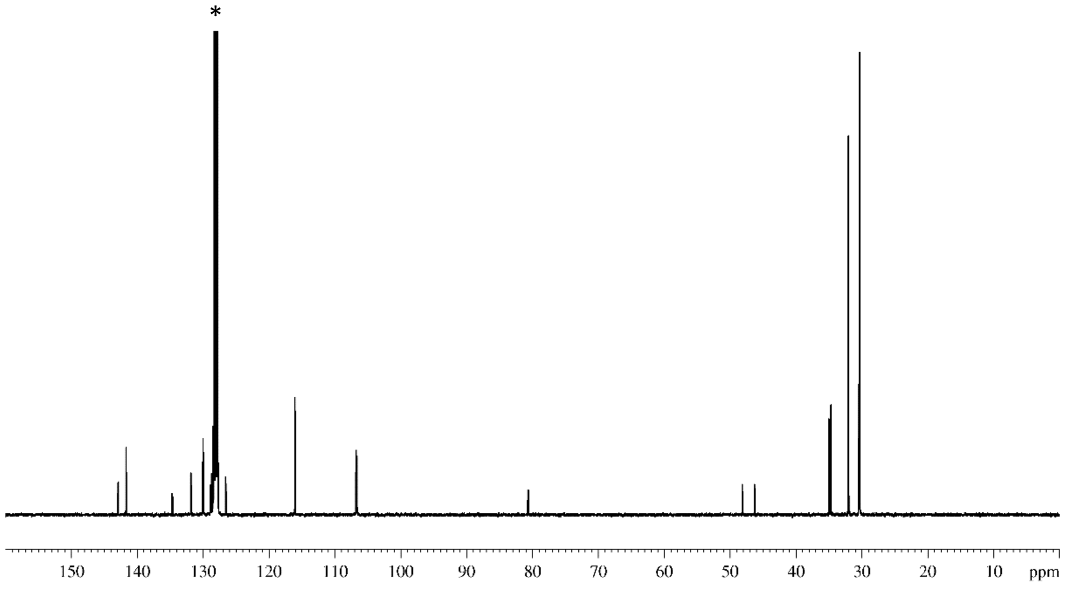

Figure S23: $^{13}C\{^{1}H\}$ NMR spectrum of **5a** in C_6D_6 at room temperature (* denotes NMR solvent residue).

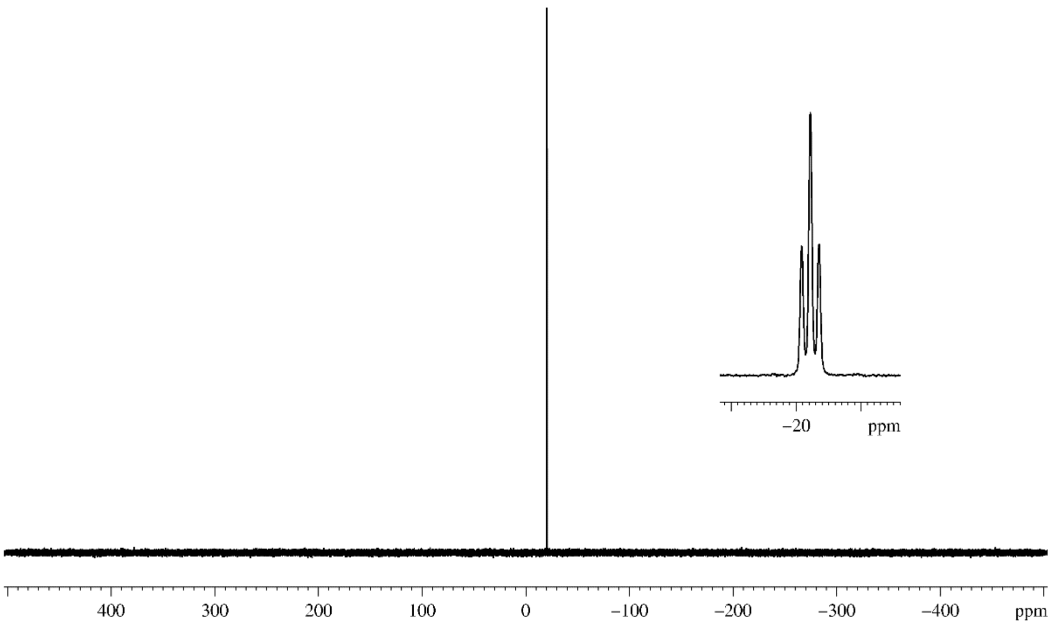

Figure S24: ^{31}P NMR spectrum of **5a** in C_6D_6 at room temperature with inset showing the triplet multiplicity.

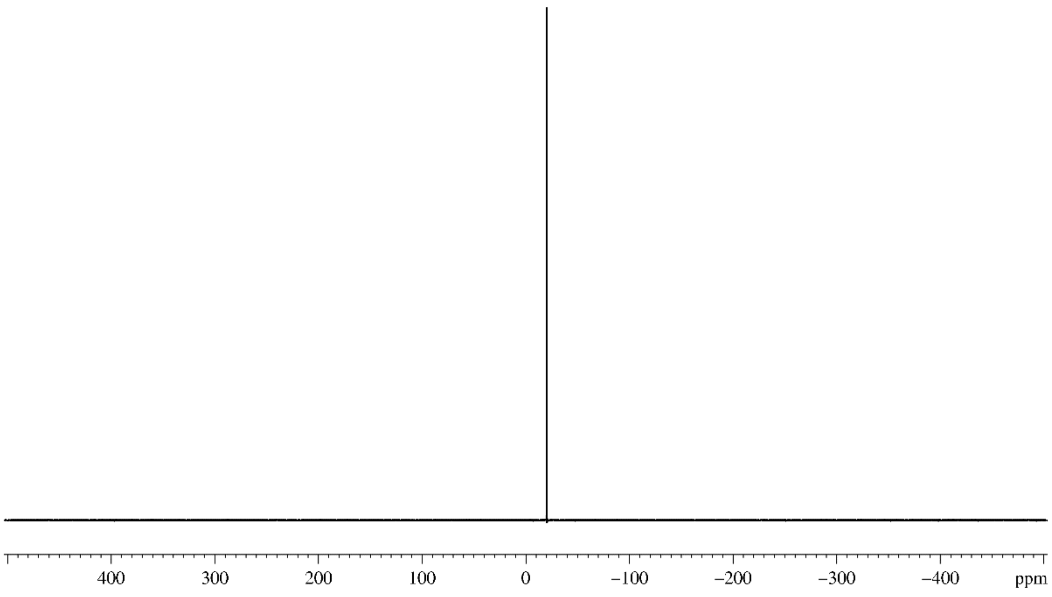

Figure S25: $^{31}P\{^1H\}$ NMR spectrum of **5a** in C_6D_6 at room temperature.

NMR spectra for [6a][OTf]

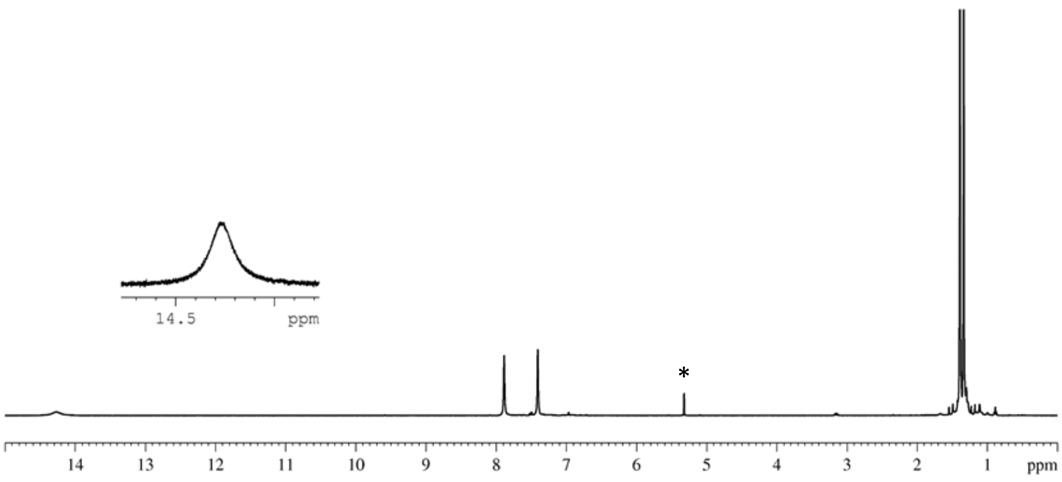

Figure S26: ^{1}H NMR spectrum of [**6a**][OTf] in CD_2Cl_2 at room temperature (* denotes NMR solvent residue).

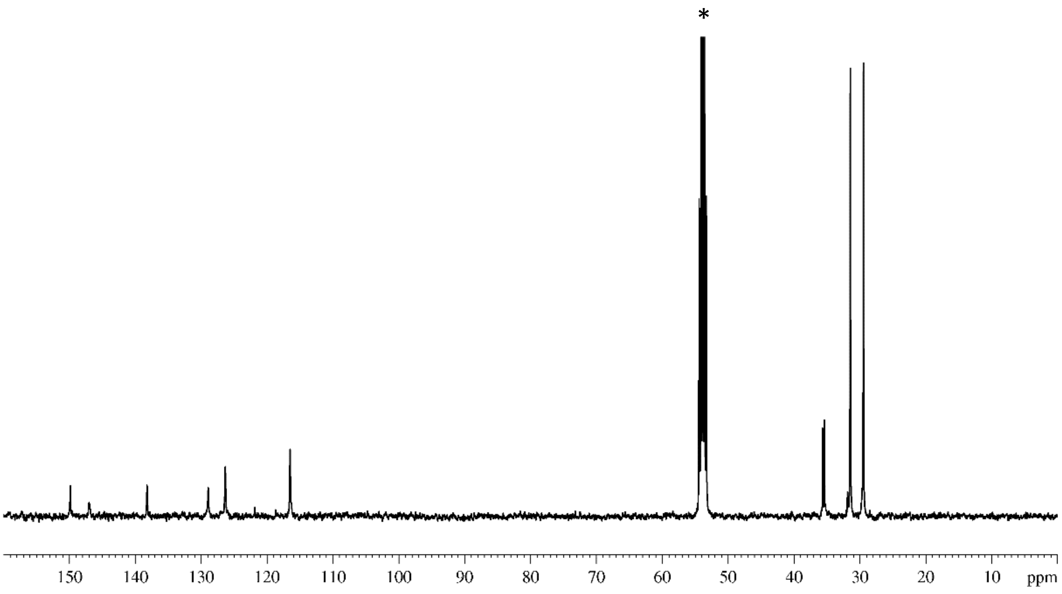

Figure S27: $^{13}C\{^{1}H\}$ NMR spectrum of [**6a**][OTf] in CD_2Cl_2 at room temperature (* denotes NMR solvent residue).

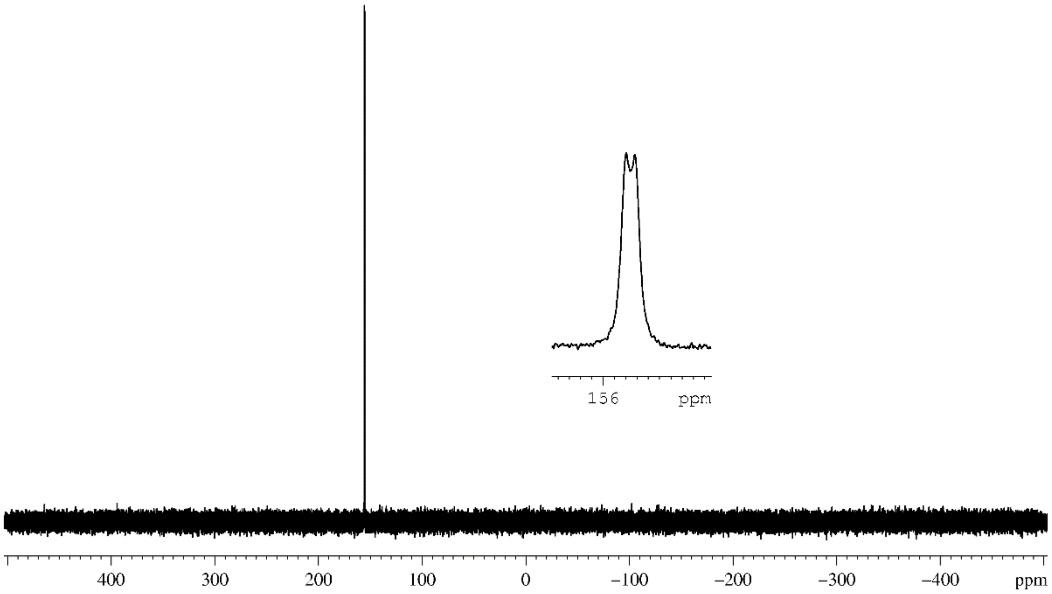

Figure S28: ^{31}P NMR spectrum of [**6a**][OTf] in CD_2Cl_2 at room temperature with inset showing the doublet multiplicity.

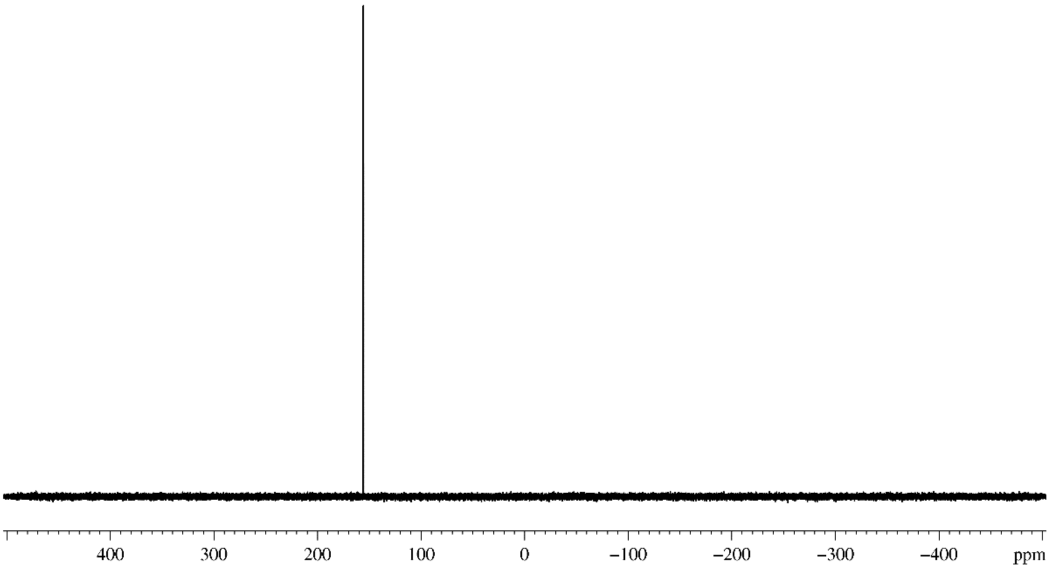

Figure S29: $^{31}P\{^1H\}$ NMR spectrum of [**6a**][OTf] in CD_2Cl_2 at room temperature.

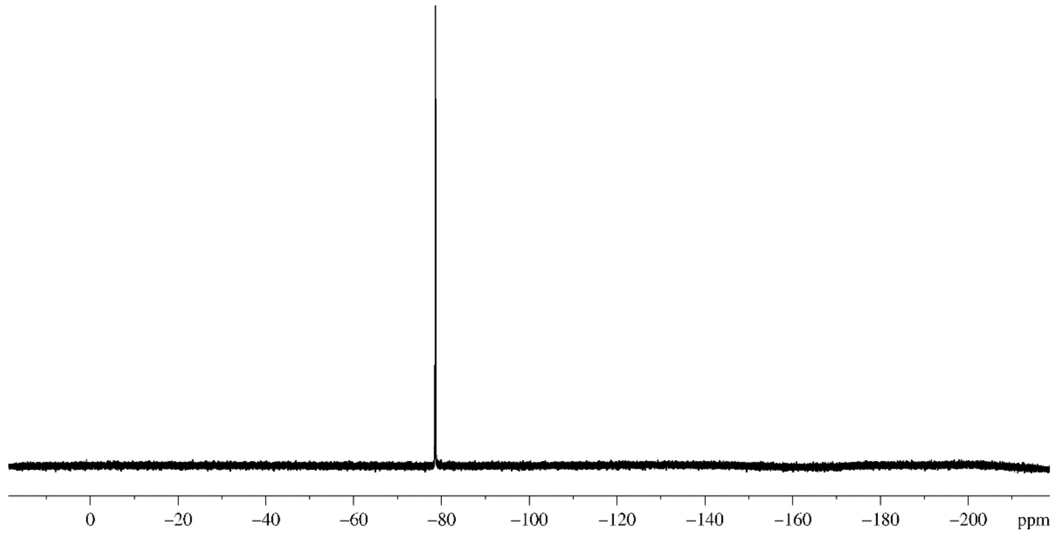

Figure S30: ^{19}F NMR spectrum of **6a** in CD_2Cl_2 at room temperature.

NMR spectra for [6b][OTf]

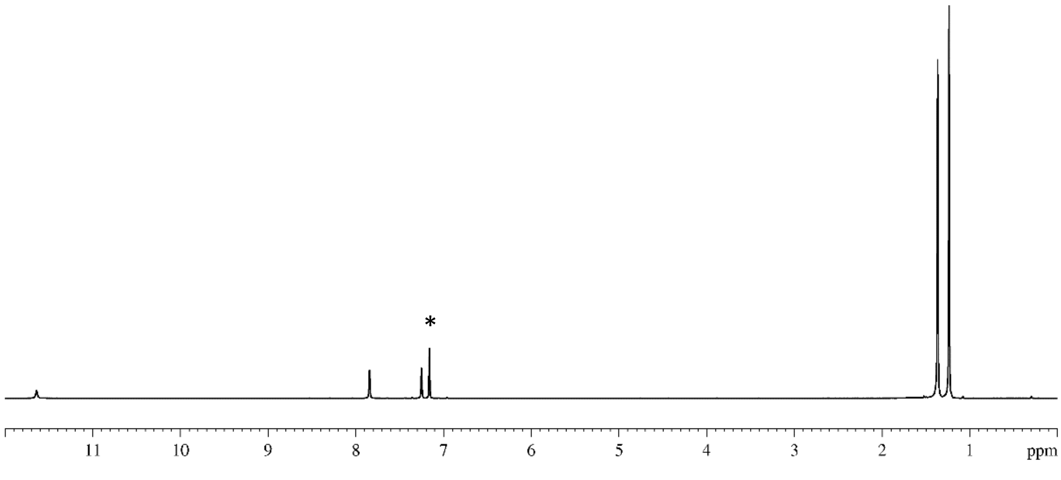

Figure S31: ^{1}H NMR spectrum of [**6b**][OTf] in C_6D_6 at room temperature (* denotes NMR solvent residue).

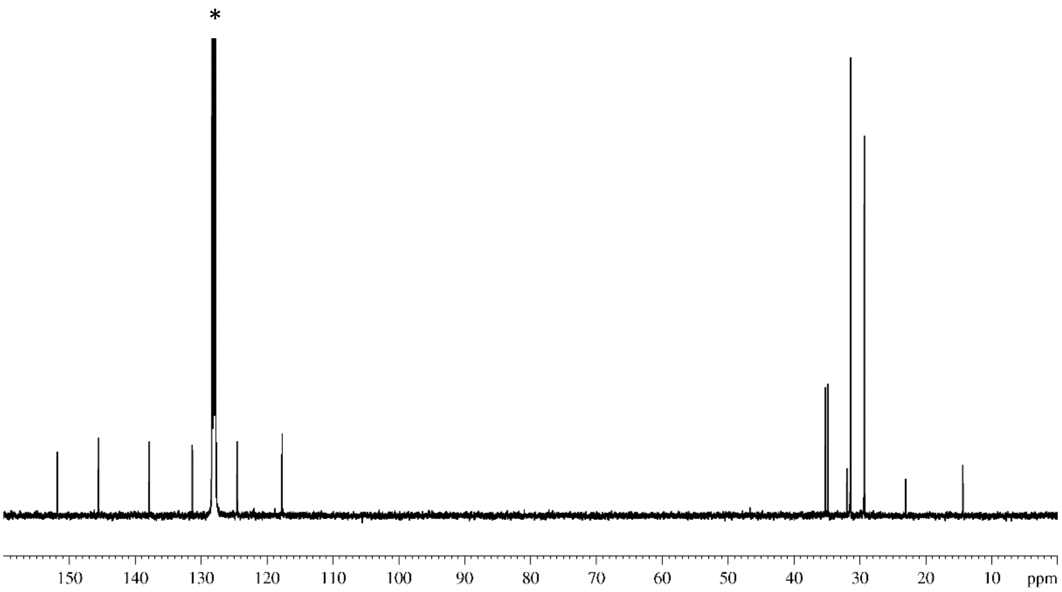

Figure S32: $^{13}C\{^{1}H\}$ NMR spectrum of [**6b**][OTf] in C_6D_6 at room temperature (* denotes NMR solvent residue).

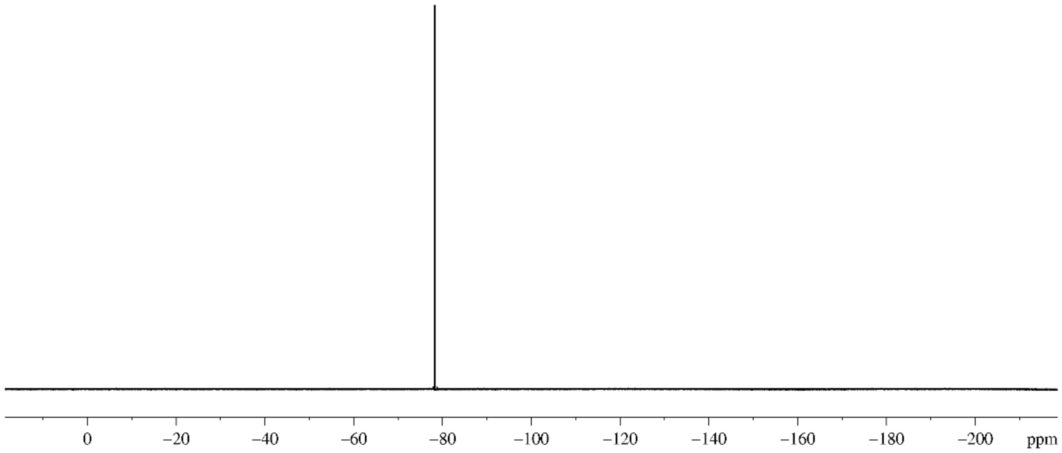

Figure S33: ^{19}F NMR spectrum of [**6b**][OTf] in C_6D_6 at room temperature.

NMR spectra for [7a][OTf]

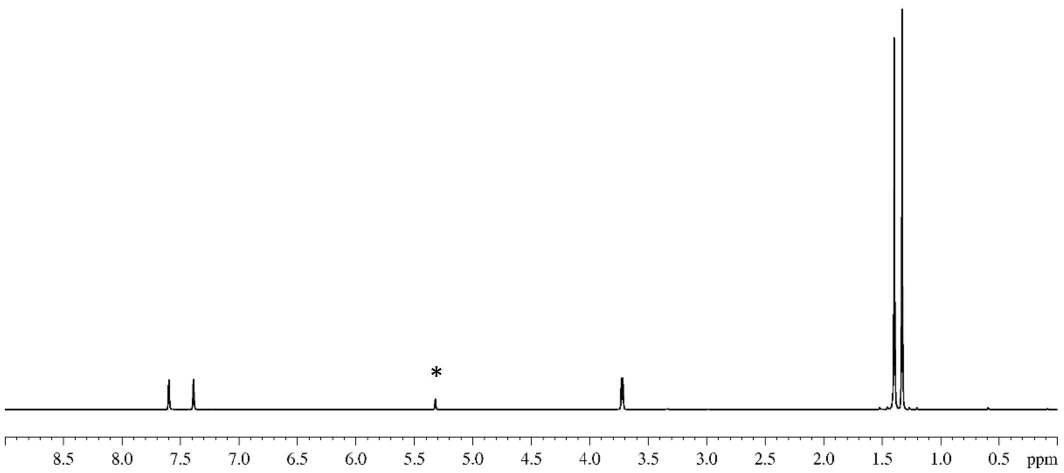

Figure S34: ^{1}H NMR spectrum of [**7a**][OTf] in CD_2Cl_2 at room temperature (* denotes NMR solvent residue).

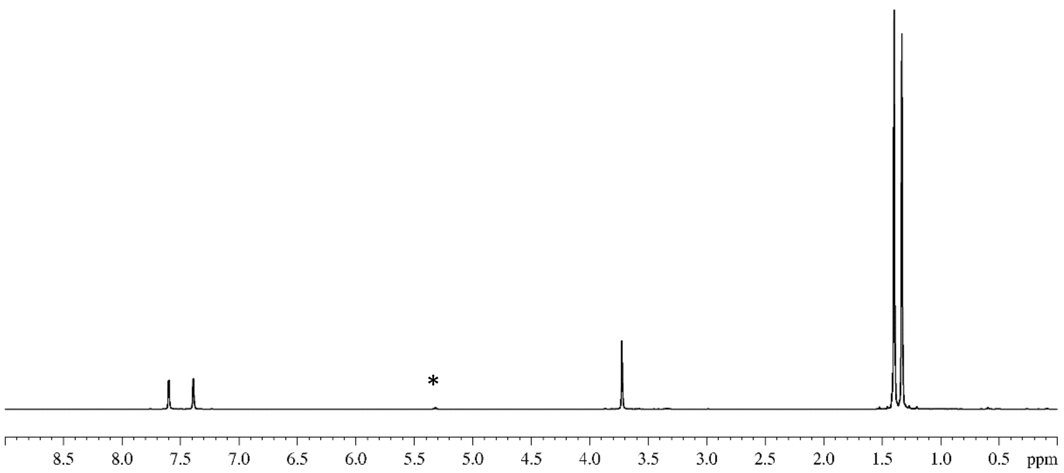

Figure S35: $^{1}H\{^{31}P\}$ NMR spectrum of [**7a**][OTf] in CD_2Cl_2 at room temperature (* denotes NMR solvent residue).

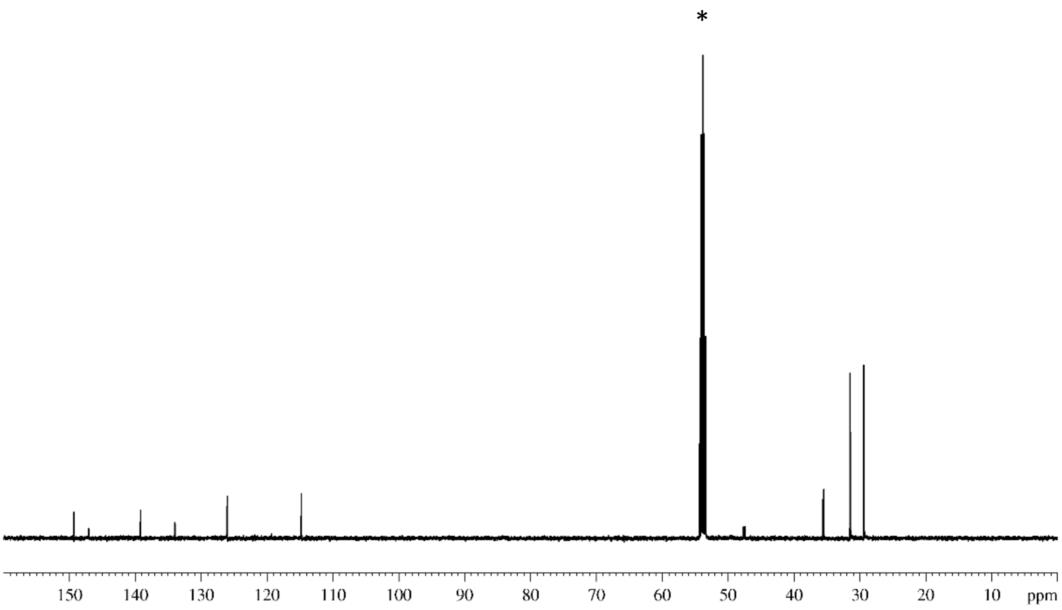

Figure S36: $^{13}C\{^{1}H\}$ NMR spectrum of [**7a**][OTf] in CD_2Cl_2 at room temperature (* denotes NMR solvent residue).

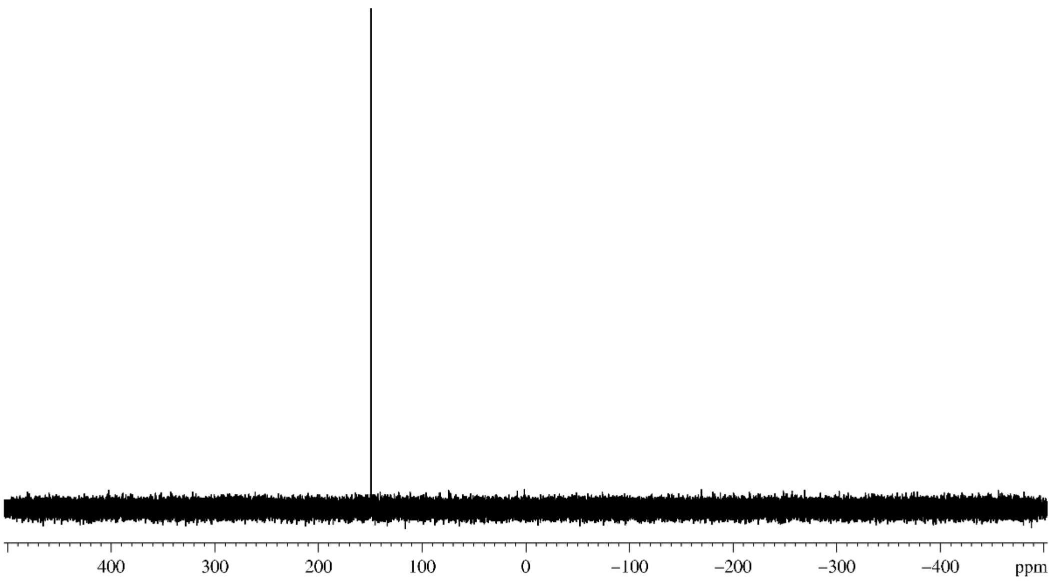

Figure S37: ^{31}P NMR spectrum of [**7a**][OTf] in CD_2Cl_2 at room temperature.

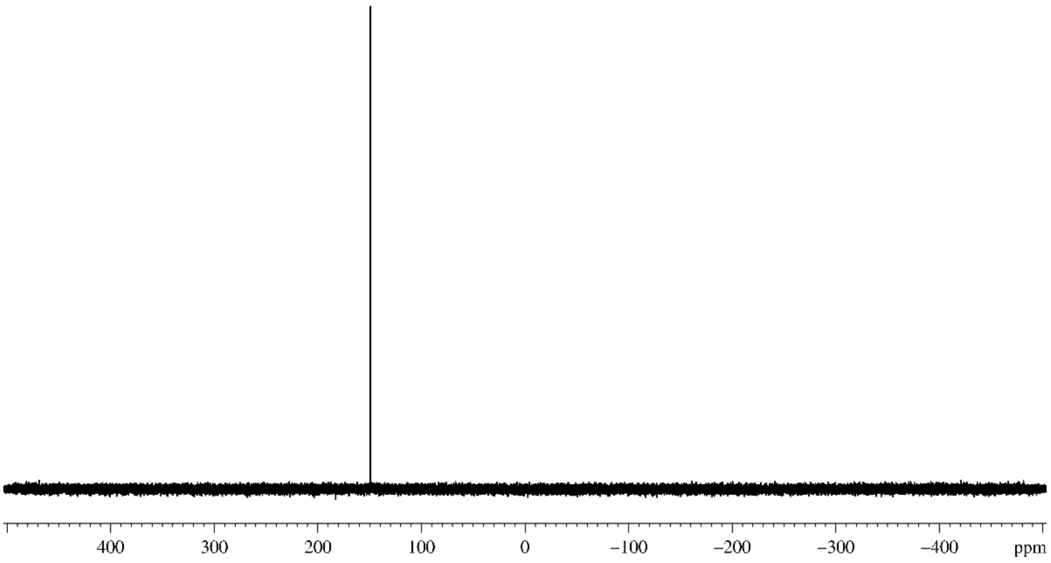

Figure S38: $^{31}P\{^{1}H\}$ NMR spectrum of [**7a**][OTf] in CD_2Cl_2 at room temperature.

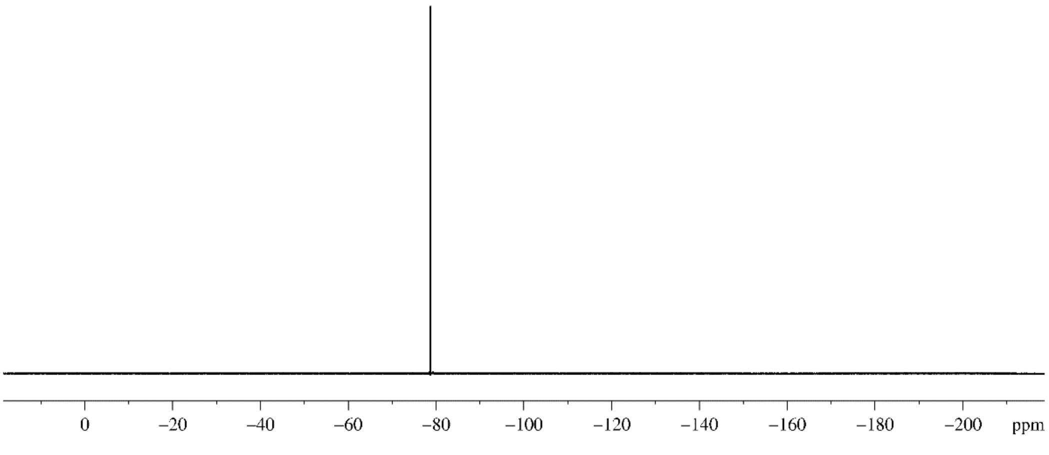

Figure S39: ^{19}F NMR spectrum of [**7a**][OTf] in CD_2Cl_2 at room temperature.

NMR spectra for [7b][OTf]

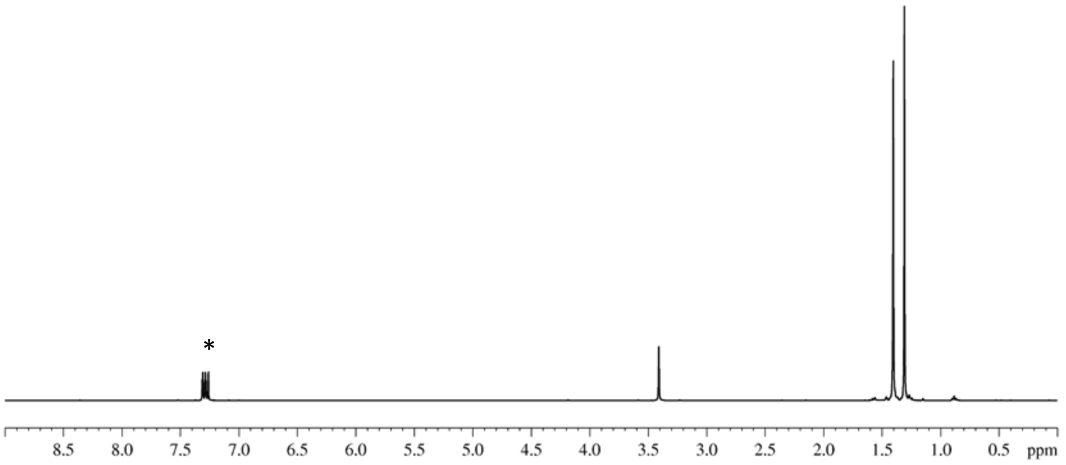

Figure S40: ^{1}H NMR spectrum of [**7b**][OTf] in $CDCl_3$ at room temperature (* denotes NMR solvent residue).

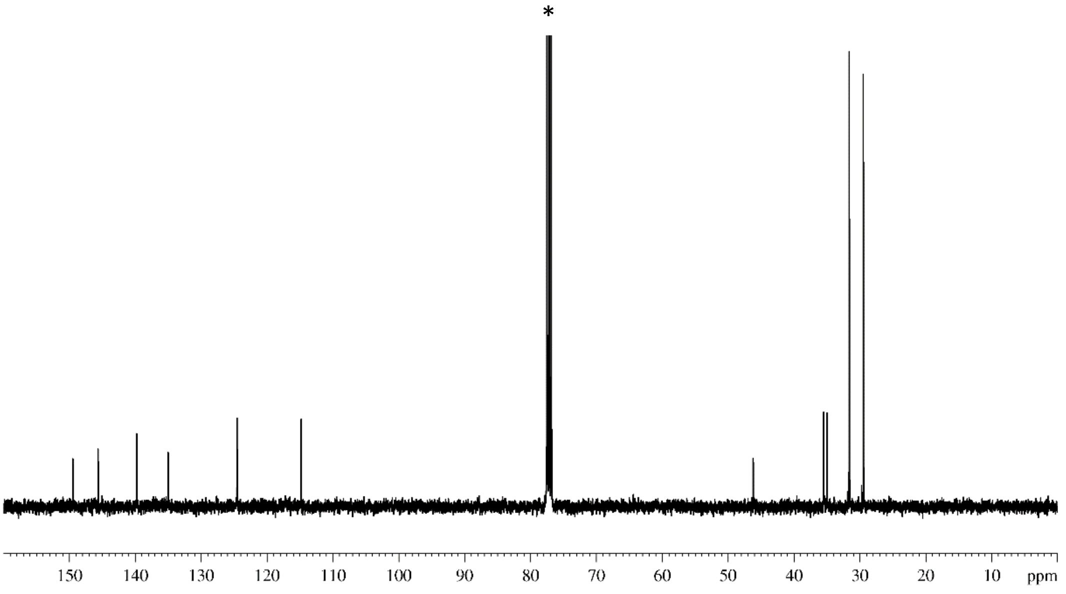

Figure S41: ^{13}C{^{1}H} NMR spectrum of [**7b**][OTf] in $CDCl_3$ at room temperature (* denotes NMR solvent residue).

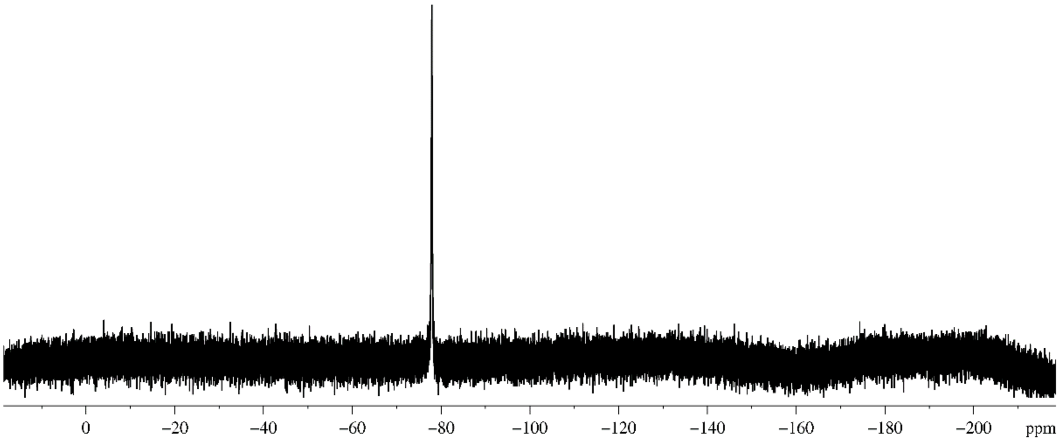

Figure S42: ^{19}F NMR spectrum of [**7b**][OTf] in $CDCl_3$ at room temperature.

NMR spectra for 8a

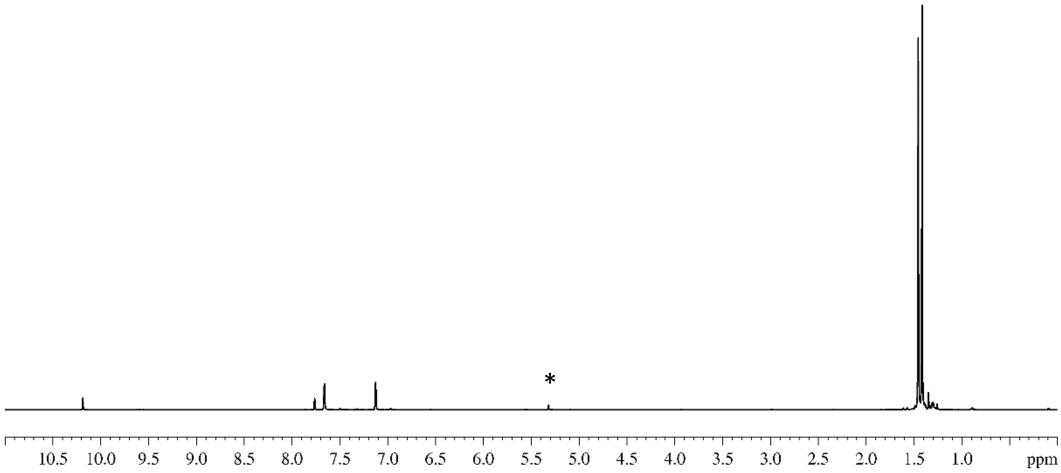

Figure S43: ^{1}H NMR spectrum of **8a** in CD_2Cl_2 at room temperature (* denotes NMR solvent residue).

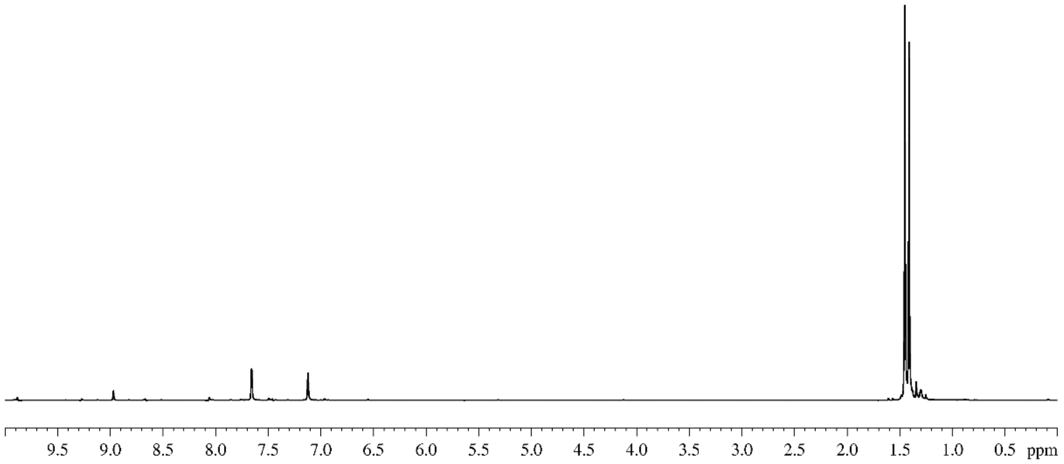

Figure S44: $^{1}H\{^{31}P\}$ NMR spectrum of **8a** in CD_2Cl_2 at room temperature.

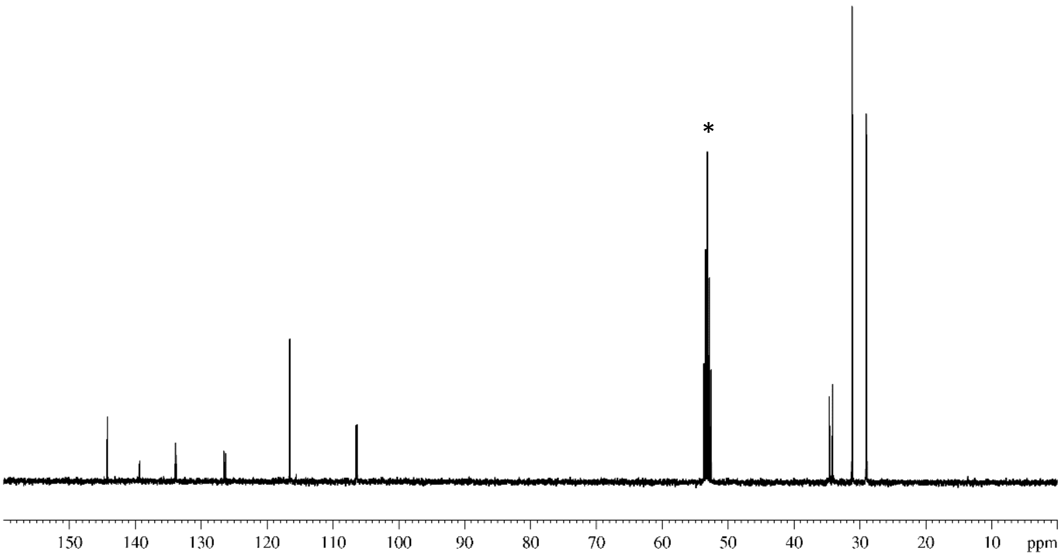

Figure S45: $^{13}C\{^{1}H\}$ NMR spectrum of **8a** in CD_2Cl_2 at room (* denotes NMR solvent residue).

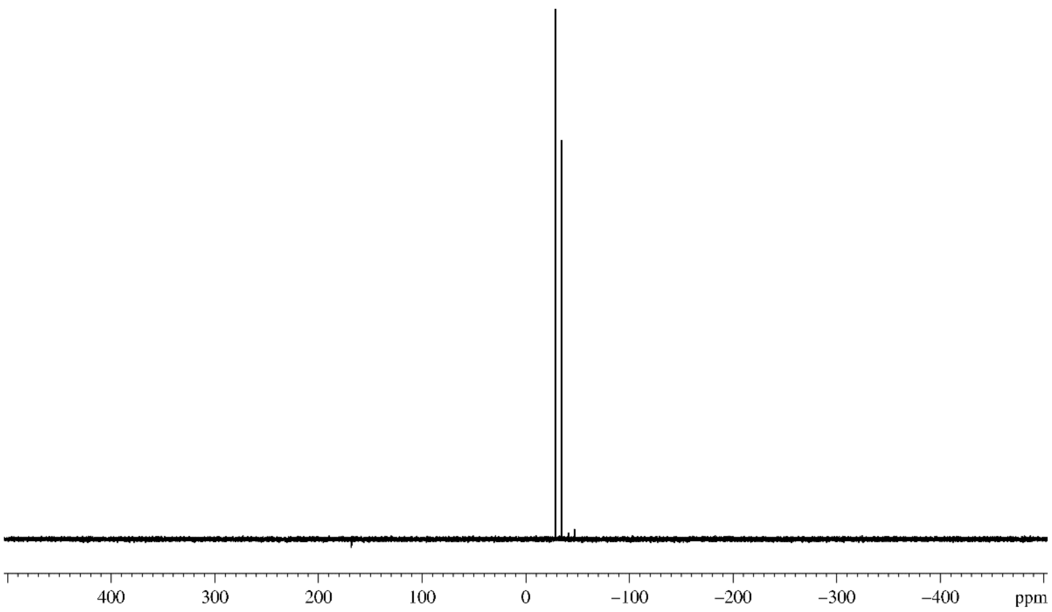

Figure S46: ^{31}P NMR spectrum of **8a** in CD_2Cl_2 at room temperature.

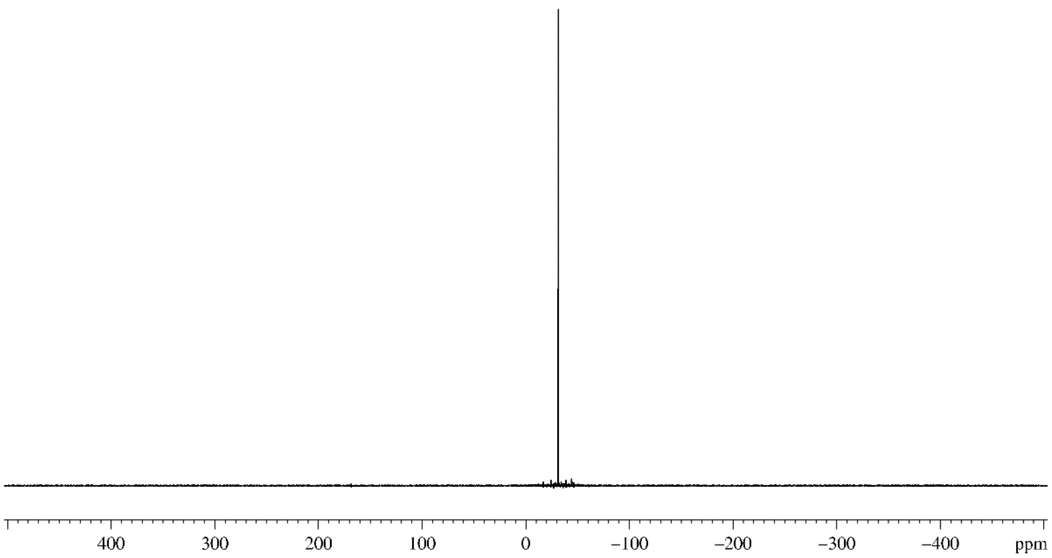

Figure S47: $^{31}P\{^{1}H\}$ NMR spectrum of **8a** in CD_2Cl_2 at room temperature.

NMR spectra for 8b

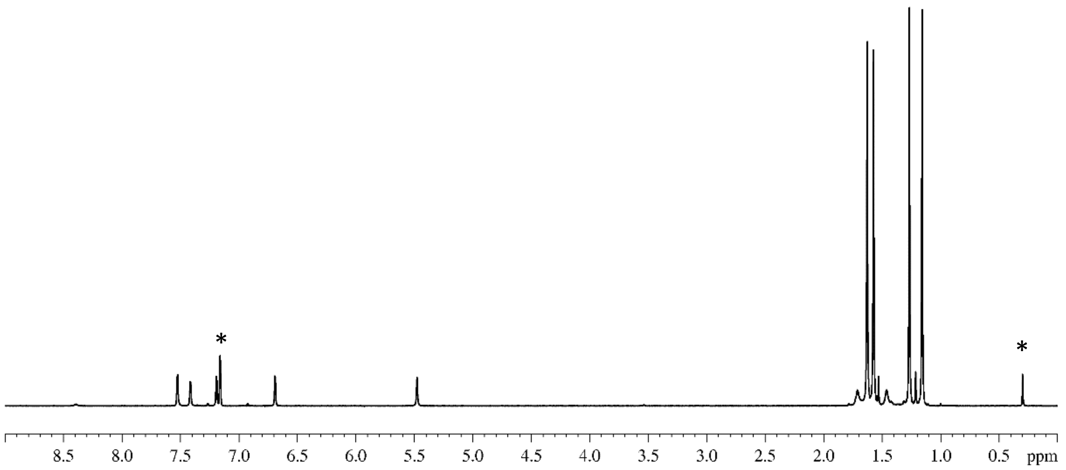

Figure S48: ^{1}H NMR spectrum of **8b** in C_6D_6 at room temperature (* denotes NMR solvent residue and silicone grease impurity).

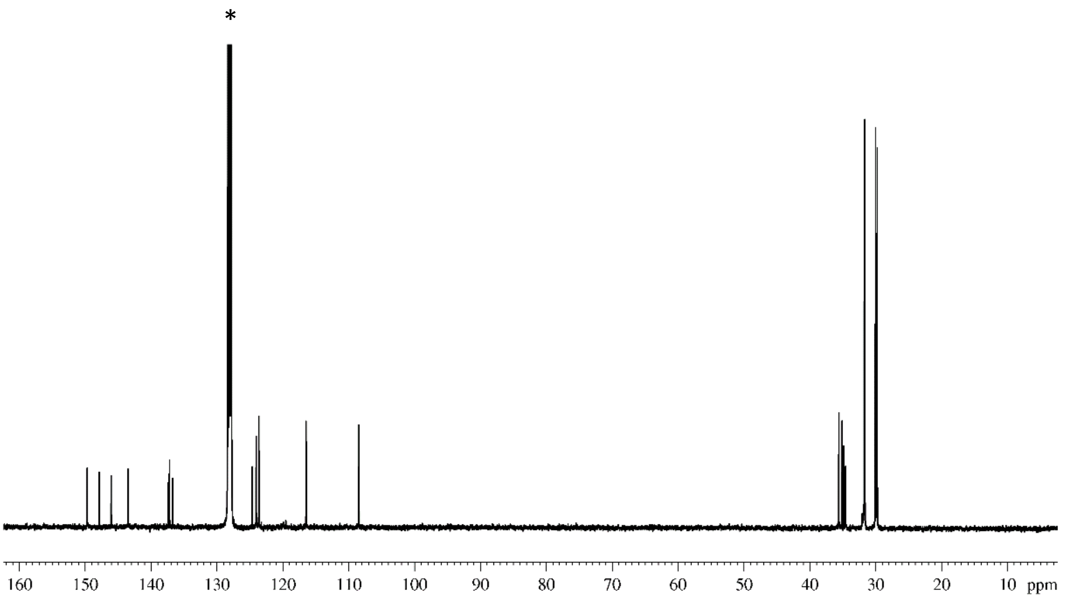

Figure S49: $^{13}C\{^{1}H\}$ NMR spectrum of **8b** in C_6D_6 at room temperature (* denotes NMR solvent residue).

NMR spectra for 9a

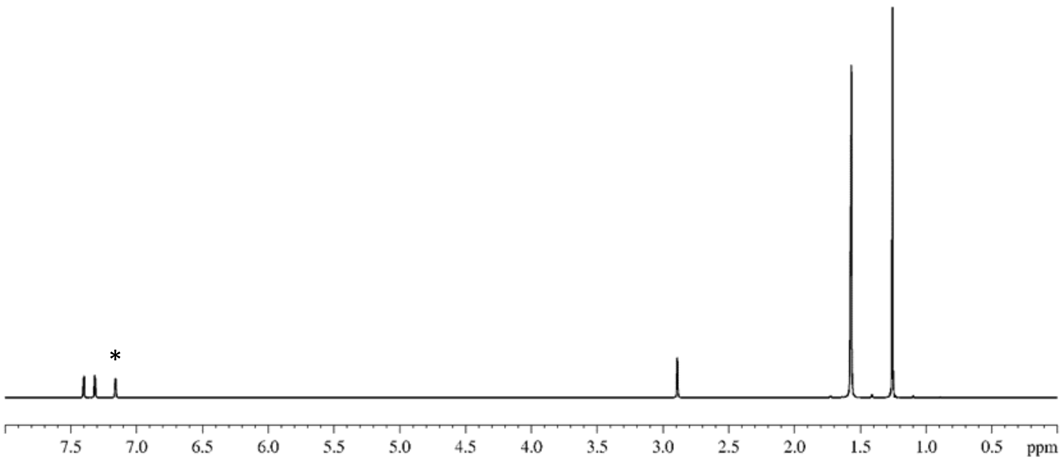

Figure S50: ^{1}H NMR spectrum of **9a** in C_6D_6 at room temperature (* denotes NMR solvent residue).

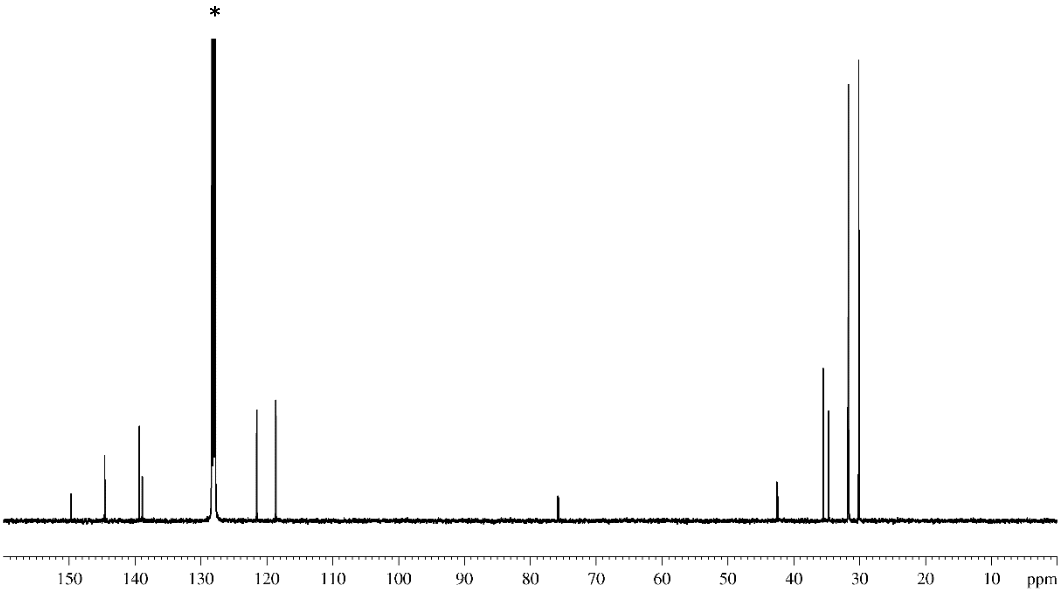

Figure S51: $^{13}C\{^{1}H\}$ NMR spectrum of **9a** in C_6D_6 at room temperature (* denotes NMR solvent residue).

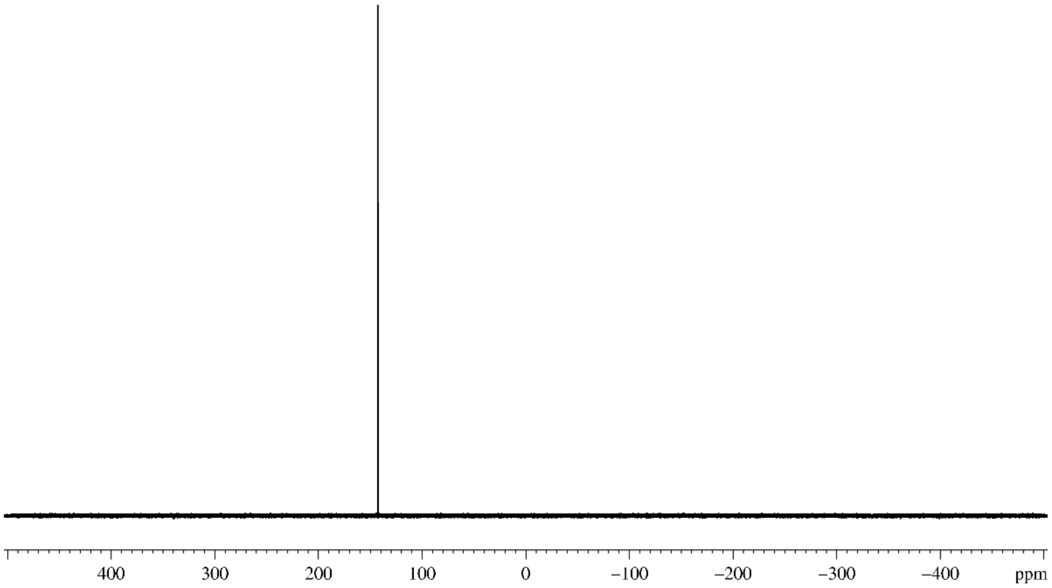

Figure S52: ^{31}P NMR spectrum of **9a** in C_6D_6 at room temperature.

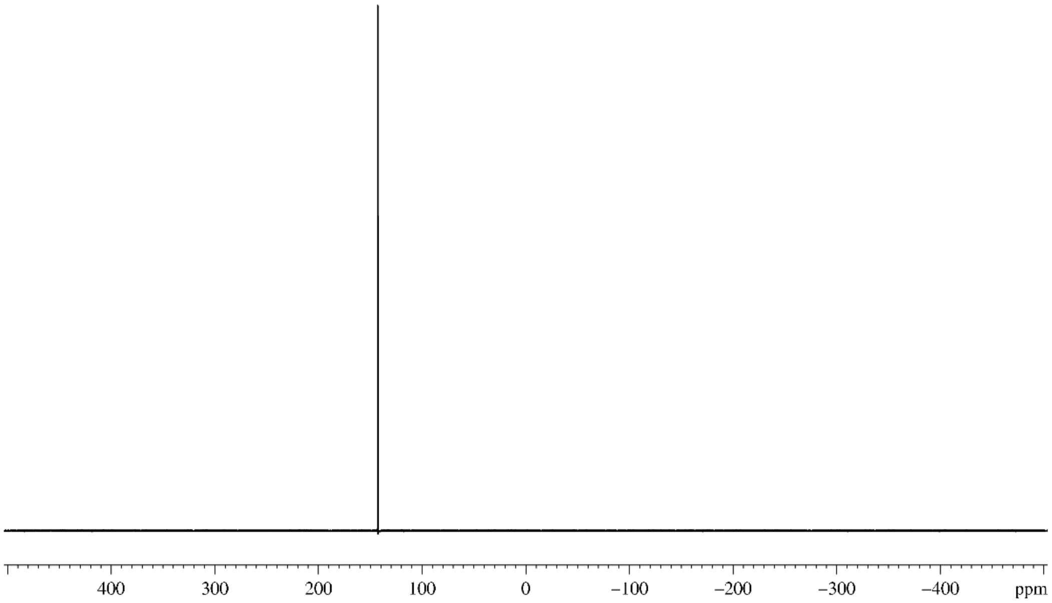

Figure S53: $^{31}P\{^1H\}$ NMR spectrum of **9a** in C_6D_6 at room temperature.

NMR Spectra for 9b

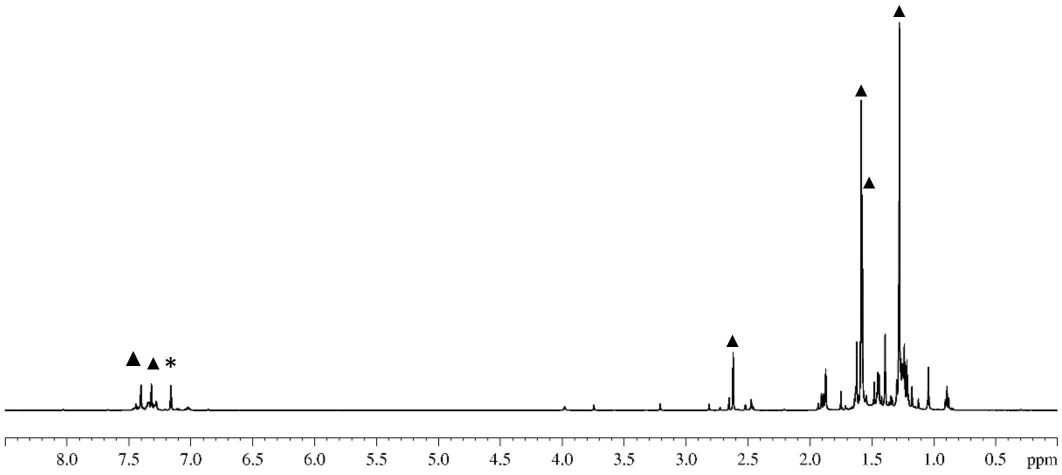

Figure S54: ^{1}H NMR spectrum of **9b** in C_6D_6 at room temperature (* denotes NMR solvent residue and ▲denotes product resonances).

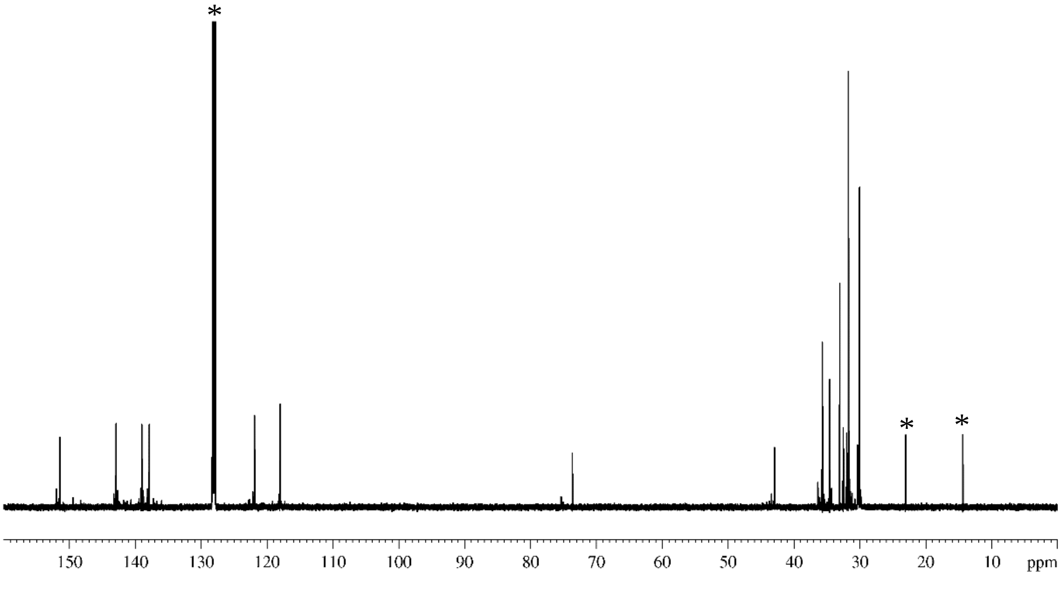

Figure S55: $^{13}C\{^{1}H\}$ NMR spectrum of **9b** in C_6D_6 at room temperature (* denotes NMR solvent residue and hexane residue).

4. MS spectra

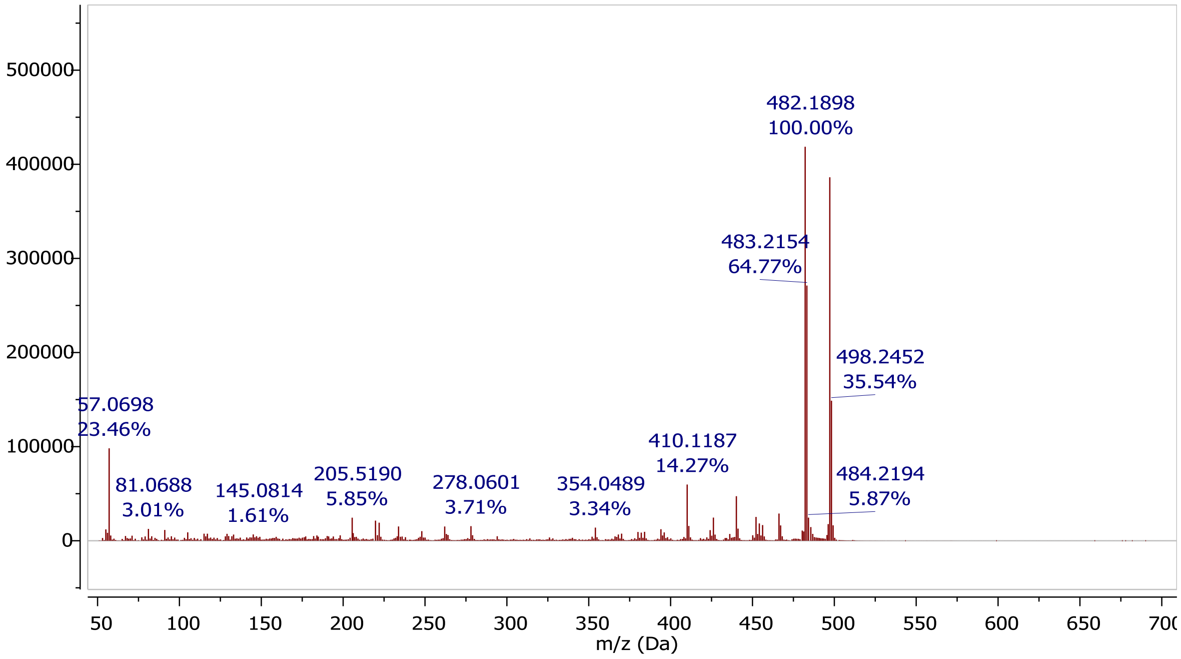

Figure S56: +ve Mode EI Mass spectrum of **1b**.

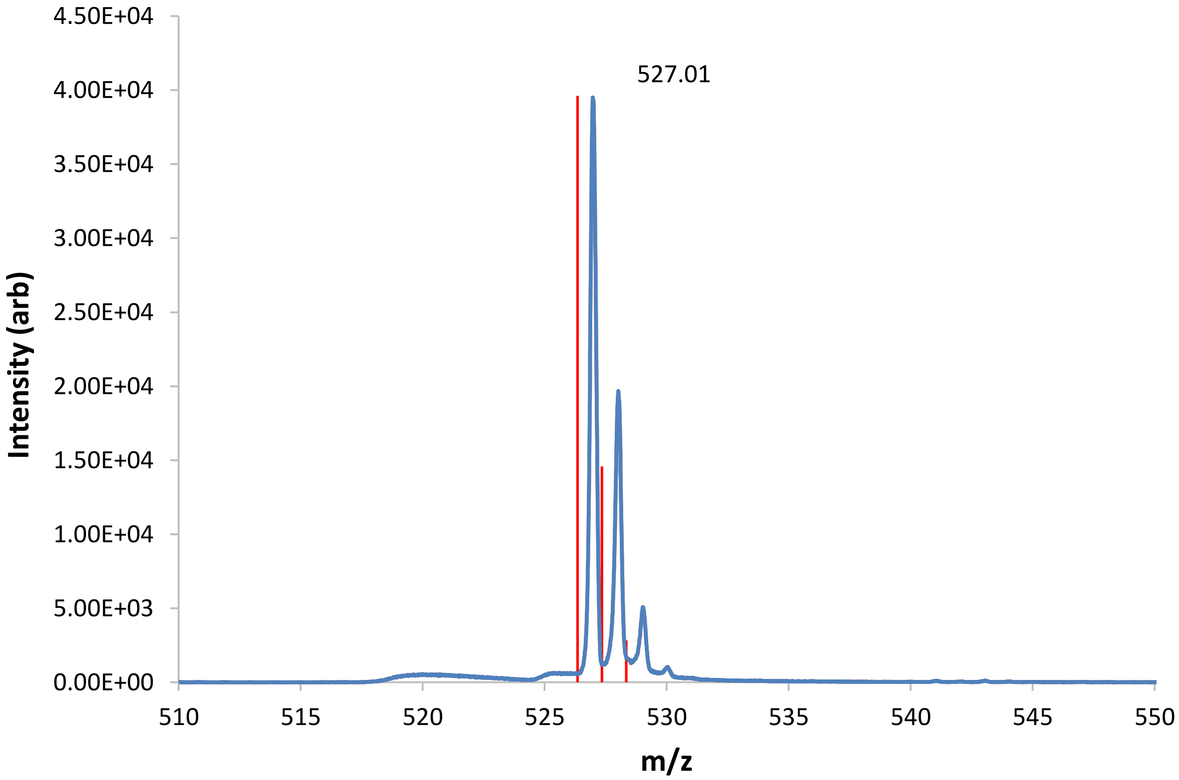

Figure S57: –ve Mode ESI Mass spectrum of **2a** recorded in DMF.

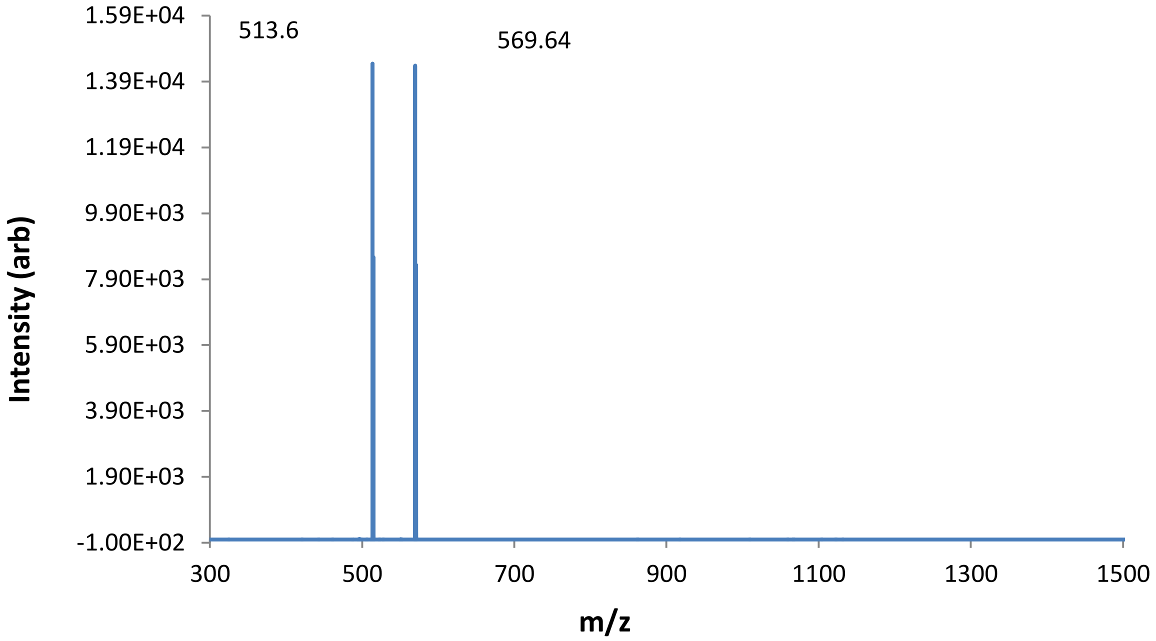

Figure S58: –ve Mode ESI Mass spectrum of **2b** recorded in DMF.

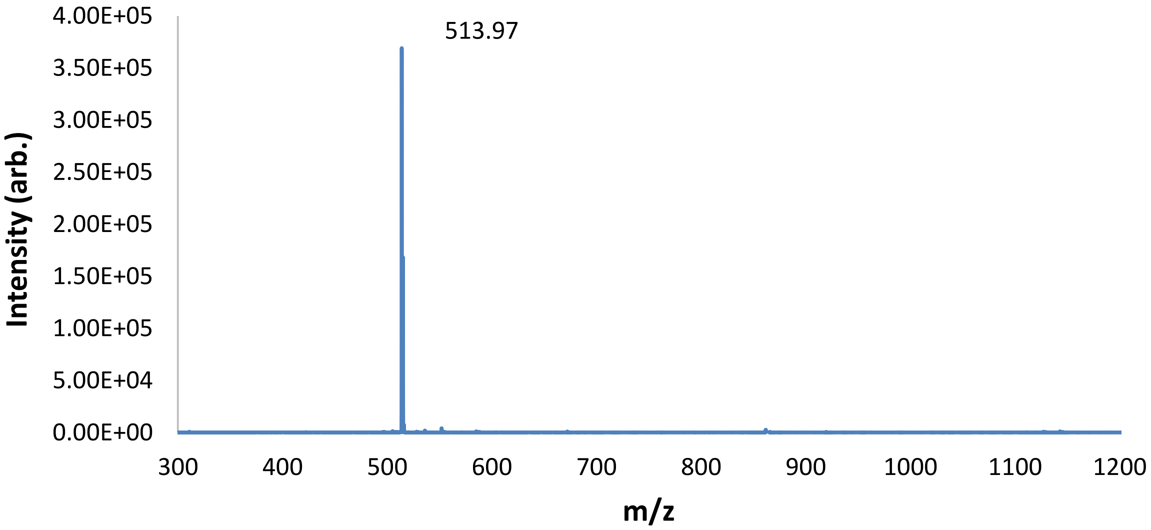

Figure S59: –ve Mode ESI Mass spectrum of **3b** recorded in DMF.

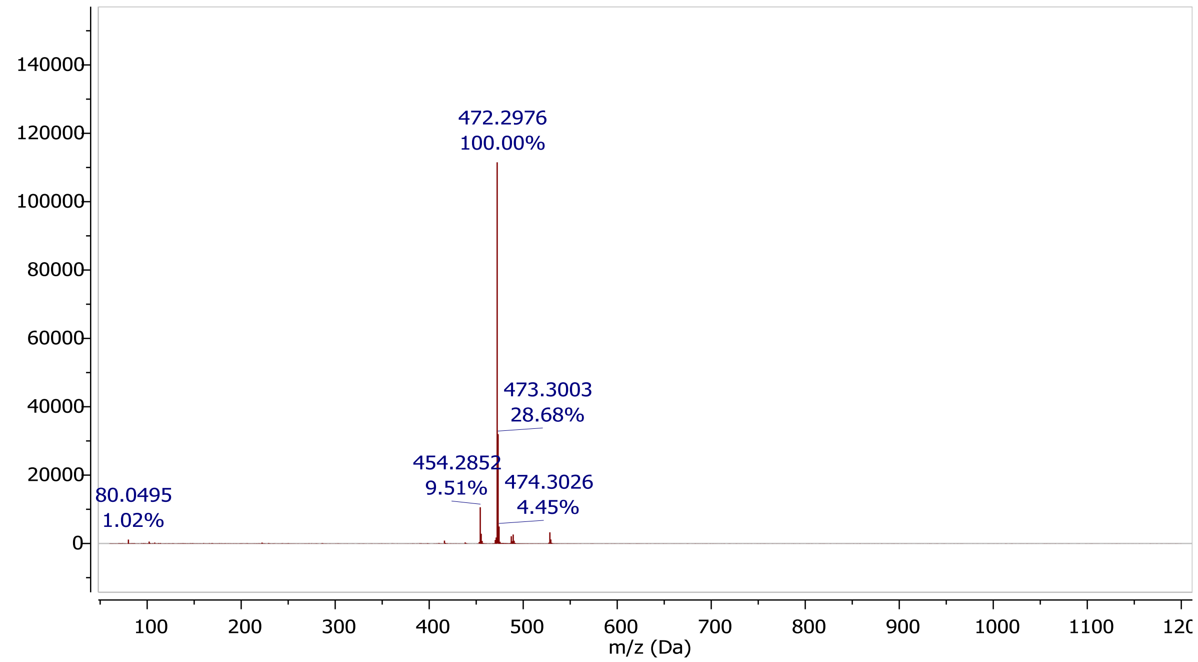

Figure S60: +ve Mode CI Mass spectrum of **4a** using NH_3 carrier gas.

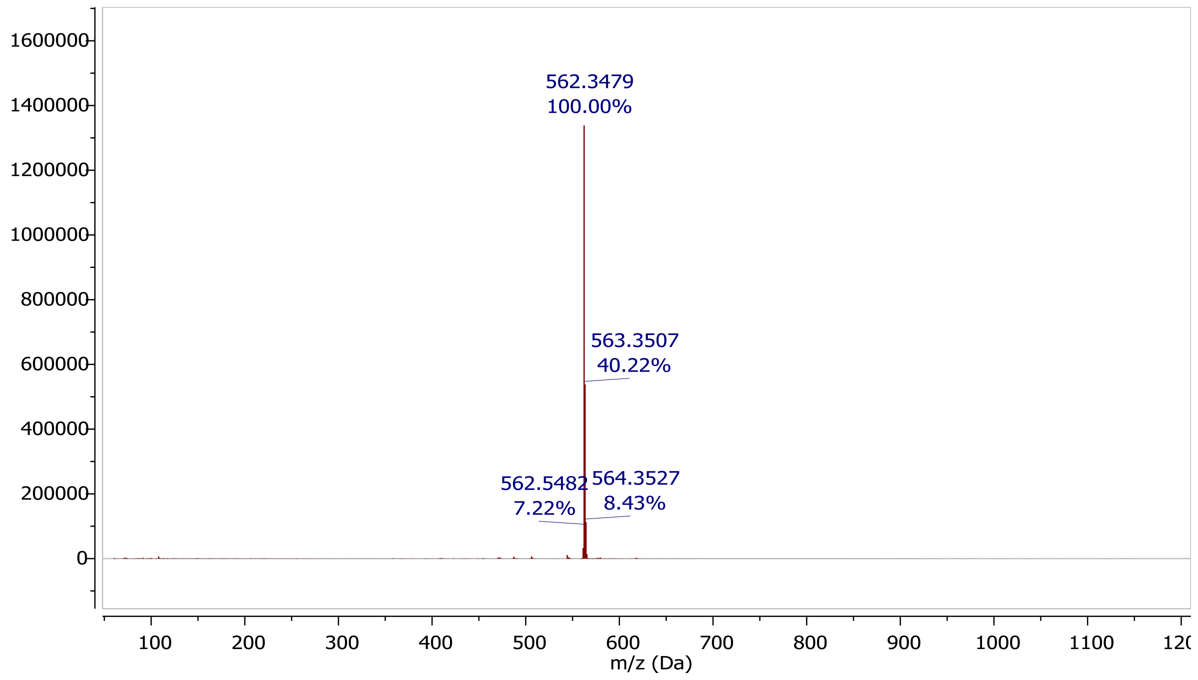

Figure S61: +ve Mode CI Mass spectrum of **5a** using NH_3 carrier gas.

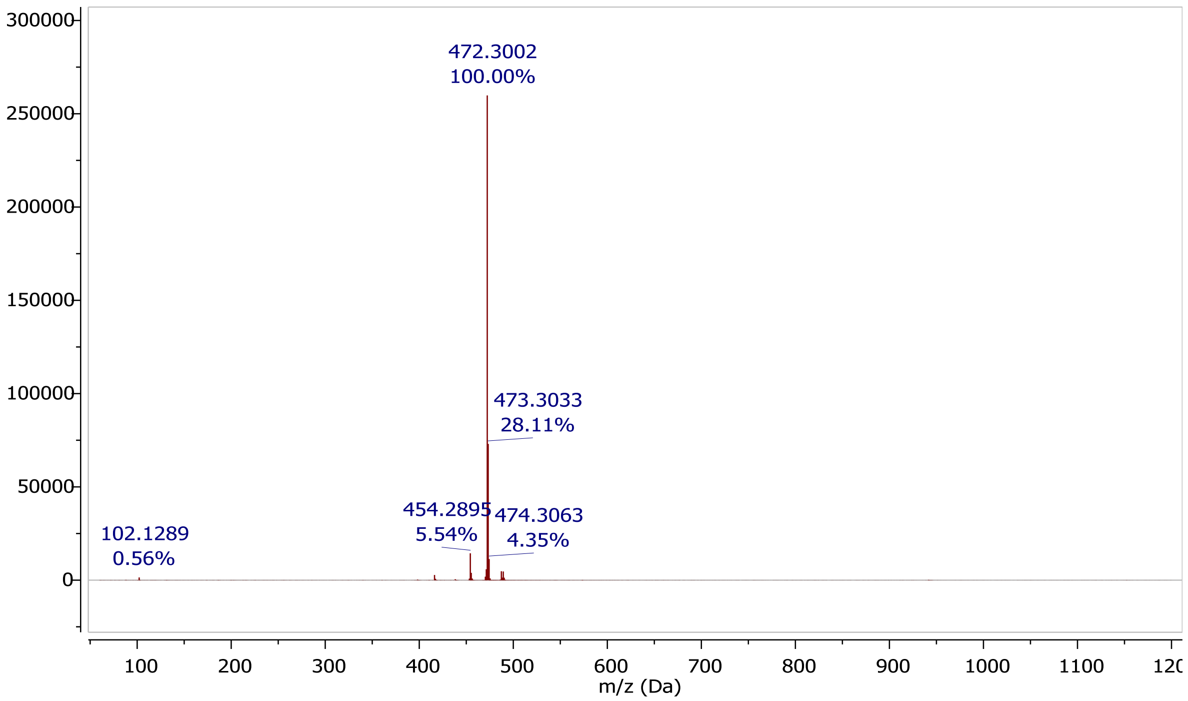

Figure S62: +ve Mode CI Mass spectrum of **6a** using NH_3 carrier gas.

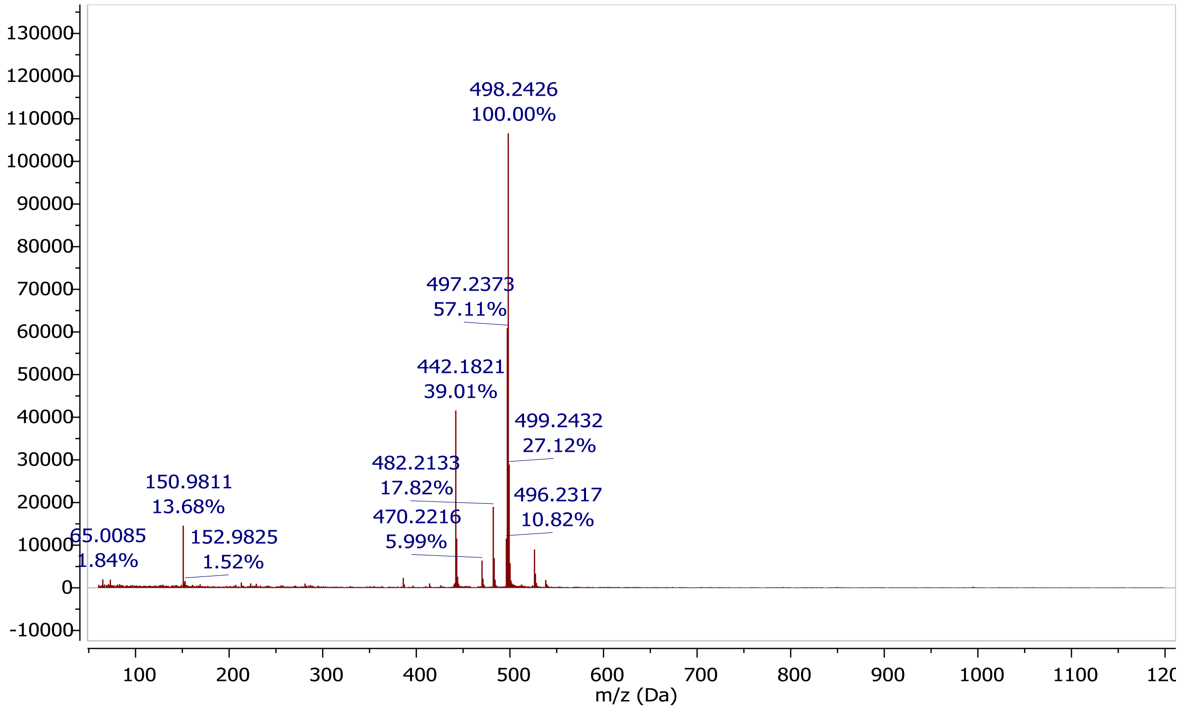

Figure S63: +ve Mode CI Mass spectrum of **6b** using CH_4 carrier gas.

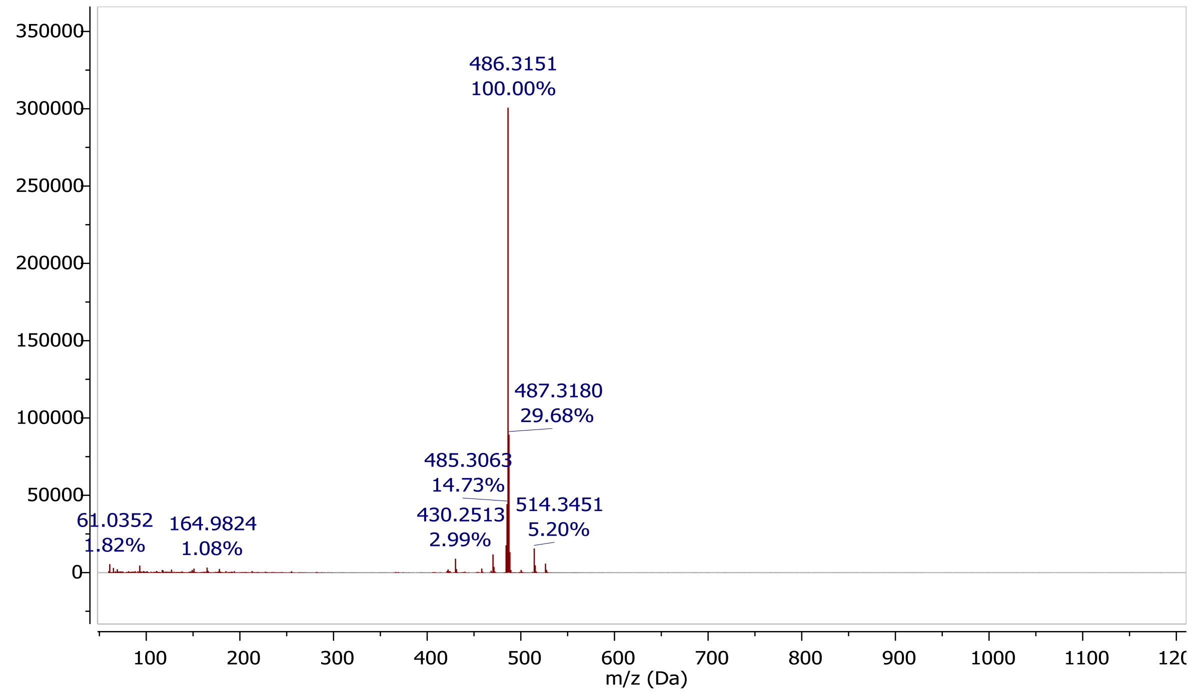

Figure S64: +ve Mode CI Mass spectrum of **7a** using NH_3 carrier gas.

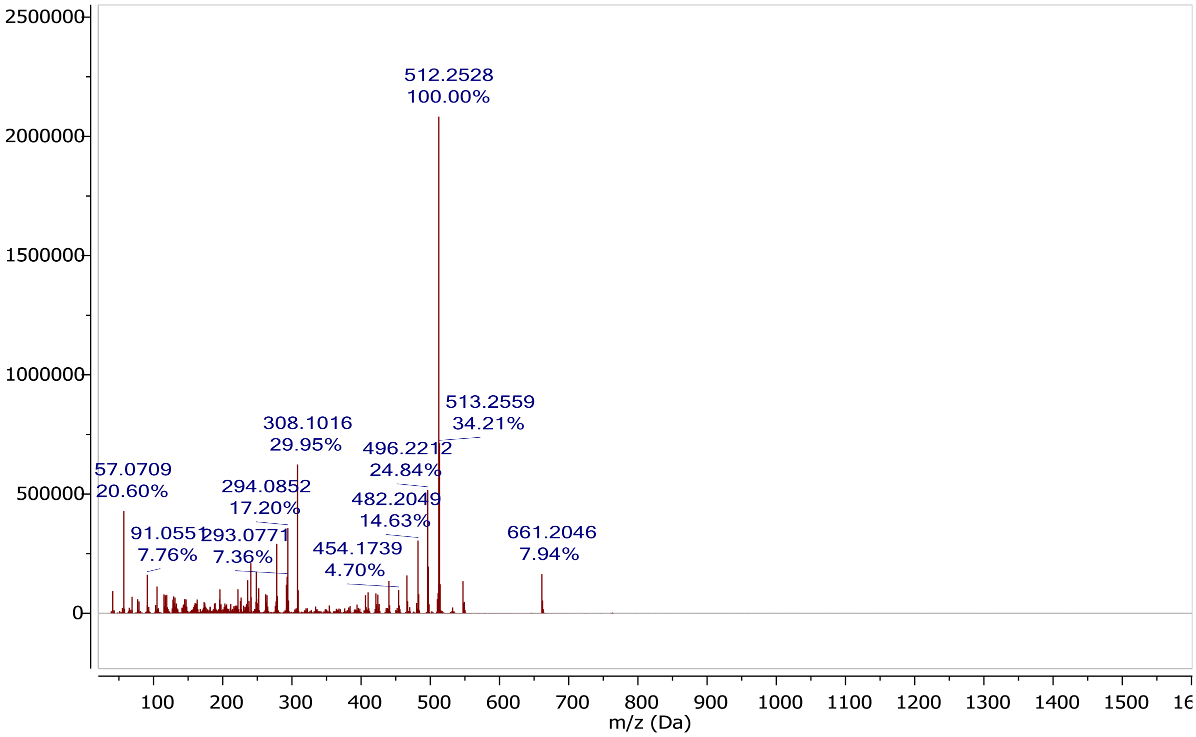

Figure S65: +ve Mode EI Mass spectrum of **7b**.

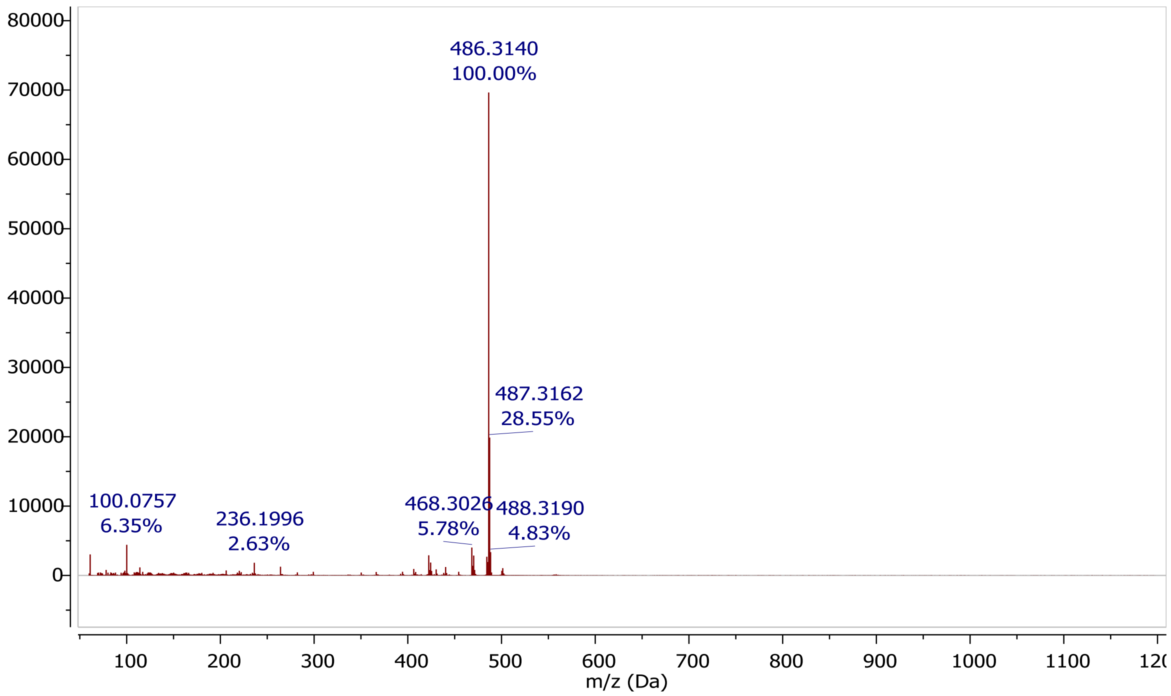

Figure S66: +ve Mode CI Mass spectrum of **9a** using NH_3 carrier gas.

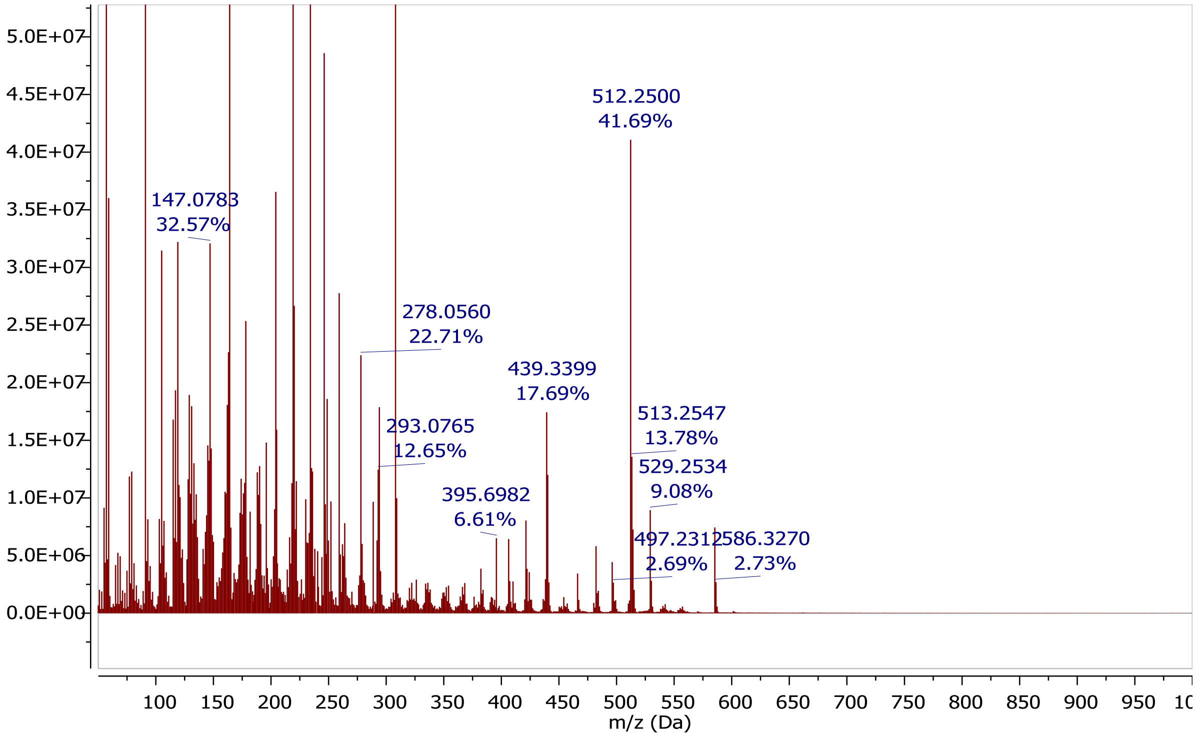

Figure S67: +ve EI Mass spectrum of **9b**.

[1] a) G. te Velde, F. M. Bickelhaupt, E. J. Baerends, C. Fonseca Guerra, S. J. A. van Gisbergen, J. G. Snijders, T. Ziegler, *J. Comput. Chem.* **2001**, *22*, 931−967; b) C. Fonseca Guerra, J. G. Snijders, G. te Velde, E. J. Baerends, *Theor. Chem. Acc.* **1998**, *99*, 391−403; c) ADF2014.01, SCM, Theoretical Chemistry, Vrije Universiteit: Amsterdam, The Netherlands, http://www.scm.com.

[2] A. D. Becke, *Phys. Rev. A* **1988**, *38*, 3098–3100.

[3] J. P. Perdew, *Phys. Rev. B* **1986**, *33*, 8822–8824.

[4] S. Grimme, J. Anthony, S. Ehrlich, and H. Krieg, *J. Chem. Phys.* **2010**, *132*, 154104–154119.

[5] L. Versluis, T. Ziegler, *J. Chem. Phys.* **1988**, *88*, 322−328.
